# The genetic basis of sex determination in grapes

**DOI:** 10.1038/s41467-020-16700-z

**Published:** 2020-06-09

**Authors:** Mélanie Massonnet, Noé Cochetel, Andrea Minio, Amanda M. Vondras, Jerry Lin, Aline Muyle, Jadran F. Garcia, Yongfeng Zhou, Massimo Delledonne, Summaira Riaz, Rosa Figueroa-Balderas, Brandon S. Gaut, Dario Cantu

**Affiliations:** 1 Department of Viticulture and Enology, University of California Davis, Davis, CA 95616 USA; 2 Department of Ecology and Evolutionary Biology, University of California Irvine, Irvine, CA 92697 USA; 3 Dipartimento di Biotecnologie, Università degli Studi di Verona, 37134 Verona, Italy

**Keywords:** Sexual selection, Agricultural genetics, Plant genetics, Plant reproduction

## Abstract

It remains a major challenge to identify the genes and mutations that lead to plant sexual differentiation. Here, we study the structure and evolution of the sex-determining region (SDR) in *Vitis* species. We report an improved, chromosome-scale Cabernet Sauvignon genome sequence and the phased assembly of nine wild and cultivated grape genomes. By resolving twenty *Vitis* SDR haplotypes, we compare male, female, and hermaphrodite haplotype structures and identify sex-linked regions. Coupled with gene expression data, we identify a candidate male-sterility mutation in the *VviINP1* gene and potential female-sterility function associated with the transcription factor *VviYABBY3*. Our data suggest that dioecy has been lost during domestication through a rare recombination event between male and female haplotypes. This work significantly advances the understanding of the genetic basis of sex determination in *Vitis* and provides the information necessary to rapidly identify sex types in grape breeding programs.

## Abstract (web summary)

Grapevine is one of a few ancestrally dioecious crops that are reverted to hermaphroditism during domestication. Here, the authors identify candidate genes related to male- and female-sterility in grapes and describe the genetic process that led to hermaphroditism during domestication.

## Introduction

Plant species possess a variety of mating systems. Some are monoecious, with separate male and female flowers on the same plant, and others are hermaphroditic, with bisexual flowers. Occasionally plants have separate male and female individuals, a mating system called dioecy. Dioecy ensures outcrossing, but it occurs in only 5–6% of angiosperms^1,2^. Despite its rarity, dioecy is widespread phylogenetically, suggesting it has evolved independently on multiple occasions.

Dioecy has been the focus of numerous evolutionary and genetic studies, both because of its multiple evolutionary origins and because several economically important crops are dioecious. A common hypothesis about the origin of dioecy is the two-locus model, which requires that dioecy evolved from an hermaphroditic ancestor in two steps^1,3,4^. The first step includes a recessive mutation that interrupts male function. Individuals with a homozygous male-sterility mutation retain only female function, and a population with this mutation contains both females and hermaphrodites (gynodioecy). The second step requires a dominant mutation that suppresses female function, leading to males. This two-locus system can maintain separate sexes only if the two loci are completely linked, because recombination between them could restore hermaphrodites^1,5^. Support for the two-locus model has been found in several species, including papaya^6^ (*Carica papaya*), strawberry^7^ (*Fragaria virginiana*), *Silene latifolia*^8^, *Actinidia* spp.^9^ and grapes^10^ (*Vitis* spp.). However, the sterility mutations that cause dioecy have not been identified completely in any species^11^. Thus far, the best candidates are from asparagus and kiwifruit^9,12,13^. In asparagus, for example, females lack a gene associated with tapetal development^13^ and mutant males without a putative female-suppressor gene revert to hermaphrodites^12^.

**Fig. 1:**
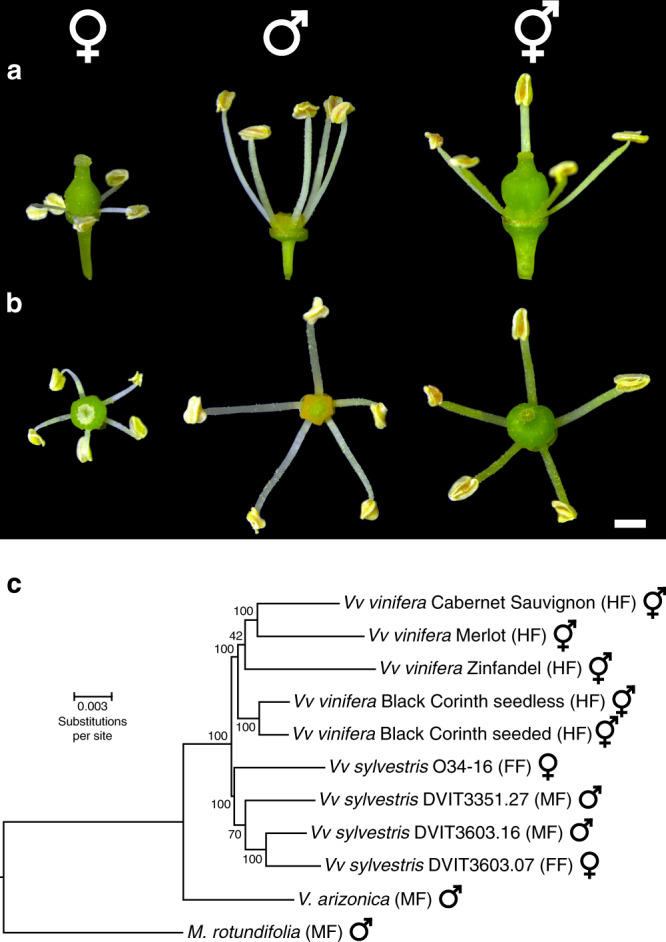
The morphology of flower sexes in grapes and a phylogenetic analysis of wild and cultivated species. Side view (**a**) and top view (**b**) of dioecious *Vv sylvestris* female O34-16 (left), male DVIT3351.27 (middle), and of hermaphrodite *Vv vinifera* Chardonnay (right). Scale bar = 1 mm. **c** A phylogenetic tree predicted from whole-genome proteome orthology separates species by taxonomy and not by sex genotype. *M. rotundifolia* is an outgroup to the *Vitis* ingroup^23^. For each individual, the genotype of the sex-determining region is indicated in parentheses followed by its corresponding sex type. The symbols ♀, ♂ and  represent female, male, and hermaphrodite individuals, respectively. Numbers associated with nodes reflect bootstrap values (see “Methods”). Scale bar is in the unit of the number of substitutions per site.

Here we study the sex-determining region (SDR) within the genus *Vitis*. All ~70 wild *Vitis* species are dioecious^14^, suggesting that dioecy has been conserved since the origin of the genus. One *Vitis* species, the cultivated grapevine (*Vitis vinifera* ssp. *vinifera*; hereafter *Vv vinifera*), has reverted to hermaphroditism, even though its wild ancestor *Vitis vinifera* ssp. *sylvestris* (hereafter *Vv sylvestris*) is dioecious. This shift of mating system occurred during domestication ~8000 years ago^15^, perhaps following a rare recombination event between male (M) and female (F) haplotypes^10,11^. Therefore, *Vitis* spp. have individuals of three types (Fig. 1a, b): (i) males with flowers that have reduced pistils, with neither stigma nor style development, (ii) females with flowers containing reflexed anthers and stamens that release sterile pollen grains^16^, and (iii) hermaphrodites within *Vv vinifera*, which have perfect flowers with functional pistils and stamens that bear fertile pollen. The three types are determined by the genotype at the SDR. Males are heterozygous for male and female haplotypes (MF), females are homozygous (FF), and cultivated *Vv vinifera* hermaphrodites are either homozygous for hermaphrodite haplotypes (HH) or heterozygous (HF).

Previous genetic and genomic studies have identified the approximate boundaries of the SDR in grapes^10,17,18^. In *Vitis* spp., the SDR maps genetically to ~150 kbp of chromosome 2 that contains between 15 and 20 genes^10,18^. Polymorphisms within the region have high linkage disequilibrium in *Vv sylvestris*, suggesting low or no recombination between M and F haplotypes^10^. It has been hypothesized that this region contains the recessive male-sterility and dominant female-sterility alleles predicted by the two-locus model, and their identification has been attempted by comparative gene expression analyses^10,19^. One candidate gene, the adenine phosphoribosyltransferase gene *VviAPT3*, is expressed in the carpel primordial of male plants, suggesting a role in pistil abortion^20^.

Until recently, a major limitation in the study of *Vitis* sex determination has been that the *Vv vinifera* reference genome represented only a partially assembled F haplotype^21^. More recent work has resolved the partial sequence of four SDR haplotypes, including three H and one F haplotypes^22^. Yet, despite substantial progress, our understanding of the SDR and the potential determinants of sex have been hampered by the absence of information from M haplotypes.

To fill this gap, we report nine phased diploid genomes of cultivated hermaphrodites, and wild male and female grapes. All of these genomes are based on high-coverage, Pacific Biosciences (PacBio) long-read sequencing. For each genome, haplotypes within the genetically defined SDR have been curated manually, and transcripts expressed from the region have been measured during early and late stages of flower development in male, female, and hermaphrodite plants. With these extensive sequence and expression data, we compare the F, H, and M haplotypes to better define the SDR, identify candidate sex-determining genes, and assess whether H haplotypes owe their origin to a recombination event.

## Results

### Sex-specific haplotypes are conserved throughout *Vitis* spp

We sequenced and assembled the complete genomes of eight *Vitis* accessions, including three hermaphrodite *Vv vinifera* cultivars (Merlot, Black Corinth seedless and Black Corinth seeded), four *Vv sylvestris* accessions (two females and two males), and one male *V. arizonica*. In addition, the genome of one male *Muscadinia rotundifolia* was sequenced as a dioecious outgroup to *Vitis spp*.^23^ (Fig. 1c). Each genome was based on Single Molecule Real Time (SMRT) DNA sequencing and de novo assembled with FALCON-Unzip^24^, which produces partially phased diploid genomes. We also included two publicly available genomes from the Cabernet Sauvignon and Zinfandel grape cultivars, both of which were sequenced and assembled with the same approach^24–26^. All diploid assemblies were highly contiguous and covered approximately twice the expected haploid genome size^21^ (Supplementary Data 1). The Cabernet Sauvignon assembly was improved further with HiC proximity-based ligation, optical mapping, and multiple scaffolding methods, resulting in two phased copies, hap1 and hap2, of all 19 chromosomes.

The SDR was first identified by aligning primer sequences of sex-linked markers^10,17^ to chromosome 2 of the Cabernet Sauvignon hap1 reference. Protein-coding sequences of the Cabernet Sauvignon hap1 SDR were then aligned to the other ten genome assemblies to identify orthologous regions. Next, we manually annotated the SDR of each haplotype in each genome and assigned a sex type (M, F or H) based on known genotypes (Fig. 1c) and known H vs. F haplotype structure^22^. By these means, we resolved a dataset of 20 *Vitis* SDR haplotypes (5 H, 12 F, and 3 M haplotypes) and the M and F haplotypes of *M. rotundifolia* (Fig. 1c).

**Fig. 2:**
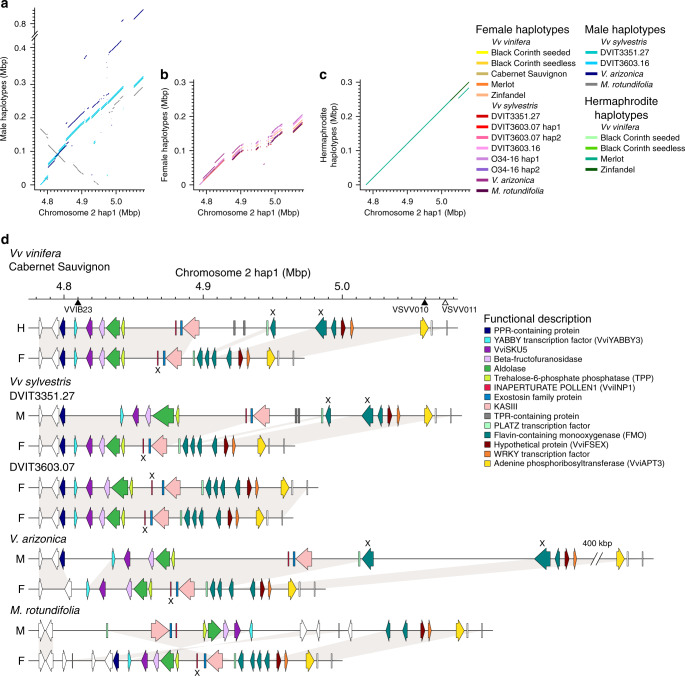
Sex-linked structural variants and their impact on gene content. Whole-sequence alignments of the sex-determining region (SDR) in M (**a**), F (**b**), and H (**c**) haplotypes against the *Vv vinifera* Cabernet Sauvignon chromosome 2 hap1 (H) reference. The figures illustrate that M haplotypes (**a**) tend to be longer than either F (**b**) or H (**c**) haplotypes and there is a large inversion in the M haplotype of *M. rotundifolia*. **d** Schematic representations of the SDR in four of the 11 genomes analyzed for this study. From top to bottom, the figure illustrates the haplotypes of hermaphrodite *Vv vinifera* Cabernet Sauvignon (HF), male *Vv sylvestris* DVIT3351.27 (MF), female *Vv sylvestris* DVIT3603.07 (FF), male *V. arizonica* (MF), and male *M. rotundifolia* (MF). Each haplotype is annotated with arrows and rectangles to depict annotated genes. White arrows depict genes that are not sex-linked. Genes affected by nonsense mutations are indicated with an X. The scale above the haplotypes denotes the position on the Cabernet Sauvignon reference. The filled, black triangles on this scale mark the position of the sex-linked genetic markers VVIB23^17^ and VSVV010^10^; the white-filled triangle represents the amplicon VSVV011^10^, which is not linked to the SDR. Source data are provided as a Source Data file.

All SDR haplotypes were aligned to the Cabernet Sauvignon hap1 H haplotype to assess structural differences and to identify sex-specific features (Fig. 2; Supplementary Fig. 1). There were obvious differences in length among the sexes. Overall, the haplotypes ranged in size from ~171.6 to ~837.4 kbp (Fig. 2a–c), but F haplotypes (181.4 ± 10.2 kbp) were significantly shorter than M (425.9 ± 274.6 kbp; Mann−Whitney test, *P* value = 0.0008403) and H (289.2 ± 7.4 kbp; *P* value = 0.0002334) haplotypes. These length differences reflect the presence of sex-linked structural variants (SVs; >50 bp). For example, the 12 *Vitis* F haplotypes shared eight large deletions relative to the H haplotype of Cabernet Sauvignon, encompassing a total length of 117.4 kbp (Fig. 2b). Most (62.9%) of these F-linked deletions were composed of transposable elements (TE), including LTR, Gypsy, Copia and MuDR elements. Similarly, the three M haplotypes shared two large SVs relative to the H reference, including a 22.6 kbp insertion at position 4,802,134 and a 30 kbp deletion from positions 5,021,983 to 5,052,079. In addition, the *V. arizonica* M haplotype had a unique ~400 kbp insertion, and the M haplotype of *M. rotundifolia* contained an inversion that encompassed 57% of the SDR (Fig. 2a). The SDR haplotypes varied in gene content among sexes. While all 20 *Vitis* SDR haplotypes shared 13 SDR genes, two SVs altered gene content. As previously observed in Zhou et al.^22^, F haplotypes had an SV relative to H and *Vv sylvestris* M haplotypes that deleted two genes encoding TPR-containing proteins. In addition, a *FLAVIN-CONTAINING MONOOXYGENASE* (*FMO*) gene was absent from H and M haplotypes relative to F haplotypes. The overall structure and gene content were well-conserved between *Vitis* and *M. rotundifolia* species. Despite a large inversion, gene content and order in the M haplotype of *M. rotundifolia* was similar to *Vitis* M haplotypes, and the F haplotype of *M. rotundifolia* was identical in gene content and order to *Vitis* F haplotypes (Fig. 2d). Finally, H haplotypes were similar in structure to F haplotypes within the first 60 kbp of the SDR, but they were more similar in structure to M haplotypes downstream of this region (Fig. 2d).

### Sex-linked polymorphisms affect protein sequences

**Fig. 3:**
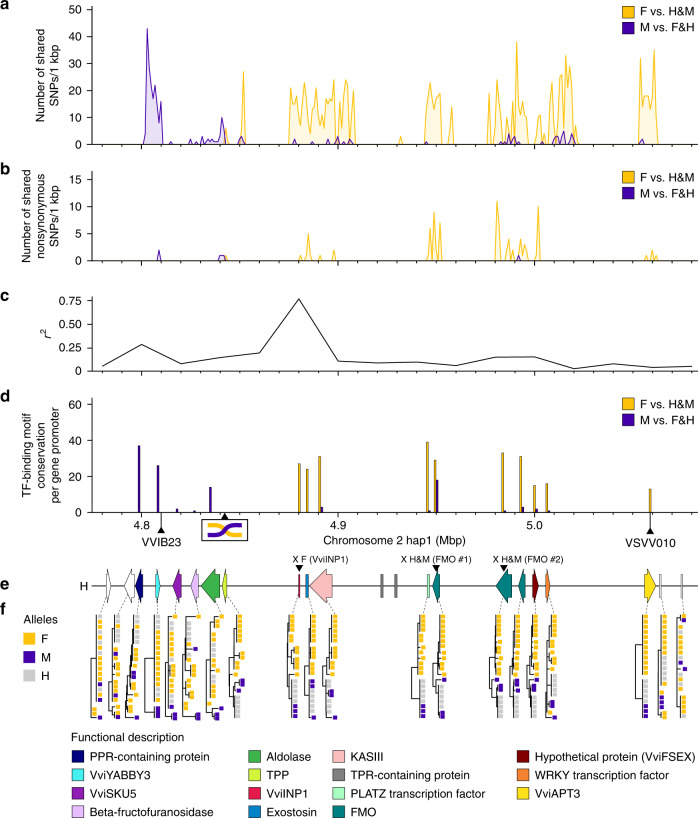
Sex-linked polymorphisms along the sex-determining region in *Vitis spp*. The number of all (**a**) and nonsynonymous sex-linked SNPs (**b**) per kbp across the sex-determining region (SDR). SNPs were identified by aligning all *Vitis* haplotypes to *Vv vinifera* Cabernet Sauvignon hap1 (H). Only SNPs strictly (100%) linked to one sex type were plotted. **c** Linkage disequilibrium (LD) across the region represented as the median of the squared correlation coefficients (*r*^2^) between all pairs of SNPs calculated within 20 kbp windows. **d** TF-binding motif conservation per gene promoter detected in the SDR. The *x*-axis denotes the location on the Cabernet Sauvignon hap1 (H) SDR, and the two black triangles along this axis mark the position of the genetic markers VVIB23^17^ and VSVV010^10^ that are closely linked to the SDR. The potential position of the recombination event responsible for the reversion to hermaphroditism in domesticated *Vv vinifera* is also indicated. **e** Gene composition of the H haplotype of *Vv vinifera* Cabernet Sauvignon hap1. Genes are colored as in Fig. 2d and white-colored genes are not sex-linked. Genes affected by nonsense mutations are indicated with an X followed by the affected haplotype and the gene name in parentheses. **f** Neighbor-joining clustering of the protein sequences encoded by each gene of the SDR. Yellow, purple and gray colors represent F, M and H haplotypes, respectively. Source data underlying Figs. 3a–d, f are provided as a Source Data file.

We used our sequence alignments to the Cabernet Sauvignon H haplotype to identify SNPs that associate perfectly with sex among *Vitis* spp. All of the F- and M-associated polymorphisms were found from positions 4,801,876 to 5,061,548 on chromosome 2 of the Cabernet Sauvignon hap1 reference (Fig. 3a; Supplementary Data 2; Supplementary Fig. 2), which further confirms and delimits the SDR^10,18^. In total, 1275 SNPs were shared by all 12 *Vitis* F haplotypes vs. H and M haplotypes, and 270 SNPs were shared by all three M haplotypes vs. F and H haplotypes. Interestingly, M-linked SNPs were densest in the first 8 kbp of the SDR (176 SNPs, from 4,801,876 to 4,809,592), and the first F-linked SNP was ~40 kbp downstream from the dense cluster of M-linked SNPs (4,842,196; Fig. 3a). Sex-specific SNP distributions were largely consistent when including *M. rotundifolia* haplotypes in the comparison, though the number of sex-specific SNPs decreased due to divergence between the two genera (Supplementary Fig. 3).

Many of the sex-linked SNPs altered amino acids (Fig. 3b). Altogether, we found six M-linked nonsynonymous SNPs: two in the YABBY transcription factor (TF)-coding gene *VviYABBY3*^27^, two in an aldolase-coding gene, one in a gene encoding a trehalose-6-phosphate phosphatase (*TPP*), and one in the third *FMO* gene. These nonsynonymous M-linked SNPs represent potential female-sterility mutations. Similarly, we detected 89 nonsynonymous F-specific SNPs across ten genes (Supplementary Data 2). These included one in *TPP*, one in *VviINP1*, seven in an exostosin-coding gene, three in a 3-ketoacyl-acyl carrier protein synthase III gene (*KASIII*), seven in a PLATZ TF-coding gene (*PLATZ*), 18 in the first *FMO* gene, 26 in the second *FMO*, 11 in the third *FMO*, 11 in the hypothetical protein *VviFSEX*, and four in *VviAPT3*. Three of these SNPs introduce a premature stop codon in the first two of four *FMO* genes (Fig. 3e).

To better understand the history of the SDR and to further identify male-sterility and female-sterility candidate genes, we constructed phylogenies from *Vitis* sequences for each SDR gene (Fig. 3f; Supplementary Fig. 4**)**. Alleles tended to cluster by sex type across most of the SDR with the pattern of clustering varying along the locus (Fig. 3f; Supplementary Fig. 4). The phylogenies of four genes at the beginning of the region (from *VviYABBY3* to the aldolase gene) clustered most M sequences apart from F and H sequence, with the *VviYABBY3* and aldolase alleles forming clades that separated M from F and H orthologs (Fig. 3f). This pattern switched from *TPP* onward; for *TPP*, *VviINP1*, *exostosin*, *KASIII*, *PLATZ*, the three *FMO*, the hypothetical protein gene *VviFSEX*, and *VviAPT3*, F sequences clustered apart from M and H alleles (Fig. 3f). These phylogenies are consistent with the observed clusters of sex-specific polymorphisms (Fig. 3a, b), with F-like H haplotypes at the beginning of the region and M-like H haplotypes towards the end of the region (Fig. 2). Genes at the edges of the region do not cluster haplotypes by sex type, further supporting our inference of SDR boundaries (Fig. 3f; Supplementary Fig. 4). Together, these observations are consistent with the emergence of H haplotypes via a recombination event near the aldolase and *TPP* genes, where the pattern of sex-specific clustering shifts (Fig. 3e).

### An INDEL in *VviINP1* is conserved in all female haplotypes

**Fig. 4:**
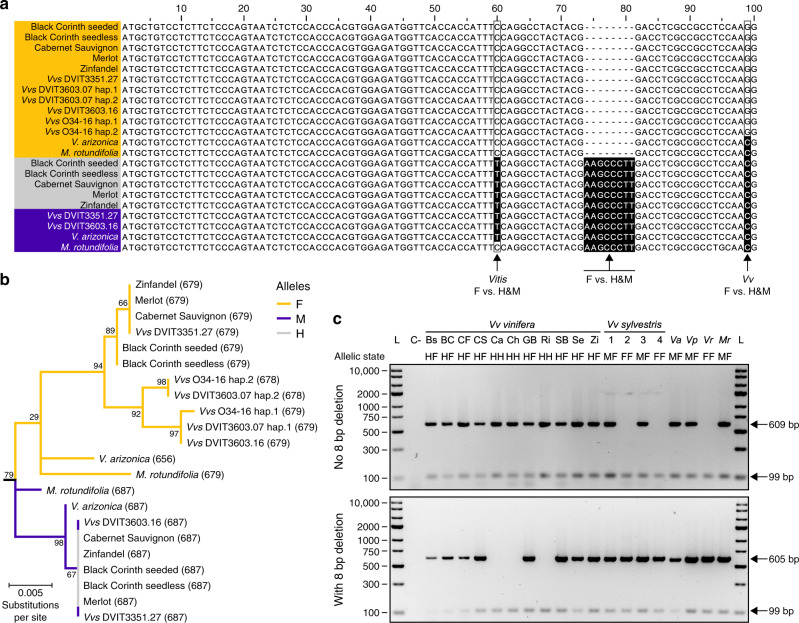
Mutations and segregation in *VviINP1*. **a** Alignment of the first 100 bp of 20 *VviINP1* coding sequences representing 12 F, 5 H and 3 M haplotypes along with two *M. rotundifolia INP1* coding sequences from F and M haplotypes. Alignment revealed an F-linked 8 bp INDEL in *VviINP1* throughout *Vitis* and shared with *M. rotundifolia*. Yellow, purple and gray colors represent F, M and H haplotypes, respectively. **b** Phylogenetic subtree of the *INP1* coding sequences from *Vitis* spp. and *M. rotundifolia*. The tree was rooted with *INP1* sequences from seven outgroups (see “Methods”). Tree branches are colored according to sex-determining region haplotype. Scale bar is in the unit of the number of substitutions per site. Sequence length in bp is indicated in parentheses. **c** Marker assay amplifying *INP1* fragment without (top panel, 609 bp) or with (bottom panel, 605 bp) the 8 bp deletion. Actin was used as a PCR positive control (99 bp fragment). This assay was performed three times. *Vvs* *Vv sylvestris*, L Ladder, C− negative control, *Vv vinifera*: Bs seeded Black Corinth, BC seedlees Black Corinth, CF Cabernet Franc, CS Cabernet Sauvignon, Ca Carménère, Ch Chardonnay, GB Gouais blanc, Ri Riesling, SB Sauvignon blanc, Se Semillon, Zi Zinfandel, *Vv sylvestris*: 1, DVIT3351.27; 2, DVIT3603.07; 3, DVIT3603.16; 4, O34-16; *Va* *V. arizonica*, *Vp* *V. piasezkii,* *Vr* *V. romanetii,* *Mr* *M. rotundifolia*. Source data are provided as a Source Data file.

In addition to SNPs, we identified 156 and 25 small INDELs (≤50 bp) shared by all F and M haplotypes, respectively, relative to the Cabernet Sauvignon H reference. Only two of these INDELs were within exons (Supplementary Data 3): (i) a 21 bp INDEL in the first FMO-coding gene, which introduced a premature stop codon in H and M alleles, and (ii) an 8 bp INDEL in the grape ortholog of the *A. thaliana INP1* (AT4G22600), which caused a frameshift and premature stop codon in all F alleles (Fig. 4a). The 8 bp INDEL in F *VviINP1* alleles leads to the truncation of the protein to 41 amino acids (228 amino acids in H/M haplotypes). Because the same 8 bp deletion was also found in the F haplotype of *M. rotundifolia* (Fig. 4a), this F-specific INDEL likely occurred before *Vitis* and *Muscadinia* diverged and is therefore likely to be widespread in *Vitis*. Phylogenetic analysis of the INP1 protein clustered *Vitis* and *Muscadini*a orthologs by sex, confirming the sex-specificity of *INP1* alleles (Fig. 4b; Supplementary Fig. 5).

The sex-specificity of *INP1* alleles provided an opportunity to estimate the divergence date between M and F haplotypes and hence the potential age of dioecy. We calculated the average synonymous distance (dS) between all 52 pairs of F and M alleles of *INP1* to be 0.0275 substitutions per base (95% confidence interval: 0.0258–0.0292). Assuming a generation time of 3 years and a nucleotide substitution rate of 2.5 × 10^−9^ substitutions per base per year^28^, we infer that M and F alleles diverged ~16.5 million years ago (95% confidence interval: 15.5–17.5), a value within the range of uncertainty of the estimated split time between *Vitis* and *Muscadinia*^29^.

To investigate the sex-specificity of the *VviINP1* INDEL further, PCR primers were designed to amplify INDEL-specific alleles of the gene. Seven additional *Vv vinifera* (HF and HH genotypes) accessions and two more distant central Asian *Vitis* species, *V. piasezkii* (male; MF) and *V. romanetii* (female; FF), were analyzed. The presumably functional *VviINP1* allele was present only in genotypes that contained H and M haplotypes, and the INDEL was present only in genotypes containing an F haplotype (Fig. 4c). The same PCR markers were used to screen two F_1_ populations. Among the 218 individuals of the *Vv vinifera* × *V. arizonica* F1 population, 102 individuals were males, 100 females and 16 did not produce any inflorescences, while the *Vv vinifera* × *Vv sylvestris* F1 population was composed of 92 males, 78 females and 8 plants without inflorescences. In both F1 populations, sex trait segregation followed a 1:1 ratio, as expected from an FF × MF cross (*χ*^2^ = 0.02, d.f. = 1, *P* value = 0.890; *χ*^2^ = 1.15, d.f. = 1, *P* value = 0.283). In both populations, all F1 individuals with female flowers were homozygous for the presumably nonfunctional *VviINP1* allele. All F1 individuals with male flowers carried one functional and one nonfunctional copy of *VviINP1* (Supplementary Figs. 6, 7; Supplementary Data 4−5). The 8 bp deletion in *VviINP1* as well as all other sex-linked polymorphisms in the SDR were confirmed by replicated bulk analysis of 120 individuals of the *Vv vinifera* × *V. arizonica* F1 population genotyped by whole-genome sequencing (Supplementary Fig. 8). Finally, it is worth noting that *VviINP1* corresponds to a peak of linkage disequilibrium (*r*^2^ = 0.77) across 50 *Vv vinifera* (HF) accessions (Fig. 3c), suggesting a suppression of recombination at this locus.

Because a functional copy of *INP1* is necessary for fertile pollen development in *Zea mays*^30^, these results support the hypothesis that the 8 bp deletion causes male sterility in homozygous (FF) *Vitis* females and could be responsible for the absence of colpi in *Vv sylvestris* female pollen grains^16,31^. Together, the sequence, phylogenetic, association, and functional evidences suggest that a recessive allele of *VviINP1* containing an 8 bp deletion interrupts male function, making *VviINP1* a plausible male-sterility candidate.

### Sex-linked genes have distinct expression patterns

In order to assess the potential impact of sex-linked polymorphisms on the regulation of SDR genes, we searched for sex-linked TF-binding sites within 3 kbp regions upstream of transcription start sites (Fig. 3d; Supplementary Fig. 9; Supplementary Data 6). M-linked TF-binding motifs were identified upstream of the genes encoding the PPR-containing protein, VviYABBY3, the aldolase, KASIII, FMOs and VviFSEX. Two of the M-linked TF-binding motifs in the promoter region of *VviYABBY3* were associated with flowering and flower development, including SHORT VEGETATIVE PHASE (SVP), which is involved in the control of flowering time by temperature^32^, and BES1-INTERACTING MYC-LIKE1 (BIM1), a brassinosteroid-signaling component involved in *A. thaliana* male fertility^33^. Similarly, we identified TF-binding motifs unique to F haplotypes upstream of *VviINP1*, *exostosin*, *KASIII*, *PLATZ*, *FMOs*, *VviFSEX*, *WRKY*, and *VviAPT3* (Fig. 3d; Supplementary Data 6). In contrast, all F haplotypes lacked TF-binding sites for bHLH TFs and AGAMOUS-LIKE 3 near the promoter region of *VviAPT3* (Supplementary Fig. 10a).

One gene, *WRKY*, was especially interesting with respect to sex-specific TF-binding sites, because of their potential functional implications. In H and M haplotypes, the *WRKY* promoter region had eleven TF-binding motifs that were absent in all F alleles. These TF-binding sites were associated with TFs that affect flowering time and development in *A. thaliana*^34^. The remarkable diversity of sex-specific TF-binding motifs suggests the potential for complex regulation of SDR genes by TFs that are located outside the SDR and that could be influenced by environmental factors. Moreover, the distribution of TF-binding sites among haplotypes suggest that many SDR genes may be differentially regulated in a sex-specific manner.

To examine sex-specific regulation, we quantified transcript abundance by RNA-sequencing (RNA-seq) of flower buds from hermaphrodite *Vv vinifera* Chardonnay (HH) and male and female *Vv sylvestris* DVIT3351.27 (MF), and O34-16 (FF). Previous studies have analyzed gene expression in the SDR and have compared flowers of different sexes^19^. However, these data were sampled from flowers at early developmental stages and may have missed the late steps of sex determination. Accordingly, we sampled flowers at three developmental stages: (i) during the early development of the reproductive structures, (ii) pre-bloom during pollen maturation, and (iii) at anthesis. Sequencing reads were mapped to both haplotypes of the phased Cabernet Sauvignon genome and expression was compared between individuals at each developmental stage. We focused on genes that showed sex-specific gene expression profiles—e.g., genes that were more highly (or lowly) expressed in the female plant compared to the male plant and to the hermaphrodite.

**Fig. 5:**
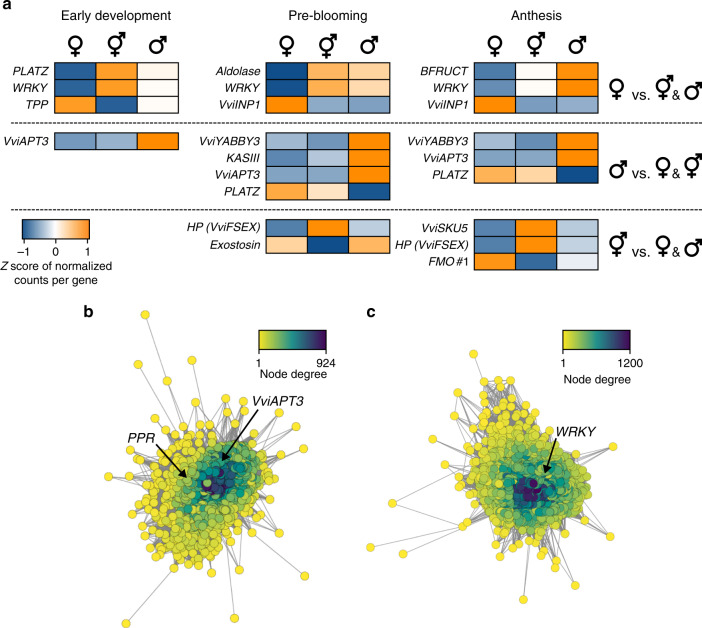
Transcriptome and gene network analyses. **a** Sex-determining region genes have sex-linked expression at each floral stage. Genes are classified in three groups based on their expression pattern. Only genes differentially expressed in one flower sex compared to the two other sex types are shown. The colors of the heat map depict the *Z* score of the normalized counts per gene. Gene coexpression networks of the module magenta (**b**), positively correlated with male sex, and red (**c**), negatively correlated with female sex. Node color indicates the degree of connectivity in the network. Positions of the gene encoding a PPR-containing protein, *VviAPT3* and *WRKY* in their corresponding networks are highlighted. HP hypothetical protein. Source data underlying Fig. 5a are provided as a Source Data file.

Thirteen genes fit this criterion in at least one of the developmental stages (Fig. 5a; Supplementary Data 7). For example—and to our surprise—*VviINP1* was significantly more highly expressed in pre- and post-bloom female flowers compared to male and hermaphroditic flowers (adjusted *P* value ≤ 0.05). Similarly, *WRKY* was more highly expressed in male and hermaphroditic flowers than in female flowers at all developmental stages. Three genes were differentially expressed at only one stage: *TPP, aldolase* and *beta-fructofuranosidase* (*BFRUCT*) (Fig. 5a). Two genes exhibited enhanced expression at two or more stages in male flowers: *VviAPT3* was more highly expressed in males at all three stages and *VviYABBY3* was more highly expressed at the two last developmental stages. The *VviAPT3* results were consistent with a previous study showing that high *VviAPT3* transcript abundance was specific to carpel primordia of *Vv sylvestris* male flowers, suggesting a role in carpel abortion^20^ (Supplementary Fig. 10b). Notably, the high expression of *VviAPT3* in male flowers was specific to the H and M allele and the expression of the F allele was constantly lower across sex types at each developmental stage (Supplementary Fig. 10b, c). This suggests that the expression level or dosage of the *VviAPT3* M and H alleles might influence sex determination. For *VviYABBY3*, we also confirmed that higher expression was specific to the M allele relative to the F allele in male flowers by aligning RNA-seq reads onto the DVIT3351.27 genome (Supplementary Data 8). Given that sequence polymorphisms in *VviYABBY3* are exclusively M-linked (Fig. 3a), and that the gene resides in the portion of the Cabernet Sauvignon H haplotype that resembles an F haplotype (Fig. 2d), it is reasonable to hypothesize that the M allele for *VviYABBY3* could be associated with female sterility.

The differentially expressed SDR genes included TFs and genes associated with hormone signaling that may play significant regulatory roles in coexpression networks^35^. To assess the relationships between sex-linked genes and other genes throughout the genome that may affect development, we performed a Weighted Gene Co-expression Network Analysis across developmental stages (WGCNA^36^) (Supplementary Data 9). Six groups of coexpressed genes (6830 genes in total) were positively or negatively correlated with one of the three sex phenotypes (|Pearson correlation| > 0.9, *P* value < 8e−11; Supplementary Fig. 11). For example, the magenta module of 1176 coexpressed genes (Fig. 5b) was correlated with male sex (Pearson correlation = 0.97; *P* value = 2e−16). This module included two genes in the SDR encoding a PPR-containing protein and *VviAPT3*. The module also included genes involved in hormonal signaling, like two uridine diphosphate glycosyltransferases (UGTs) that are orthologous to *AtUGT85A1* and *AtUGT85A3* and could be involved in active cytokinin homeostasis^37^. *WRKY* expression was central (i.e. highly connected) in the red module (1512 genes; Fig. 5c) that was negatively correlated with female sex (Pearson correlation = −0.92; *P* value = 6e−12). This module included an ortholog of *A. thaliana SEPALLATA1* (*SEP1*; AT5G15800), an MADS-box gene necessary for floral organ development^38^. Altogether, these data suggest that sex-linked polymorphisms affect the regulation of some SDR genes and also that some of these genes are highly connected within coexpression modules that participate in sex determination and other developmental processes.

## Discussion

Dioecy in *Vitis* is interesting because grapevine is one of a few ancestrally dioecious crops that reverted to hermaphroditism during domestication^11^. Structurally, wild *Vitis* spp. have perfect flowers (Fig. 1a, b), but male and female flowers lack functional pistils and pollen, respectively. As is predicted for most angiosperms, these wild *Vitis* flowers are likely derived from hermaphroditic ancestors, with dioecy resulting from independent, sequential male-sterility and female-sterility mutations^3^. Under a two-locus model, the first step to dioecy is the evolution of a recessive male-sterility mutation, and the second step is the formation of a dominant female-sterility mutation^3,39^.

**Fig. 6:**
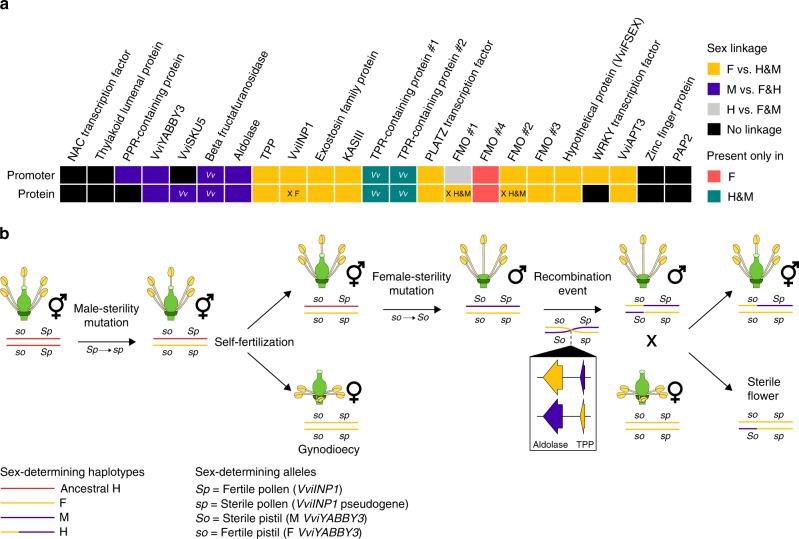
Model of the evolution of sex determination in grapevine. **a** A graphical representation of the association between sex and the observed polymorphisms in promoter regions (top row) and encoded proteins (bottom row) of each gene present in the sex-determining region. Genes affected by nonsense mutations are indicated with an X with the affected haplotype. Sex linkage observed only in *Vitis vinifera* species are indicated with a *Vv*. **b** A potential model for the evolution of dioecy in *Vitis* and its relatives and the reversion to hermaphroditism in cultivated *Vv vinifera*. From left to right, a hermaphroditic ancestor gave rise to male-sterility mutation to produce gynodioecious individuals. Here we denote the recessive male-sterility mutation as *sp*, following the convention of Oberle^39^. Next, male flowers originated as a consequence of a female-sterility mutation(s), labeled *So*, again following the convention of Oberle^39^. According to our data, we hypothesize that a rare recombination event occurred in a *Vv sylvestris* male, leading to H haplotype and hermaphrodite individuals in domesticated *Vv vinifera* cultivars. The symbols ♀, ♂ and  represent female, male and hermaphrodite individuals, respectively.

By mapping the *Vitis* SD haplotypes onto an H reference from Cabernet Sauvignon, we discovered distinct patterns of M-linked and F-linked polymorphisms. M-linked polymorphisms occur in the 5′ region spanning from the promoter of the PPR-containing protein through to the *TPP* gene. In contrast, F-linked polymorphisms span from *TPP* to *VviAPT3* (Fig. 6a). Under the two-locus model of the origin of dioecy, the dominant female-sterility allele is expected to be unique to M haplotypes and therefore located in the region where M haplotypes differ from F and H haplotypes. This narrows a search for the female-sterility locus around the region where M-linked polymorphisms are elevated (Fig. 3a–e). Notably, M-linked SNPs cluster near *VviYABBY3*, and the VviYABBY3 protein sequence clearly differentiates M from F and H haplotypes (Fig. 3f). In addition, *VviYABBY3* contains two M-specific TF-binding sites and exhibits an M-linked gene expression pattern during flower development (Fig. 5a). We therefore hypothesize that one of the key steps in the transition to dioecy was either caused by the amino acid change in the VviYABBY3 protein and/or the upregulation of the *VviYABBY3* gene that caused female sterility in males. Functional information about the YABBY gene family is consistent with this hypothesis. In *A. thaliana*, YABBY genes are involved in floral and lateral organ development^40^, specifically the development of carpels and the ovule outer integument^41^. Though VviYABBY3 is not yet characterized, expression of *V. pseudoreticulata VpYABBY1* and *VpYABBY2* in *A. thaliana* (and of *VvYABBY4* in tomato) implicates these genes in leaf and carpel development^27,42^.

Similarly, F-linked polymorphisms define a region of the SDR that is likely to house the hypothesized recessive male-sterility mutation. F-linked polymorphisms were observed in the latter portion of the SDR from *TPP* to *VviAPT3* (Figs. 3 and 6a). Of the genes in the region, *WRKY* and *VviINP1* are the most noteworthy. The gene *WRKY* is poorly expressed in females relative to males (Fig. 5a) and is part of a coexpression module that is negatively correlated with female sex (Fig. 5c); thus, in theory, it is possible that WRKY expression could contribute to male sterility. However, in our view, the 8 bp deletion in the *VviINP1* gene is the most likely cause of male sterility (Fig. 4a). This homozygous deletion causes a frameshift and premature stop codon in the F allele throughout the genus (Fig. 4c), suggesting it has been maintained throughout *Vitis* history. In *A. thaliana* and maize*, INP1* participates in pollen aperture formation^30,43^; a loss-of-function mutation in grapevine may explain females with sterile inaperturate pollen^31^. All F_1_ males contained at least one functional *VviINP1* (Fig. 4c; Supplementary Figs. 6 and 7), suggesting that if the 8 bp deletion in *VviINP1* causes male sterility it is a recessive allele. Why *VviINP1* was more highly expressed in female flowers is not clear (Fig. 5a), especially given our hypothesis that the deletion event makes the F allele of VviINP1 protein nonfunctional. One possibility is that high expression constitutes a kind of compensatory effect similar to those reported for CRISPR knockouts^44^. Given our hypothesis that the *VviINP1* deletion leads to male sterility, an important future step will be functional confirmation that a homozygous deletion engineered into a hermaphrodite produces female flowers.

Like *VviINP1, VviAPT3* is in the latter portion of the SDR and exhibits F-linked polymorphism, and so its location suggests a possible role in male sterility. However, *VviAPT3* expression was higher in male flowers and its coexpression module correlated with the male sex. These results are consistent with the gene expression pattern described by Coito et al.^20^. These data implicate *VviAPT3* in flower development and sex determination but suggest its mechanism of action is complex and requires further study to understand fully.

It has been hypothesized that recombination between M and F haplotypes caused reversion to H^10,11,45^. Several pieces of evidence support the hypothesis that H haplotypes arose from a recombination event, including their intermediate length and their similarity in structure and sequence to F haplotypes in the first portion of the SDR and to M haplotypes in the latter portion of the SDR (Fig. 6a). Based on our data, we could also localize the hypothetical recombination event. Phylogenetic evidence supports that the recombination event occurred between the *aldolase* and *TPP* genes, and polymorphism information supports that it could have occurred within *TPP* gene, because the gene sequence contained both M- and F-linked nonsynonymous SNPs (Figs. 3a–f, 6b). We note, however, that there is evidence that H haplotypes originated more than once in domesticated grapevine^10,45^, suggesting that there could be different recombination breakpoints across H haplotypes.

Finally, we address one more question about recombination: if recombination can occur between F and M alleles, then what has kept the two haplotypes distinct for so long, given that dioecy has been maintained in the wild since the origin of the genus? This is an especially important question given the hypothesis that the rarity of dioecy among angiosperms is due to easy reversion to hermaphroditism^46^. We speculate that recombination between M and F haplotypes is deterred by at least three features of the *Vitis* SDR. The first is that the close linkage of sex-determining genes may simply reflect physical closeness^39^. If we are correct in our hypotheses that *VviYABBY3* and *VviINP1* are the sterility genes, then recombination events must occur in <100 kbp that separates the two genes to produce an H haplotype. The second is that not all recombination events will be successful in nature: only 50% of correct recombinants will become hermaphrodites (Fig. 6b), and there can be fitness costs associated with hermaphroditism^3^. Finally, we suspect that differences in the structure and length of M and F haplotypes, which are largely attributable to TE accumulation in intergenic space, limit recombination, because recombination can be slowed by differences in TE content between alleles^47^. In this context, the inversion in *M. rotundifolia*, which affects 57% of the M haplotype relative to the F haplotype, may be an especially effective deterrent, because inversions can be barriers to recombination^6,48^. We suspect that these three features contribute to the conspicuous absence of hermaphroditic grapes in the wild.

## Methods

### Plant material

Different plant tissues were collected from several genotypes for genome sequencing, RNA-seq and marker assay. All plant material was immediately frozen and ground to powder in liquid nitrogen after collection. For genome sequencing, young leaves were collected from three hermaphrodite *Vv vinifera* (Merlot clone FPS 15; Black Corinth with parthenocarpic fruit FPS 02.1; Black Corinth with seeded fruit; Supplementary Fig. 12), four dioecious *Vv sylvestris* (male DVIT3351.27 collected from Armenia; female O34-16 collected from Iran; female DVIT3603.07 and male DVIT3603.16 collected from Azerbaijan), one dioecious male *V. arizonica* (b40-14), and one dioecious male *M. rotundifolia* (Trayshed). For RNA-seq, inflorescences were collected in April and May 2019 from vines at the University of California Davis (Davis, CA, USA). These times correspond to two developmental stages^49^: flowers pressed together (E-L 15) and full flowering with 50% caps off (E-L 23; Supplementary Fig. 13). Three genotypes were sampled, including *Vv vinifera* cv. Chardonnay clone FPS 04, male *Vv sylvestris* DVIT3351.27, and female O34-16. Floral buds were sampled from three inflorescences at E-L 15 (time 1). At E-L 23, pre- (time 2) and post-bloom (time 3) flowers were collected separately, each represented by three biological replicates. Young leaves from additional genotypes were collected for a marker assay. This included seven *Vv vinifera*, *V. piasezkii* and *V. romanetii* and two F_1_ populations (Supplementary Table 1). One F_1_ population was the result of a cross between the pistillate *Vv vinifera* F2-35 (Carignane × Cabernet Sauvignon) and *V. arizonica* b42-26. The other F_1_ population was produced by crossing female *Vv vinifera* 08326-61 (selfing of Cabernet Franc) and *Vv sylvestris* DVIT3351.27. The sexes of F_1_ individuals were evaluated by flower morphology and the presence of fruit.

### Library preparation and sequencing

High molecular weight genomic DNA (gDNA) was isolated as in Chin et al.^24^ from each of the nine previously described samples. DNA purity, quantity, and integrity were evaluated with a Nanodrop 2000 spectrophotometer (Thermo Scientific, IL, USA), Qubit 2.0 Fluorometer together with the DNA High Sensitivity kit (Life Technologies, CA, USA), and by pulsed-field gel electrophoresis, respectively. SMRTbell libraries were prepared as described in Minio et al.^50^ and sequenced on a PacBio Sequel system using V3 chemistry, and on a PacBio RS II (Pacific Biosciences, CA, USA) using P6-C4 chemistry for *Vv vinifera* cv. Merlot (DNA Technology Core Facility, University of California, Davis). Summary statistics of SMRT sequencing are provided in Supplementary Data 1. DNA extractions from the two F_1_ populations were performed using a modified CTAB protocol^17^. DNA concentration was measured using Qubit 2.0 Fluorometer. For the bulk segregant analysis, 120 DNA samples from the F_1_ population *Vv vinifera* F2-35 × *V. arizonica* b42-26, 60 females and 60 males, were selected to constitute four pools of 30 individuals each, two made of DNA from male individuals and two made of DNA from female individuals. A total of 1 μg DNA per sample was used as the input material. DNA-seq libraries were prepared using the Kapa LTP library prep kit (Kapa Biosystems, MA, USA). Libraries were evaluated for quantity and quality with the High Sensitivity chip on a Bioanalyzer 2100 (Agilent Technologies, CA, USA) and sequenced in paired-end 150 bp reads on an Illumina HiSeq4000. Total RNA were extracted from floral buds as described in Rapicavoli et al.^51^ from each of the three genotypes described above. cDNA libraries were prepared using the Illumina TruSeq RNA sample preparation kit v.2 (Illumina, CA, USA) and sequenced in single-end 100 bp reads on an Illumina HiSeq4000 (Supplementary Data 10). Four samples from the male genotype (three replicates at time 2 and one at time 3) were re-sequenced in paired-end 150 bp reads to improve library quality and depth.

### Genome assembly and annotation

Genome assembly of hermaphrodite *Vv vinifera* cv. Merlot was performed at DNAnexus (Mountain View, CA, USA) (Supplementary Method 1). For each of the other eight genotypes, SMRT reads were assembled with a custom FALCON-Unzip pipeline^52^ reported in https://github.com/andreaminio/FalconUnzip-DClab. Repetitive regions were marked using the DAmasker TANmask and REPmask modules^53^ before and after SMRT read error correction. Then, error-corrected reads were assembled with FALCON v.2017.06.28-18.01^24^. This included setting different minimum seed-read lengths (length_cutoff_pr parameter) to improve the contiguity of the primary assembly (Supplementary Data 1). Haplotype phasing was carried out using FALCON-Unzip. FALCON-Unzip was designed to combine single-nucleotide polymorphisms and structural variants to separate long sequencing reads based on their haplotype, which are then assembled into separate contigs^24^. FALCON-Unzip was shown to successfully phase heterozygous regions in plants, including grapes^24,52^. FALCON-Unzip was performed with default settings. Primary contigs and haplotigs were both polished with Arrow from ConsensusCore2 v.3.0.0. To further improve sequence contiguity, primary contigs of Cabernet Sauvignon^24^ and the other nine assemblies were scaffolded using SSPACE-Longread v.1.1^54^ and gaps were closed with PBJelly from PBsuite v.15.8.4^55^. Summary statistics of the ten genome assemblies is provided in Supplementary Data 1. Additional scaffolding and gap-closing steps were performed on the Cabernet Sauvignon genome assembly to construct pseudomolecules (see details in Supplementary Method 2). For all new nine assemblies, genome annotation was performed as described previously for *Vv vinifera* cv. Zinfandel^26^. Details are provided in Supplementary Method 3.

### SDR localization and haplotype reconstruction

The grape SDR was identified by aligning the SSR marker VVIB23^17^ and genes previously associated with the SDR^10^ (Supplementary Table 2) to the chromosome-scaled Cabernet Sauvignon genome assembly. Protein-coding sequences (CDS) of Cabernet Sauvignon hap1 in this genomic region were then aligned to the ten other genome assemblies with GMAP v.2015-09-29^56^ to identify homologous regions. When the alignments of SDR-associated sequences were fragmented (i.e. with genes aligned to multiple contigs), BLAT v.36x2^57^ was used to determine the overlap between sequences and contigs were manually joined. Junction gaps of ten bases were added between overlaps. A schematic representation of the haplotypes was made using the Gviz Bioconductor package v.1.20.0^58^.

### Transcription factor-binding site analysis

For each haplotype, promoter sequences were extracted for all the genes of the SDR using the R package GenomicFeatures v.1.36.4^59^ with a maximum of 3 kbp upstream regions from the gene transcriptional start sites. TF-binding sites were identified using the R package TFBSTools v.1.22.0^60^ and the JASPAR database (R package JASPAR2018 v.1.1.1^61^).

### Linkage disequilibrium analysis

Illumina whole-genome resequencing data from 50 *vinifera* accessions from previous studies^22,28^ (mean depth = 21.6×) were trimmed using Trimmomatic v.0.36^62^ to remove adaptor sequences and bases for which average quality per base dropped below 20 in 4 bp window. Trimmed paired-end reads were aligned onto Cabernet Sauvignon primary pseudomolecules (hap1) using Minimap2 v.2.17^63^. Alignments with a mapping quality <10 were removed using Samtools v.1.9^64^. PCR duplicates introduced during library preparation were filtered in MarkDuplicates in the picard-tools v.1.119 (https://github.com/broadinstitute/picard). INDEL realignment was performed using RealignerTargetCreator and IndelRealigner (GATK v.3.5^65^). Sequence variants were called using HaplotypeCaller (GATK v.4.1.2.0^65^). SNPs were filtered using VariationFiltration (GATK v.4.1.2.0^65^), according to the following criteria: variant quality (QD) > 2.0, quality score (QUAL) > 40.0, mapping quality (MQ) > 30.0, and <80% missing genotypes across all samples. Linkage disequilibrium measured as the correlation coefficient of the frequencies (*r*^2^) were calculated using Plink v.2.0^66^ for pairwise SNPs with a minor allele frequency (MAF) > 0.05 across the whole SDR (chr2:4750000..5100000).

### Whole-sequence alignments and structural variation analysis

Pairwise alignments of all the haplotypes were performed using NUCmer from MUMmer v.4.0.0^67^ and the --mum option using Cabernet Sauvignon hap1 as a reference. Structural variants (SVs; >50 bp) including deletions, insertions, duplications, inversions, translocations, and complex insertion-deletions (CIDs) and short INDELs (<50 bp) were called using show-diff and show-snps, respectively. SNPs were called using show-snps from MUMmer v.4.0.0 and the -1 filter. Comparison between haplotypes for SVs and SNPs was performed using multiinter from BEDTools v.2.19.1^68^. Polymorphisms were considered as fully sex-linked only if they were strictly fixed in one sex haplotype compared to the other two sex haplotypes. SNPs and INDELs were confirmed by manually inspecting the alignments of each genotype whole-genome short- and long-read sequences onto their corresponding genomes. Alignments were visualized using Integrative Genomics Viewer (IGV) v.2.4.14 and phasing the two haplotypes^69^.

### Phylogenetic analysis

Phylogenetic analysis of the 11 genomes was based on orthology inference (see Supplementary Method 4). Phylogenetic analysis of the proteins and promoter regions of genes in the SDR were conducted with MEGA7^70^ using the Neighbor-Joining method^71^ and 1000 replicates. Evolutionary distances were computed using the Poisson correction method^72^ and are expressed as the number of amino acids or base substitutions per site. All positions with less than 5% site coverage were eliminated. Phylogenetic analysis of the *INP1* coding sequences from *Vitis* spp. and *M. rotundifolia* was performed with seven outgroups: three Brassicaceae: *Matthiola incana*, *A. thaliana*, *Capsella rubella*, *Solanum lycopersicum* (Solanaceae), *Eschscholzia californica* (Papaveraceae), and two Poaceae, *Zea mays* and *Brachypodium distachyon*^30^ (Supplementary Fig. 5), using the Maximum Likelihood method based on the Tamura-Nei model^73^ and 1000 iterations in MEGA7^70^.

All 52 possible pair of sequences were constructed between the F and M alleles of *INP1* coding sequences in *Vitis* and *M. rotundifolia*, based on an alignment of 687 nucleotides. The average synonymous distance (dS) was computed for each sequence pair using the yn00 program in the PAML package v.4.9^74^. The resulting distribution of dS values was not significantly different from a normal distribution using a Shapiro test (*W* = 0.95765, *P* value = 0.06189) and hence normality was assumed to construct 95% confidence intervals.

### Transcript abundance quantification

Illumina reads were trimmed using Trimmomatic v.0.36^62^ and the following settings: LEADING:3 TRAILING:3 SLIDINGWINDOW:10:20 CROP:100 MINLEN:36. For the four samples with pair-ended reads, only the first mate was used and samples were randomly down-sampled to 26 million reads with seqtk v.1.0-r57-dirty (https://github.com/lh3/seqtk) before trimming. These reads were then mapped onto Cabernet Sauvignon phased genome and *Vv sylvestris* DVIT3351.27 genome using HISAT2 v.2.0.5^75^ with the following settings: --end-to-end --sensitive --no-unal -q -t --nondeterministic -k 1 (Supplementary Data 10). The Bioconductor package GenomicAlignments v.1.12.2^59^ was used to count reads per gene locus. Read-count normalization and statistical testing of differential expression were performed using DESeq2 v.1.16.1^76^. Coexpression analysis was conducted with WGCNA v.1.66^36^ using log_2_-transformed normalized counts (log_2_(normalized counts +1)). A soft-thresholding power of 12 with a scale-free model fitting index *R*^2^ > 0.92 was applied with a minimum module size of 30. The weighted network was converted into an unweighted network preserving all connections with topological overlap mapping metric (TOM) > 0.02, and imported into Cytoscape v.3.7.2^77^.

### Marker assay

Three primers (two forward, one reverse) were designed to determine the presence or absence of an 8 bp deletion in *VviINP1*: INP1_HM-F, 5′-CCTACTACGAAGCCCTTGACC-3′; INP1_F-F, 5′-CAGGCCTACTACGGACCTC-3′; INP1-R, 5′-CCGTGCAGCGTTCAAATTCACTG-3′. *Actin*-specific primers^78^ were used to provide a positive amplification control. The PCR conditions included a 3 min denaturation at 95 °C, followed by 35 cycles of 35 s at 95 °C, 30 s at 60 °C and 40 s at 72 °C, and a final extension for 5 min at 72 °C.

### Bulk segregant analysis

Illumina reads from the four pools (mean depth = 172×) were trimmed using Trimmomatic v.0.36^62^ and the following settings: LEADING:7 TRAILING:7 SLIDINGWINDOW:10:20 MINLEN:36. Trimmed paired-end reads were aligned onto Cabernet Sauvignon hap1 genome using BWA v.0.7.17-r1188^79^ with default parameters. PCR and optical duplicates were removed with picard-tools v.2.0.1 (http://broadinstitute.github.io/picard/). Sequence variants were called using UnifiedGenotyper from GATK v.3.5-0-g36282e4^65^. Variants were filtered according to the following criteria: read depth (DP) > 5 and quality score (QUAL) > 40.

### Reporting summary

Further information on research design is available in the Nature Research Reporting Summary linked to this article.

## Supplementary information

Supplementary Information

Peer Review File

Reporting Summary

Description of Additional Supplementary Files

Supplementary Data 1

Supplementary Data 2

Supplementary Data 3

Supplementary Data 4

Supplementary Data 5

Supplementary Data 6

Supplementary Data 7

Supplementary Data 8

Supplementary Data 9

Supplementary Data 10

## Data Availability

Data supporting the findings of this work are available within the paper and its Supplementary Information files. A reporting summary for this Article is available as a Supplementary Information file. The datasets generated and analyzed during the current study are available from the corresponding authors upon request. Sequencing data are accessible through NCBI under the BioProject ID PRJNA593045. Genome sequences and gene annotation files are available at 10.5281/zenodo.3827985. The source data underlying Figs. 2, 3a−d, f, 4a, b, and 5a, as well as Supplementary Figs. 1–5 and 8–10 are provided as a Source Data file. Source data are provided with this paper.

## Source data

Source Data

## Code availability

The script HaploSync is available at Github (https://github.com/andreaminio/HaploSync). Source data are provided with this paper.
