# Supplementary material for: The genetic basis of sex determination in grapes
Source: Nat Commun. 2020 Jun 9;11:2902. doi: 10.1038/s41467-020-16700-z (PMC7283251; doi:10.1038/s41467-020-16700-z)
Supplement: Supplementary file 1 — Supplementary Information [file 41467_2020_16700_MOESM1_ESM.pdf]

The genetic basis of sex determination in grapes

Massonnet *et al.*

Supplementary Figures

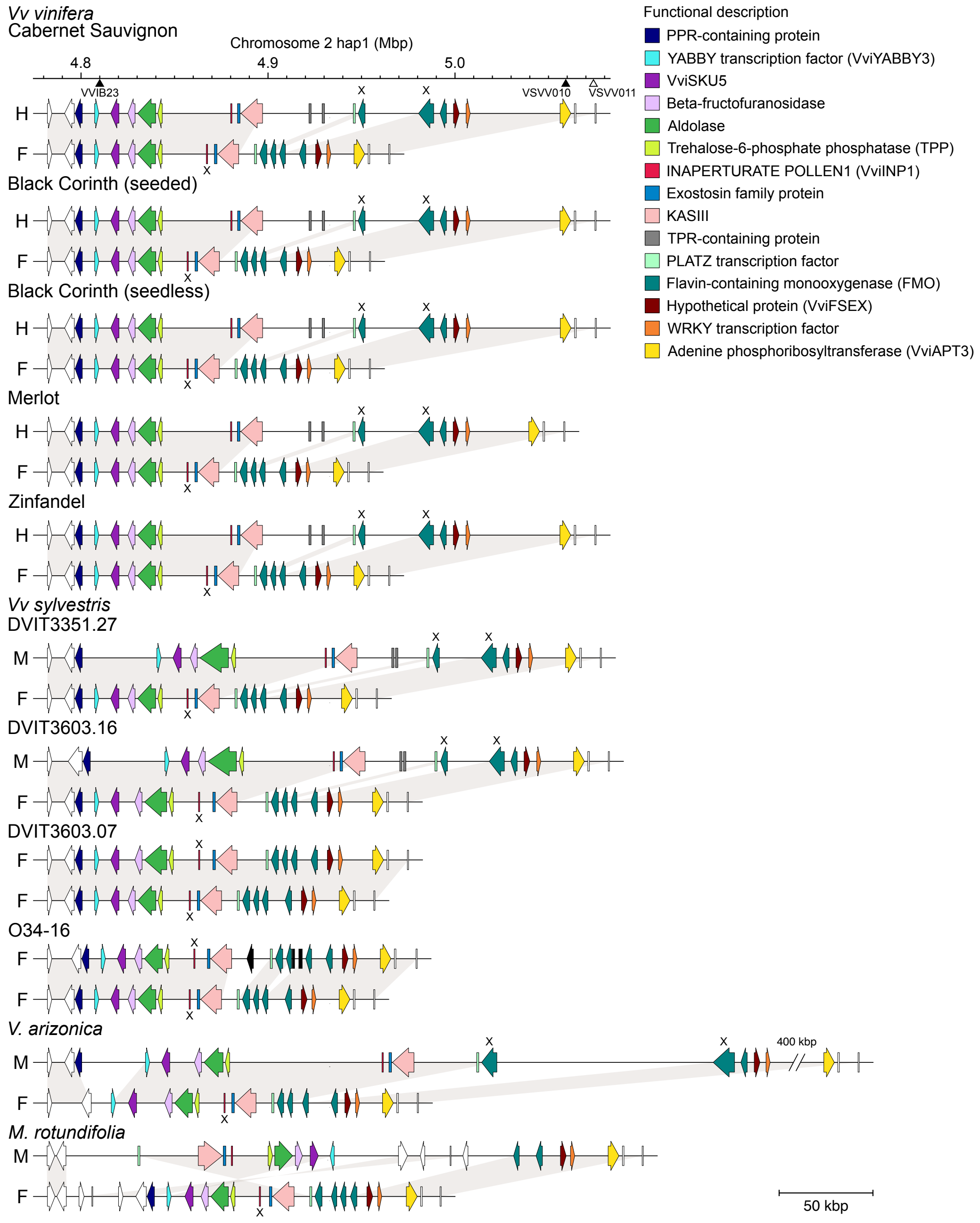

Supplementary Fig. 1: Gene content is highly conserved in the grape sex-determining region. Schematic representations of the sex-determining region haplotypes. Black-filled triangles along chromosome 2 mark the position of the genetic marker VVIB23 and the amplicon VSVV010, both of which are closely linked to the sex-determining locus[1,2], while no linkage to sex was detected in the amplicon VSVV011[2] (white-filled triangle). Genes affected by nonsense mutations are indicated with an X. Black- and white-colored arrows depict genes that are present only in one haplotype and genes that do not show any sex linkage, respectively. Source data are provided as a Source Data file.

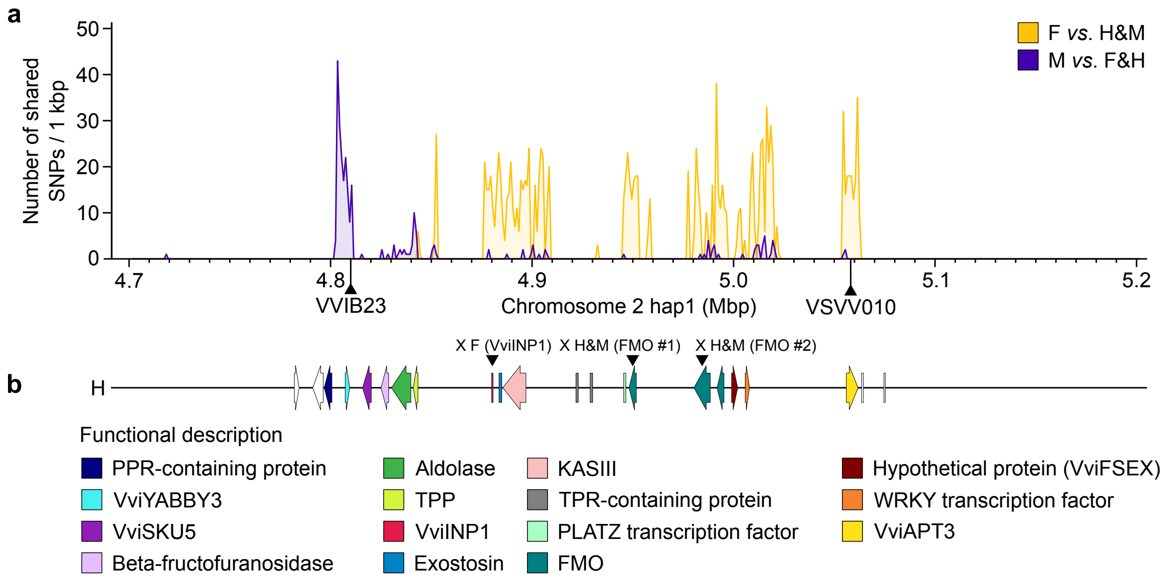

Supplementary Fig. 2: Sex-linked SNPs on Cabernet Sauvignon chromosome 2 hap1. **a**, Number of shared sex-linked SNPs per kbp from the position 4.7 to 5.2 Mbp. **b**, Gene composition of the H haplotype of *Vv vinifera* Cabernet Sauvignon hap1. Two triangles along chromosome 2 mark the position of the genetic marker VVIB23 and the amplicon VSVV010, both closely linked to the sex-determining region[1,2]. Source data are provided as a Source Data file.

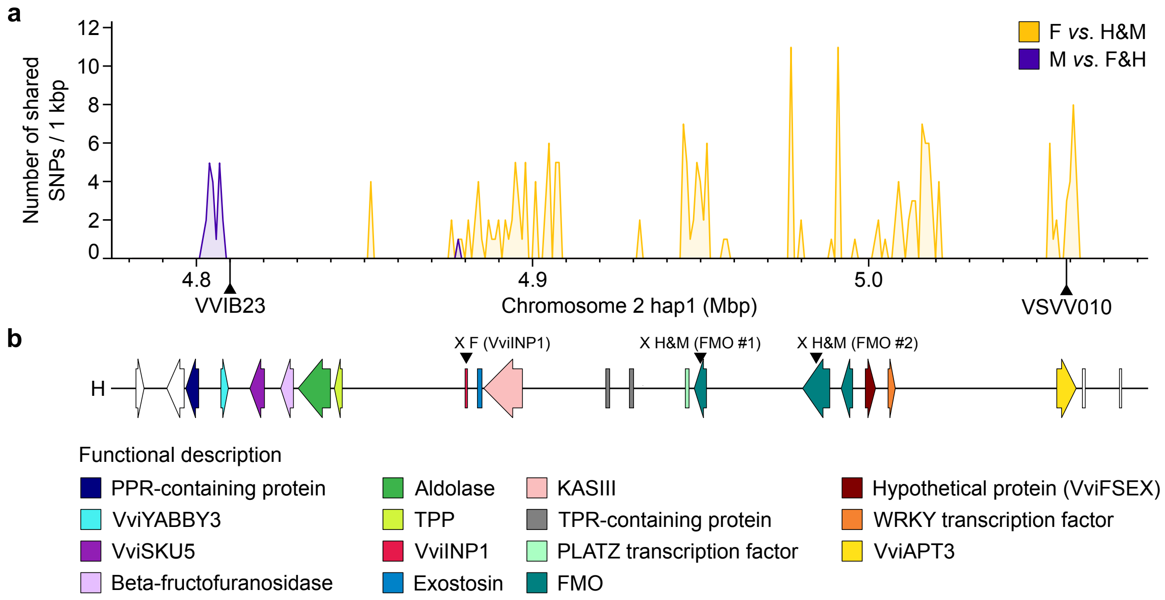

Supplementary Fig. 3: Sex-linked SNPs along the sex-determining region with *M. rotundifolia*. **a**, Number of shared sex-linked SNPs per kbp. **b**, Gene composition of the H haplotype of *Vv vinifera* Cabernet Sauvignon hap1. Two triangles along chromosome 2 mark the position of the genetic marker VVIB23 and the amplicon VSVV010, both closely linked to the sex-determining locus[1,2]. Source data are provided as a Source Data file.

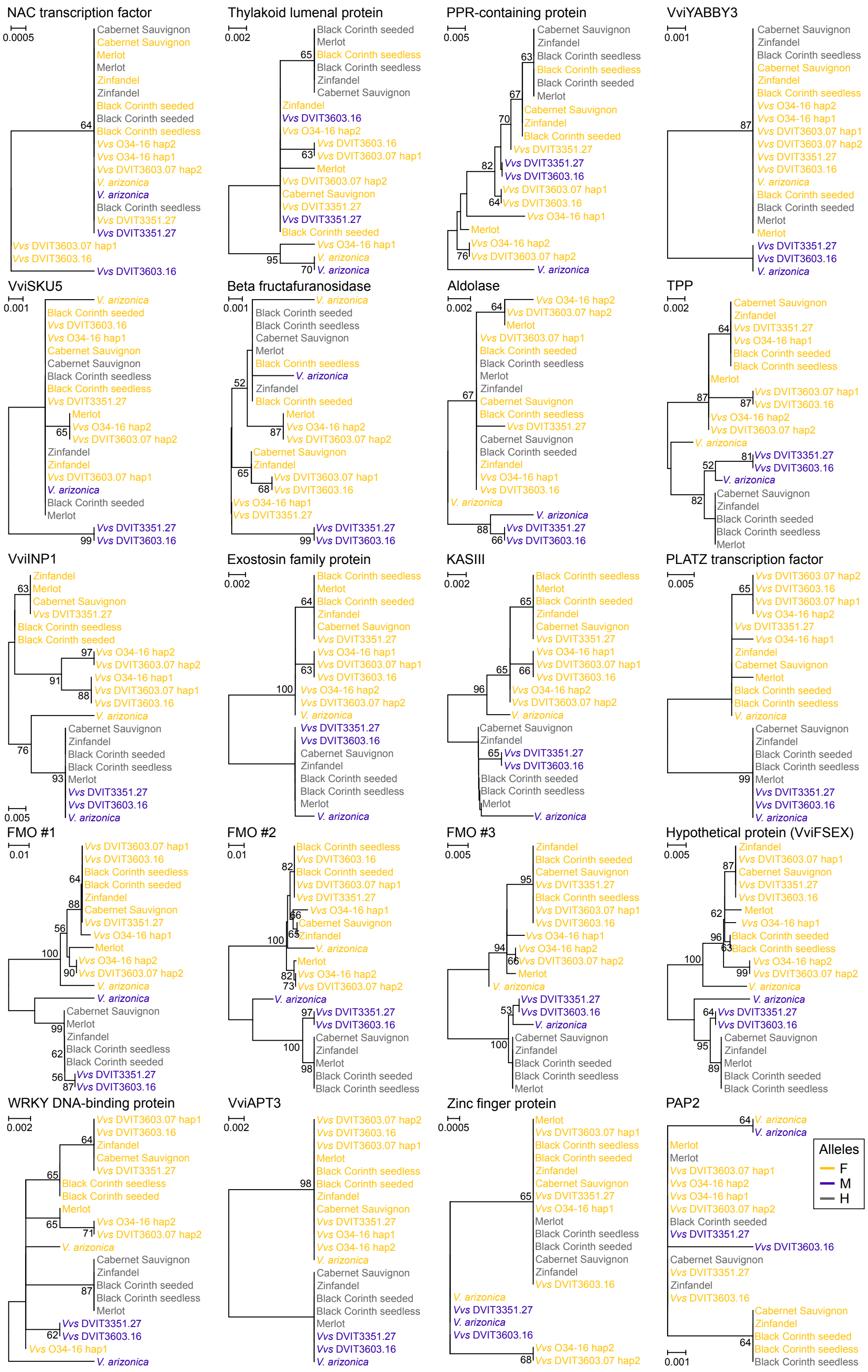

Supplementary Fig. 4: Sex-linked polymorphisms affect protein sequences. Neighbor-joining clustering of the protein sequences encoded by each gene of the sex-determining locus. Scale bars are in the unit of the number of substitutions per site. Source data are provided as a Source Data file.

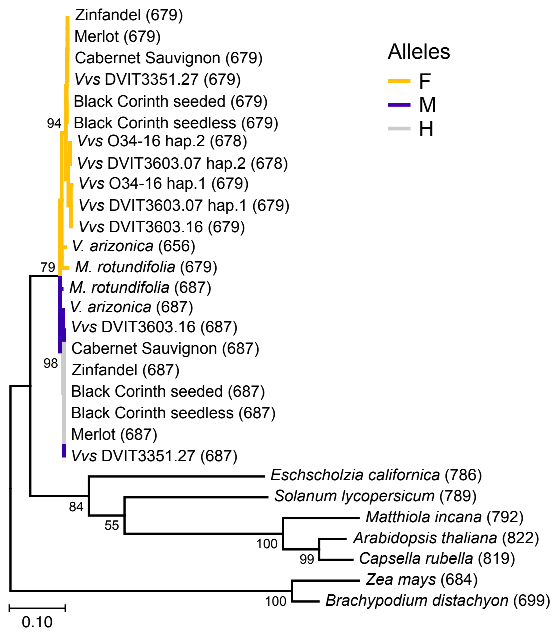

Supplementary Fig. 5: Maximum-likelihood clustering of INP1 coding sequences from *Vitis* spp., *M. rotundifolia* and seven outgroups.

Phylogenetic analysis was performed with INP1 coding sequences from *Vitis* spp., *M. rotundifolia* seven outgroups: three Brassicaceae, *Matthiola incana*, *Arabidopsis thaliana*, *Capsella rubella*, one Solanaceae, *Solanum lycopersicum*, one Papaveraceae, *Eschscholzia californica*, and two Poaceae, *Zea mays* and *Brachypodium distachyon*[3]. Tree branches are colored according to sex-determining locus haplotype. Scale bar is in the unit of the number of substitutions per site. Sequence length in bp is indicated in parentheses. Source data are provided as a Source Data file.

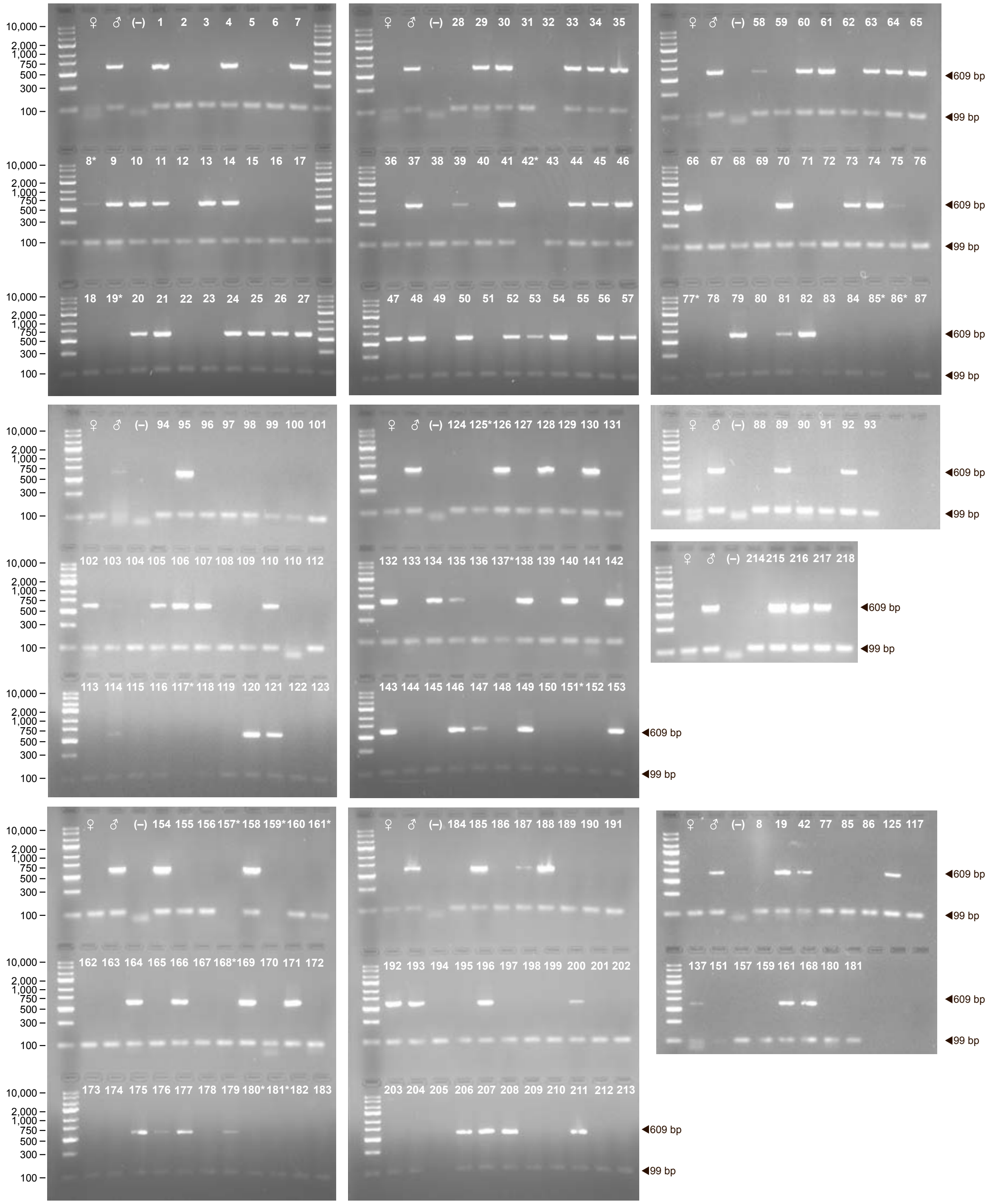

Supplementary Fig. 6: Marker assay in the population *Vv vinifera* F2-35 x *V. arizonica* b42-26.

Marker assay amplifying M *VviINP1* fragment, *i.e.* without the 8 bp deletion (609 bp). Actin was used as a PCR positive control (99 bp fragment). The female *Vv vinifera* F2-35 and the male *V. arizonica* b42-26 are indicated by the symbols ♀ and ♂, respectively. This assay was performed once. Samples with (*) were repeated. Results are summarized in **Supplementary Data 4**.

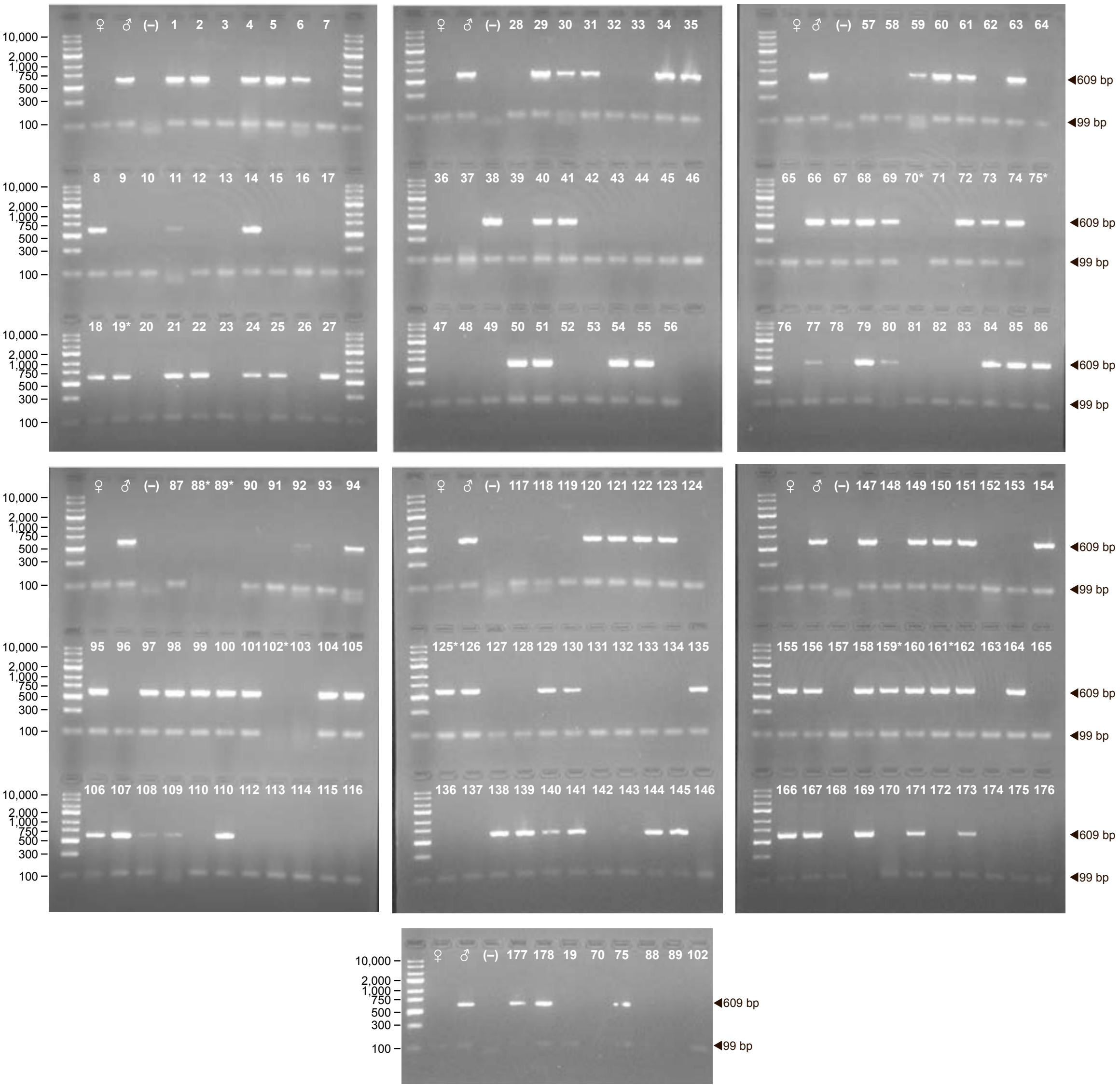

Supplementary Fig. 7: Marker assay in the population *Vv vinifera* 08326-61 x *Vv sylvestris* DVIT3351.27.

Marker assay amplifying M *VviINP1* fragment, *i.e.* without the 8 bp deletion (609 bp). Actin was used as a PCR positive control (99 bp fragment). The female *Vv vinifera* 08326-61 and *Vv sylvestris* DVIT3351.27 are indicated by the symbols ♀ and ♂, respectively. This assay was carried out once. Samples with (*) were repeated. Results are summarized in **Supplementary Data 5**.

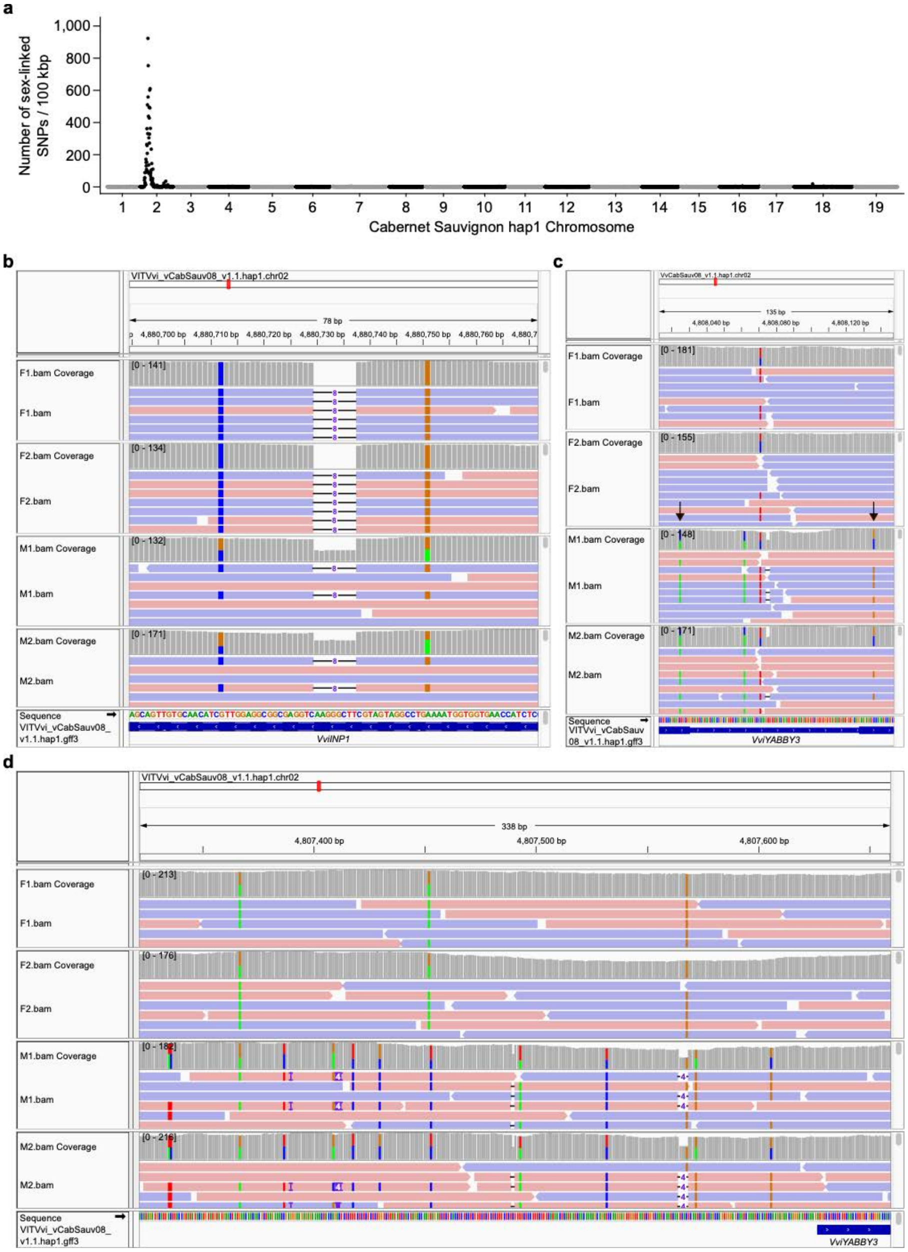

Supplementary Fig. 8: Bulk segregant analysis on 120 individuals from the population *Vv vinifera* F2-35 x *V. arizonica* b42-26.

a, The number of sex-linked SNPs per 100 kbp across Cabernet Sauvignon hap1 genome. SNPs were identified by aligning short-read sequencing data from 120 individuals of the F1 population *Vv vinifera* F2-35 x *V. arizonica* b42-26 split in four bulks, two composed of female individuals (FF), called F1 and F2, two made of male individuals (MF), called M1 and M2. SNPs in homozygous state in both female pools (0/0/0/0 or 1/1/1/1) and in heterozygous state in both male pools (0/0/1/1) were considered fully sex-linked. Bulk segregant analysis confirmed the location of the sex-determining region on chromosome 2. Alignment of the short-read sequencing data confirm complete sex linkage of the 8 bp deletion in *VviINP1* (**b**), as well as the two non-synonymous SNPs in *VviYABBY3* (**c**) and SNPs identified in its promoter region (**d**). Source data are provided as a Source Data file.

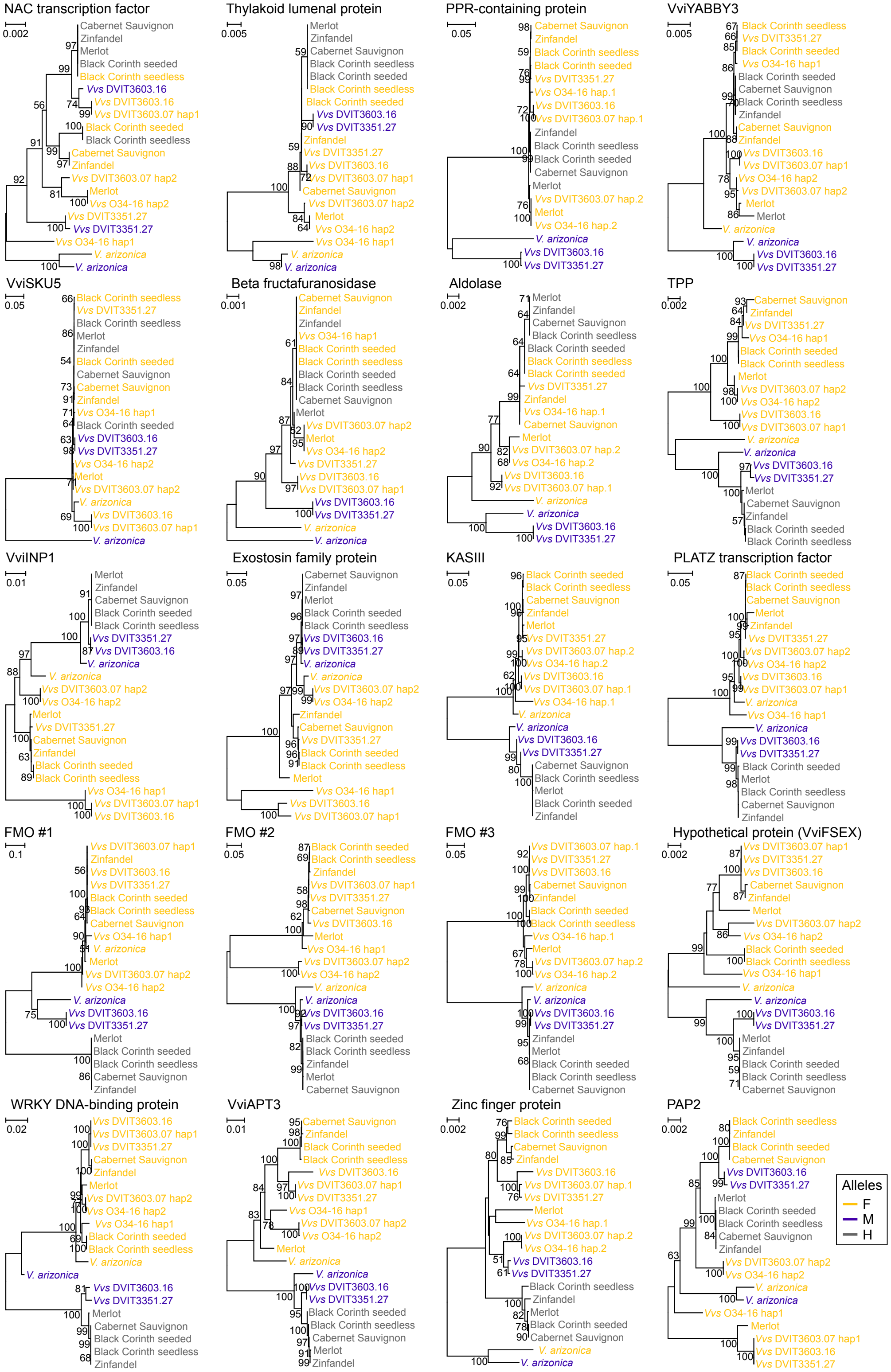

Supplementary Fig. 9: Sex-linked polymorphisms affect promoter region. Neighbor-joining clustering of the 3 kbp region upstream of transcription start site of each gene of the sex-determining locus. Scale bars are in the unit of the number of substitutions per site. Source data are provided as a Source Data file.

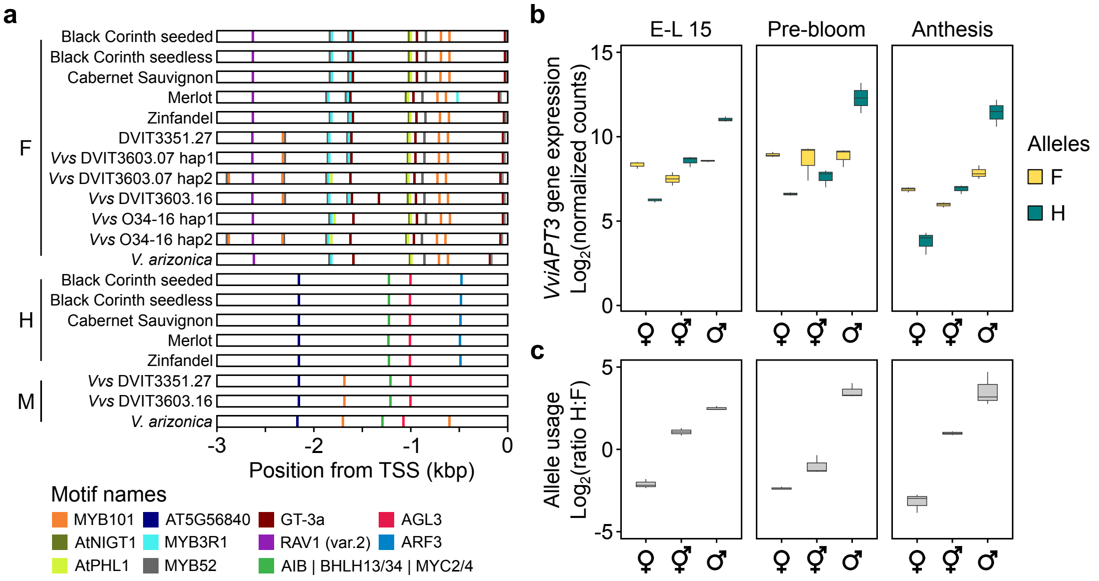

Supplementary Fig. 10: *VviAPT3*, sex-linked factor-binding sites, gene expression and allele usage.

a, Sex-linked transcription factor-binding sites found within 3 kbp region upstream of *VviAPT3* transcription start site (TSS). **b**, *VviAPT3* gene expression during floral development in three sex types. Mapping of the RNA-seq reads was performed on the entire genome of Cabernet Sauvignon in a non-deterministic way. For each gene of the sex-determining locus, read counting was done by allele, *i.e.* H and F alleles separately. In absence of sequence variation, RNA-seq reads were assigned evenly to F and H haplotypes. **c**, *VviAPT3* allele usage by flower sex at each developmental stage. For box plots, the middle bar represents the median, the bottom and top of each box represent the 25th and 75th percentiles, respectively, and the whiskers extend to 1.5 times the interquartile range (n = 3 biologically independent samples per each sex type). Abbreviations: F, female; H, hermaphrodite; M, male. Source data are provided as a Source Data file.

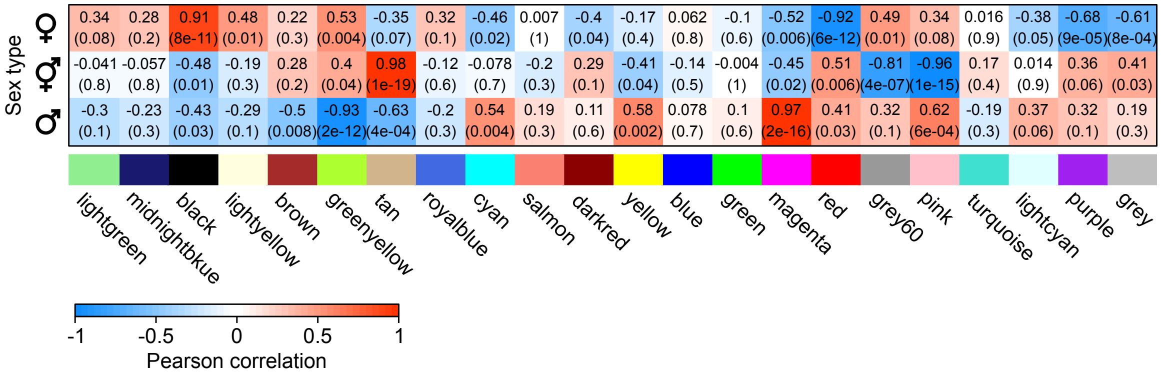

Supplementary Fig. 11: Sex trait-module association heatmap.
The rows correspond to the flower sexes and the columns to module eigengenes (WGCNA). The cell labels denote weighted Pearson correlation (supported through the color legend) and the Student asymptotic P value of the corresponding module and group (n = 9 for each sex type).

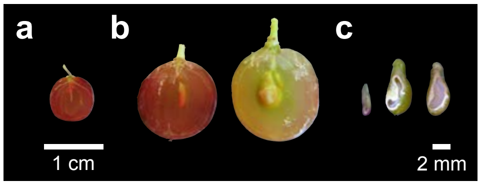

Supplementary Fig. 12: Seedless and seeded Black Corinth berries and seeds.
Seedless Black Corinth fruit is parthenocarpic (**a**) whereas the seeded Black Corinth berries (**b**) exhibit aborted (left) and fully-developed (right) seeds. **c**, Three types of seeds were observed in the seeded Black Corinth: aborted seed (left), empty seed (middle), and filled seed (right).

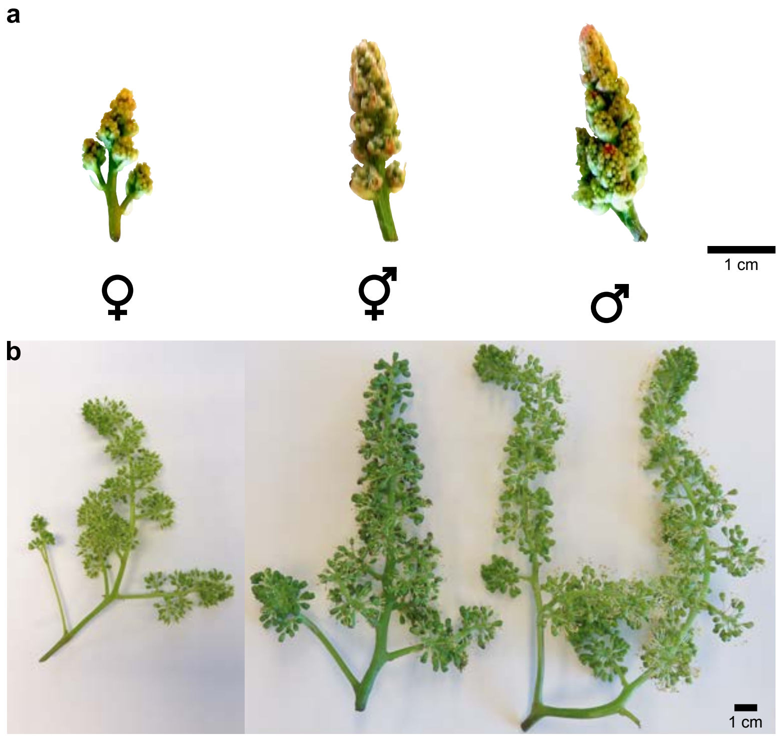

Supplementary Fig. 13: Inflorescences at E-L 15 and E-L 23 developmental stages. For the RNA sequencing assay, inflorescences of *Vv sylvestris* O34-16 (female), DVIT3351.27 (male) and *Vv vinifera* cv. Chardonnay were collected at the developmental stage E-L 15 (**a**), flowers pressed together, E-L 23 (**b**), full flowering with 50% caps off.

Supplementary Tables

Supplementary Table 1: Information about the genotypes used for marker assay presented on Fig. 4c.

**Genotype**	**Genus**	**Species**	**Subspecies**	**Accession number**	**Flower sex**	**Allelic State**
Black Corinth seeded	*Vitis*	*vinifera*	*vinifera*	Collected by R.J. Weaver in Visalia, CA, USA	Hermaphrodite	HF
Black Corinth seedless	*Vitis*	*vinifera*	*vinifera*	clone FPS 02.1	Hermaphrodite	HF
Cabernet Franc	*Vitis*	*vinifera*	*vinifera*	clone FPS 04	Hermaphrodite	HF
Cabernet Sauvignon	*Vitis*	*vinifera*	*vinifera*	clone FPS 08	Hermaphrodite	HF
Carmenere	*Vitis*	*vinifera*	*vinifera*	clone FPS 02	Hermaphrodite	HH
Chardonnay	*Vitis*	*vinifera*	*vinifera*	clone FPS 04	Hermaphrodite	HH
Gouais blanc	*Vitis*	*vinifera*	*vinifera*	clone FPS 01	Hermaphrodite	HF
Riesling	*Vitis*	*vinifera*	*vinifera*	clone FPS 24	Hermaphrodite	HH
Sauvignon blanc	*Vitis*	*vinifera*	*vinifera*	clone FPS 01	Hermaphrodite	HF
Semillon	*Vitis*	*vinifera*	*vinifera*	clone FPS 03	Hermaphrodite	HF
Zinfandel	*Vitis*	*vinifera*	*vinifera*	clone FPS 03	Hermaphrodite	HF
DVIT3351.27	*Vitis*	*vinifera*	*sylvestris*	DVIT3351.27	Male	MF
DVIT3603.07	*Vitis*	*vinifera*	*sylvestris*	DVIT3603.07	Female	FF
DVIT3603.16	*Vitis*	*vinifera*	*sylvestris*	DVIT3603.16	Male	MF
O34-16	*Vitis*	*vinifera*	*sylvestris*	DVIT1803	Female	FF
Vitis arizonica	*Vitis*	*arizonica*		b40-14	Male	MF
Vitis piasezkii	*Vitis*	*piasezkii*		DVIT2027	Male	MF
Vitis romanetii	*Vitis*	*romanetii*		DVIT2732	Female	FF
Muscadinia rotundifolia Trayshed	*Muscadinia*	*rotundifolia*		Trayshed	Male	MF

Supplementary Table 2: Primers used to localize the grape sex-determining locus.

Name	Forward	Reverse	Reference
VVIB23	GGTCACGTAGATATTGAAGTTG	TTTGTATTTTGGGCATTTGCAG	[1]
VSVV006	TGGATTAGCTTGGCGAATAAA	CACGGTCAGGATCATCAACA	[2]
VSVV007	CTCTCCGTTTTCCATTTCCA	TCCTCAACCCCAGTGAACTC	[2]
VSVV008	AAGGATACACGCCCTCACAG	AGCAATCAGCAGCCAAATCT	[2]
VSVV009	AGGAAACTGAGGATAAAGAGATCGT	GGATAGACAGCGAGTTAGCACTTAG	[2]
VSVV010	AGTGCTCACTTTTCCTTGTGAA	CATGAATCAGCAGTGCATTT	[2]
VSVV011	AGCAATGGGAGGGTCTTTTT	TAAAATCAACCGCCTTACCG	[2]

Supplementary Methods

Supplementary Method 1

Genome assembly of *Vv vinifera* cv. Merlot was performed at DNAnexus (Mountain View, CA, USA). Repetitive regions were marked using the TANmask and REPmask modules from the DAmasker[4] before and after error correction of the SMRT reads. Then, error-corrected reads were assembled with FALCON v.1.7.7[5] using sets of alignment parameters and different thresholds of seed-reads minimum length for overlap stage (length_cutoff_pr parameter). The most contiguous primary assembly was obtained with the parameter ovlp_HPCdaligner_option set to "-k20 -e.96 -s1000 -t32 -l1500 -h150 -mtan -mrep2" and length_cutoff_pr to 4000. Haplotype phasing was performed using FALCON-Unzip with default parameters.

Supplementary Method 2

Additional scaffolding and gap-closing steps were performed on the Cabernet Sauvignon genome assembly. First, genome conformation was assessed using Dovetail™ Hi-C technology (Dovetail Genomics, CA, USA). A Dovetail Hi-C library was prepared as described in Lieberman-Aiden *et al.*[6] and sequenced on an Illumina platform generating 166,370,017 paired-end 150 bp reads (~100X). These data were used to scaffold the Cabernet Sauvignon assembly (after gap-closing with PBJelly) with Dovetail's HiRise™ pipeline v.1.3.0-1233267a1cde[7]. A diploid-aware map of Cabernet Sauvignon was generated using BioNano Genomics technology (BioNano Genomics, San Diego, CA). High molecular weight genomic DNA was extracted from Cabernet Sauvignon leaves by BioNano Genomics (San Diego, CA). DNA was then nicked and labelled using the IrysPrep Kit (BioNano Genomics, San Diego, CA). Labelled DNA was loaded onto the IrysChip nanochannel array for imaging on the Irys system (BioNano Genomics). Imaged molecules longer than 150 kbp were kept. These molecules were assembled using IrysView v.2.5.1.29842[8], generating a 1.05 Gbp consensus genome map with an N50 of 1.49 Mbp. A hybrid genome assembly was produced by combining the Cabernet Sauvignon consensus genome map with its primary contigs and haplotigs using HybridAssembler v.1.0 based on RefAligner v.5678[9]. The relationship between the two haplotype assemblies was defined in micro-synteny blocks. Cross-alignments of Cabernet Sauvignon transcripts (mRNAs) were identified using GMAP v.2015-09-29[10] and parsed with MCSCANX v.11.11.2013[11] to identify collinear regions with a minimum of ten consecutive genes. Sequences lacking micro-synteny were discarded. Next, all scaffolding information obtained by the different technologies were manually combined to generate two haplotype-specific sets of mutually exclusive non-overlapping scaffolds. Finally, chromosome assignment was done based on collinearity with the *Vv vinifera* PN40024 V1 genome assembly (http://genomes.cribi.unipd.it/DATA/) using ALLMAPS v.0.7.5[12] and GMAP alignments of Cabernet Sauvignon transcripts as input. The first set of pseudomolecule-scale sequences (hap1) was the most complete and contiguous and the second set (hap2) all of the alternative phased sequence information. hap2 was refined by using collinearity with hap1 to incorporate unplaced sequences; this was done using HaploSync, a tool developed in-house.

Supplementary Method 3

Genome annotation was performed as described in Vondras *et al.*[13]. Prior to gene prediction, RepeatMasker v.open-4.0.6[14] was used with a custom *Vv vinifera* repeat library[15] to identify and mask repetitive elements. Protein-coding genes were predicted using EVidenceModeler v.1.1.1[16]. *Ab initio* prediction was performed with several predictors (SNAP v.2006-07-28[17]; Augustus v.3.0.3[18]; GeneMark-ES v.4.32[19]; GlimmerHMM v.3.0.4[20]; GeneID v.1.4.4[21]), trained on Cabernet Sauvignon, and Augustus v.3.0.3 trained with a BUSCO dataset. Next, gene models were obtained with PASA v.2.1.0[22] and by using *Vitis* ESTs and flcDNAs (downloaded from GenBank on 03.15.2016), *Vv vinifera* PN40024 V1 coding sequences (CDS; http://genomes.cribi.unipd.it/DATA/), *Vv vinifera* cv. Tannat (TSA GAKH01.1) and *Vv vinifera* cv. Corvina (TSA PRJNA169607) transcriptomes, and Cabernet Sauvignon corrected Iso-Seq reads (SRP132320) as transcriptional evidence. For *Vv vinifera* cv. Merlot, *Vv sylvestris* DVIT3351.27 and O34-16, *de novo* transcriptomes were used as additional transcriptional evidences during gene model identification. Transcriptomes were *de novo* assembled using public RNA-seq data (Merlot: SRR1573033_1, SRR1573034_1, SRR1573035_1, SRR1573036_1, SRR1709051_1, SRR1709052_1, SRR1709053_1, SRR1709060_1, SRR1709061_1, SRR1709062_1; DVIT3351.27 and O34-16: GSE67191 accession). For each genotype, RNA-seq samples were trimmed using Trimmomatic v.0.36[23] with the options LEADING:3 TRAILING:3 SLIDINGWINDOW:10:20 MINLEN:20. Genome-guided transcriptome assembly was performed with StringTie v.1.3.4d[24] and Trinity v.2.6.5[25] using read alignments obtained with Hisat2 v.2.0.5[26] for each genotype separately.

EVidenceModeler v.1.1.1[16] was used to integrate the *ab initio* predictions and PASA gene models with experimental evidence, which included proteins from Swissprot viridiplantae (downloaded on 2016.03.15) mapped with Exonerate v.2.2.0[27] and the aforementioned transcriptional evidence aligned with GMAP v.2015-09-29[10] and BLAT v.36x2[28]. Models featuring in-frame stop codons were removed. Functional annotations of the encoded proteins were assigned based on sequence homology (High Scoring Segment Pair (HSP) length > 50 amino acids) with proteins from the RefSeq plant protein database (downloaded on 2017.01.17) and domain identification by InterProScan v.5.27-66[29]. Structural annotation of the protein-coding genes in SDRs were manually curated. Features with fragmented functional domains were considered pseudo-genes.

For each genome, gene locus nomenclature was adapted from Grimplet *et al.*[30]:

1. Taxonomy ID: the three letters 'VIT' and the code defined by the Vitis International Variety (VIV) Catalogue (http://www.vivc.de/docs/dataonbreeding/AbbrevVitaceae%208Dez10.pdf).
2. Accession ID. For *Vitis vinifera* ssp. *vinifera*, accession ID is composed of the cultivar abbreviation and clone number.
3. Genome assembly version.
4. Genomic sequence.
5. Gene annotation version.
6. Gene numeric code.

Examples:

```
VITVvi_vCabSauv08_v1.1.H0000F_002.ver1.0.g000110
|------------| |------------| |----| |---------------| |-------| |---------|
      1              2          3          4             5         6

VITVvi_sO34-16_v1.1_H0000F_002.ver1.0.g000100
|-----------| |---------| |----| |---------------| |-------| |---------|
      1            2       3          4             5         6
```

Supplementary Method 4

Predicted protein sequences from each genome were compared to one another using BLAST v.2.7.1+[31] and OrthoFinder v.2.3.7[32]. Multiple-sequence alignments of the proteins in single-copy gene orthogroups were done with MUSCLE v.3.8.31[33]. Alignments were concatenated and parsed with Gblocks v.91b, permitting up to 40 contiguous non-conserved positions and blocks no shorter than 5 amino acids[34]. Next, phylogenetic analysis was performed using RAxML-NG v.0.9.0 with the option --opt-model to optimize the evolutionary model in function of the data[35]. We applied the Maximum Likelihood (ML) method with the optimized evolutionary model LG+G8m+F, using twenty random starting trees, a boostrapping of 200 replicates and the Gblocks parsed alignments. The analysis was supervised using the Approximate-ML tree produced by OrthoFinder.

Supplementary References

1. Riaz, S., Krivanek, A. F., Xu, K. & Walker, M. A. Refined mapping of the Pierce's disease resistance locus, PdR1, and Sex on an extended genetic map of *Vitis rupestris* × *V. arizonica*. *Theor. Appl. Genet.* **113**, 1317–1329 (2006).
2. Picq, S. et al. A small XY chromosomal region explains sex determination in wild dioecious *V. vinifera* and the reversal to hermaphroditism in domesticated grapevines. *BMC Plant Biol.* **14,** 229 (2014).
3. Li, P., Ben-Menni Schuler, S., Reeder, S. H., Wang, R., Suárez Santiago, V. N. & Dobritsa, A. INP1 involvement in pollen aperture formation is evolutionarily conserved and may require species-specific partners. *J Exp Bot* **69**, 983–996 (2018).
4. Myers, G. Efficient local alignment discovery amongst noisy long reads. In: *International Workshop on Algorithms in Bioinformatics* 52–67 (Springer, Berlin, 2014).
5. Chin, C.-S. et al. Nonhybrid, finished microbial genome assemblies from long-read SMRT sequencing data. *Nat. Methods* **10,** 563–569 (2013).
6. Lieberman-Aiden, E. et al. Comprehensive mapping of long-range interactions reveals folding principles of the human genome. *Science* **326**, 289–293 (2009).
7. Putnam, N. H. et al. Chromosome-scale shotgun assembly using an in vitro method for long-range linkage. *Genome Res.* **26**, 342–350 (2016).
8. Shelton, J. M. et al. Tools and pipelines for BioNano data: molecule assembly pipeline and FASTA super scaffolding tool. *BMC Genomics* **16,** 1–16 (2015).
9. Staňková, H. et al. BioNano genome mapping of individual chromosomes supports physical mapping and sequence assembly in complex plant genomes. *Plant Biotechnol. J.* **14**, 1523–1531 (2016).
10. Wu, T. D. & Watanabe, C. K. GMAP: a genomic mapping and alignment program for mRNA and EST sequences. *Bioinformatics* **21**, 1859–1875 (2005).
11. Wang, Y. et al. MCScanX : a toolkit for detection and evolutionary analysis of gene synteny and collinearity. *Nucleic Acids Res.* **40**, 1–14 (2012).
12. Tang, H. et al. ALLMAPS: robust scaffold ordering based on multiple maps. *Genome Biol.* **16**, 1–15 (2015).
13. Vondras, A. M. et al. The genomic diversification of grapevine clones. *BMC Genomics* **20**, 972 (2019).
14. Smit, A. F. A., Hubley, R. & Green, P. RepeatMasker Open-4.0. http://www.repeatmasker.org 2013–2015 (2013).
15. Minio, A. et al. Iso-Seq allows genome-independent transcriptome profiling of grape berry development. *G3* **9**, 755–767 (2019).
16. Haas, B. J. et al. Automated eukaryotic gene structure annotation using EVidenceModeler and the program to assemble spliced alignments. *Genome Biol.* **9**, R7 (2008).
17. Korf, I. Gene finding in novel genomes. *BMC Bioinformatics* **5,** 59 (2004).
18. Stanke, M. et al. AUGUSTUS: ab initio prediction of alternative transcripts. *Nucleic Acids Res.* **34**, W435–W439 (2006).

19. Lomsadze, A., Ter-Hovhannisyan, V., Chernoff, Y. O. & Borodovsky, M. Gene identification in novel eukaryotic genomes by self-training algorithm. *Nucleic Acids Res.* **33**, 6494–6506 (2005).
20. Majoros, W. H., Pertea, M. & Salzberg, S. L. TigrScan and GlimmerHMM: Two open source ab initio eukaryotic gene-finders. *Bioinformatics* **20**, 2878–2879 (2004).
21. Parra, G., Blanco, E. & Guigó, R. GeneID in Drosophila. *Genome Res.* **10**, 511–515 (2000).
22. Haas, B. J. et al. Improving the Arabidopsis genome annotation using maximal transcript alignment assemblies. *Nucleic Acids Res.* **31**, 5654–5666 (2003).
23. Bolger, A. M., Lohse, M. & Usadel, B. Trimmomatic: a flexible trimmer for Illumina sequence data. *Bioinformatics* **30**, 2114–2120 (2014).
24. Pertea, M. et al. StringTie enables improved reconstruction of a transcriptome from RNA-seq reads. *Nat. Biotechnol* **33**, 290–295 (2015).
25. Haas, B. J. et al. De novo transcript sequence reconstruction from RNA-seq using the Trinity platform for reference generation and analysis. *Nat. Protoc* **8**,1494–1512 (2013).
26. Kim, D., Langmead, B. & Salzberg, S. L. HISAT: a fast spliced aligner with low memory requirements. *Nat. methods* **12,** 357–360 (2015).
27. Slater, G. S. C. & Birney, E. Automated generation of heuristics for biological sequence comparison. *BMC Bioinformatics* **6**, 31 (2005).
28. Kent, W. J. BLAT---The BLAST-Like Alignment Tool. *Genome Res.* **12**, 656–664 (2002).
29. Jones, P. et al. InterProScan 5: genome-scale protein function classification. *Bioinformatics* **30**, 1236–1240 (2014).
30. Grimplet. J. et al. The grapevine gene nomenclature system. *BMC genomics* **15**, 1077 (2014).
31. Emms, D. M. & Kelly, S. OrthoFinder: solving fundamental biases in whole genome comparisons dramatically improves orthogroup inference accuracy. *Genome Biol.* **16**, 157 (2015).
32. Edgar, R. C. MUSCLE: a multiple sequence alignment method with reduced time and space complexity. *BMC Bioinformatics* **5**, 113 (2004).
33. Castresana, J. Selection of conserved blocks from multiple alignments for their use in phylogenetic analysis. *Mol. Biol. Evol.* **17**, 540–552 (2000).
34. Kozlov, A. M., Darriba, D., Flouri, T., Morel, B. & Stamatakis, A. RAxML-NG: a fast, scalable and user-friendly tool for maximum likelihood phylogenetic inference. *Bioinformatics* **35**, 4453–4455 (2019).
35. Kumar, S., Stecher, G. & Tamura, K. MEGA7: Molecular Evolutionary Genetics Analysis version 7.0 for bigger datasets. *Mol. Biol. Evol.* **33**, 1870–1874 (2016).
