# Supplementary material for: The genetic basis of sex determination in grapes
Source: Nat Commun. 2020 Jun 9;11:2902. doi: 10.1038/s41467-020-16700-z (PMC7283251; doi:10.1038/s41467-020-16700-z)
Supplement: Supplementary file 3 — Reporting Summary [file 41467_2020_16700_MOESM3_ESM.pdf]

natureresearch

Corresponding author(s): Dario Cantu and Brandon Gaut

Last updated by author(s): May 17, 2020

Reporting Summary

Nature Research wishes to improve the reproducibility of the work that we publish. This form provides structure for consistency and transparency in reporting. For further information on Nature Research policies, see Authors & Referees and the Editorial Policy Checklist .

Statistics

For all statistical analyses, confirm that the following items are present in the figure legend, table legend, main text, or Methods section.

n/a	Confirmed	
☐	☒	The exact sample size (*n*) for each experimental group/condition, given as a discrete number and unit of measurement
☐	☒	A statement on whether measurements were taken from distinct samples or whether the same sample was measured repeatedly
☐	☒	The statistical test(s) used AND whether they are one- or two-sided<br>*Only common tests should be described solely by name; describe more complex techniques in the Methods section.*
☒	☐	A description of all covariates tested
☒	☐	A description of any assumptions or corrections, such as tests of normality and adjustment for multiple comparisons
☐	☒	A full description of the statistical parameters including central tendency (e.g. means) or other basic estimates (e.g. regression coefficient) AND variation (e.g. standard deviation) or associated estimates of uncertainty (e.g. confidence intervals)
☐	☒	For null hypothesis testing, the test statistic (e.g. *F*, *t*, *r*) with confidence intervals, effect sizes, degrees of freedom and *P* value noted<br>*Give P values as exact values whenever suitable.*
☒	☐	For Bayesian analysis, information on the choice of priors and Markov chain Monte Carlo settings
☒	☐	For hierarchical and complex designs, identification of the appropriate level for tests and full reporting of outcomes
☒	☐	Estimates of effect sizes (e.g. Cohen's *d*, Pearson's *r*), indicating how they were calculated

Our web collection on statistics for biologists contains articles on many of the points above.

Software and code

Policy information about availability of computer code

Data collection	No software had been used for data collection
Data analysis	ALLMAPS v.0.7.5; Augustus v.3.0.3; BEDTools v.2.19.1; BLAST v.2.7.1+; BLAT v.36x2; BUSCO v.3; BWA v.0.7.17-r1188;ConsensusCore2 v.3.0.0; Cytoscape v.3.7.2; DAmasker; DESeq2 v.1.16.1; Dovetail's HiRise™ v.1.3.0-1233267a1cde; EVidenceModeler v.1.1.1; Exonerate v.2.2.0; FALCON-Unzip v.1.7.7; FALCON-Unzip v.2017.06.28-18.01; GATK v.3.5; GATK v.3.5-0-g36282e4; GATK v.4.1.2.0; Gblocks v.91b; GeneID v.1.4.4; GeneMark-ES v.4.32; GenomicAlignments v.1.12.2; GenomicFeatures v.1.36.4; GlimmerHMM v.3.0.4; GMAP v.2015-09-29; Gviz v.1.20.0; HaploSync (https://github.com/andreaminio/HaploSync); Hisat2 v.2.0.5; HybridAssembler v.1.0; InterProScan v.5.27-66.0; Integrative Genomics Viewer v.2.4.14; IrysView v.2.5.1.29842; JASPAR2018 v.1.1.1; MCSCANX v.11.11.2013; MEGA7; Minimap2 v.2.17; MUMmer v.4.0.0; MUSCLE v.3.8.31; OrthoFinder v.2.3.7; PAML v.4.9; PASA v.2.1.0; PBsuite v.15.8.4; picard-tools v.1.119; picard-tools v.2.0.1; Plink v.2.0; RAxML-NG v.0.9.0; RefAligner v.5678; RepeatMasker v.open-4.0.6; Samtools v.1.9; seqtk v.1.0-r57-dirty; SNAP v.2006-07-28; SSPACE-Longread v.1.1; StringTie v.1.3.4d; TFBSTools v.1.22.0; Trimmomatic v.0.36; Trinity v.2.6.5; WGCNA v.1.66

For manuscripts utilizing custom algorithms or software that are central to the research but not yet described in published literature, software must be made available to editors/reviewers. We strongly encourage code deposition in a community repository (e.g. GitHub). See the Nature Research guidelines for submitting code & software for further information.

Data

Policy information about availability of data

All manuscripts must include a data availability statement. This statement should provide the following information, where applicable:

- Accession codes, unique identifiers, or web links for publicly available datasets
- A list of figures that have associated raw data
- A description of any restrictions on data availability

Data supporting the findings of this work are available within the paper and its Supplementary Information files. A reporting summary for this Article is available as a Supplementary Information file. The datasets generated and analyzed during the current study are available from the corresponding author upon request. Sequencing data are accessible through NCBI under the BioProject ID PRJNA593045 [https://www.ncbi.nlm.nih.gov/bioproject/PRJNA593045]. Genome sequences and gene annotation files are available at https://doi.org/10.5281/zenodo.3827985. The source data underlying Figs 2a–d, 3a–f, 4a–b and 5a and Supplementary Figs 1–5, 8–10 are provided as a Source Data file.

Field-specific reporting

Please select the one below that is the best fit for your research. If you are not sure, read the appropriate sections before making your selection.

☐ Life sciences ☐ Behavioural & social sciences ☒ Ecological, evolutionary & environmental sciences

For a reference copy of the document with all sections, see nature.com/documents/nr-reporting-summary-flat.pdf

Ecological, evolutionary & environmental sciences study design

All studies must disclose on these points even when the disclosure is negative.

Study description	We studied the structure and evolution of the sex determination region in Vitis species. We sequenced and assembled the diploid genome of nine accessions, including three hermaphrodite V. vinifera (Vv) vinifera cultivars, four Vv sylvestris accessions (two females and two males), one male V. arizonica, and one male Muscadinia rotundifolia. Two publicly available genomes from the Cabernet Sauvignon and Zinfandel wine grape cultivars were also included. A phased chromosome-scale genome of Cabernet Sauvignon was generated and used as reference. The twenty-two sex determination haplotypes were compared to study the sex locus structure, its gene content and variability. Gene expression analysis was performed on flower buds from three sex-type accessions at three developmental stages to identify genes which exhibit a sex-linked gene expression pattern.
Research sample	For comparative genomics, grapevine accessions were sampled from the three sex-types covering different subspecies and species of Vitis with diverse genetic backgrounds and from different geographical origins. We sampled five hermaphrodite Vv vinifera: Cabernet sauvignon, Merlot, Zinfandel and two Black Corinth, two female and two males Vv sylvestris, and one male V. arizonica. One male Muscadinia rotundifolia was sampled as a dioecious outgroup.<br>For RNA-seq, floral buds were sampled from the hermaphrodite Vv vinifera Chardonnay (HH) and male and female Vv sylvestris DVIT3351.27 (MF) and O34-16 (FF) at three developmental stages: (i) early development of the reproductive structures, (ii) pre-bloom during pollen maturation, and (iii) anthesis. The three developmental stages were chosen to cover the development of the reproductive structures in grapevine flowers.<br>For marker assay, seven additional hermaphrodite Vv vinifera accessions, two more distant central Asian Vitis species, V. piasezkii (male; MF) and V. romanetii (female; FF), and two F1 populations were selected. One F1 population was the result of a cross between the pistillate Vv vinifera F2-35 (Carignane x Cabernet Sauvignon) and V. arizonica b42-26. The other F1 population was produced by crossing female Vv vinifera 08326-61 (selfing of Cabernet Franc) and Vv sylvestris DVIT3351.27. The two male parents of the F1 populations were chosen to cover two Vitis species.
Sampling strategy	Floral buds were sampled from three inflorescences collected on three individual plants, i.e. one inflorescence per plant, representing three biological replicates per developmental stage. Sampling size was not determined by statistical method but by sample availability in the research vineyard.
Data collection	Melanie Massonnet, Rosa Figueroa-Balderas, Jerry Lin and Summaira Riaz were responsible for scoring sex type and staging sex development and for the collection of specific genotypes.
Timing and spatial scale	Leaves for DNA extraction were collected one time from vines at the University of California Davis (Davis, CA, USA) in February-March 2019.<br>For RNA-seq, inflorescences were collected two times during flowering time: April 4th 2019 and May 7th 2019 from vines at the University of California Davis (Davis, CA, USA).
Data exclusions	No data were excluded.
Reproducibility	All attempts to repeat the data analyses were successful. Allelic state of the candidate male-sterility mutation identified by comparative genomics, i.e. the 8 bp INDEL in INP1, was independently validated in eleven additional genotypes by PCR. The allelic structure of the candidate male-sterility gene was also independently validated by PCR in seven additional hermaphrodite Vv vinifera accessions and two more distant central Asian Vitis species: V. piasezkii (male; MF) and V. romanetii (female; FF), and 365 individuals from two F1 populations. Allelic state of the candidate male-sterility mutation was also validated independently by whole genome sequencing of 4 bulks, two composed of 30 female individuals each and two made of 30 males each, representing a total of 120 individuals from the population Vv vinifera F2-35 x V. arizonica b42-26.

Randomization	Not applicable.
Blinding	Not applicable.

Did the study involve field work? [x] Yes [ ] No

Field work, collection and transport

Field conditions	On April 4th 2019: Partly sunny, temperature: 16°C, humidity: 67%.<br>On May 7th 2019: Scattered clouds, temperature: 21°C, humidity: 50%.
Location	Tissues were collected from grapevines located in research vineyards of the University of California Davis (Davis, CA, USA) at 38.532485° N, 121.752017° W and 38.528458° N, 121.793350° W, and from the Russell Ranch Foundation Vineyard (Foundation Plant Services, Davis, CA, USA, 38.542654° N, 121.872561° W).
Access and import/export	Not applicable. All genotypes are publicly available in germplasm collections located in Davis (Foundation Plant Services and University of California Davis, CA, USA). Clone and accession numbers of the genotypes are listed in Supplementary Table 1.
Disturbance	No disturbance was caused during the study

Reporting for specific materials, systems and methods

We require information from authors about some types of materials, experimental systems and methods used in many studies. Here, indicate whether each material, system or method listed is relevant to your study. If you are not sure if a list item applies to your research, read the appropriate section before selecting a response.

Materials & experimental systems

n/a	Involved in the study
[x]	[ ] Antibodies
[x]	[ ] Eukaryotic cell lines
[x]	[ ] Palaeontology
[x]	[ ] Animals and other organisms
[x]	[ ] Human research participants
[x]	[ ] Clinical data

Methods

n/a	Involved in the study
[x]	[ ] ChIP-seq
[x]	[ ] Flow cytometry
[x]	[ ] MRI-based neuroimaging
