# Supplementary material for: The genetic basis of sex determination in grapes
Source: Nat Commun. 2020 Jun 9;11:2902. doi: 10.1038/s41467-020-16700-z (PMC7283251; doi:10.1038/s41467-020-16700-z)
Supplement: Supplementary file 4 — Description of Additional Supplementary Files [file 41467_2020_16700_MOESM4_ESM.pdf]

Description of Additional Supplementary Files

File name: Supplementary Data 1
Description: Summary statistics of genome sequencing and assembly of the eleven grape accessions.

File name: Supplementary Data 2
Description: Coordinates and features of the sex-linked SNPs.

File name: Supplementary Data 3
Description: Coordinates and features of the sex-linked INDELs.

File name: Supplementary Data 4
Description: Results of the marker assay performed on the F_1 population *Vv vinifera* F2-35 x *V. arizonica* b42-26.

File name: Supplementary Data 5
Description: Results of the marker assay performed on the F_1 population *Vv vinifera* 08326-61 x *Vv sylvestris* DVIT3351.27.

File name: Supplementary Data 6
Description: Sex-linked transcription factor-binding motifs within 3 kbp region upstream of transcription start sites of each gene composing the sex-determining locus.

File name: Supplementary Data 7
Description: Differential gene expression analysis of the genes within the sex-determining locus of *Vv vinifera* cv. Cabernet Sauvignon.

File name: Supplementary Data 8
Description: Differential gene expression analysis of the alleles within the sex-determining locus of *Vv sylvestris* DVIT3351.27.

File name: Supplementary Data 9
Description: Gene co-expression modules obtained from Weighted Gene Coexpression Network Analysis (WGCNA).

File name: Supplementary Data 10
Description: Summary of RNA-seq data features of floral buds collected from *Vv sylvestris* O34-16 (female), DVIT3351.27 (male) and *Vv vinifera* cv. Chardonnay at three developmental stages.
