# Supplementary material for: The genetic basis of sex determination in grapes
Source: Nat Commun. 2020 Jun 9;11:2902. doi: 10.1038/s41467-020-16700-z (PMC7283251; doi:10.1038/s41467-020-16700-z)
Supplement: Supplementary file 2 — Peer Review File [file 41467_2020_16700_MOESM2_ESM.pdf]

Reviewers' comments:

Reviewer #1 (Remarks to the Author):

Sex determination is a major switch in the evolution of dioecious plants and animals. It used to be and still is in many species a major effort to sequence and assemble sex chromosomes and identify and characterize candidate genes for sex determination. The authors fully utilized the rapidly advancing genomic technologies and generated 9 phased genomes of male, female, and hermaphrodite. They have compared male and female or hermaphrodite and female haplotypes in three species and an out group species in a sister genus. Based on comparative genomics and gene expression analysis, they identified INP1 and YABBY as candidate genes for sex determination, corroborating previous reports about these two candidate genes.

An inversion was detected in the sex determination region (SDR) in M. rotundifolia, but not in any Vitis species that examined. This is odd for two reasons: 1. They have proven that sex chromosomes are ancestral in the genus Vitis, because all M haplotypes across species were clustered together, separated from all F haplotypes from multiple species, a valid and strong test. For a stable sex chromosome system to be inherited through all ~70 species after 47 million years of divergence, the SDR had to be suppressed for recombination. Otherwise sex reversal to hermaphrodite would occur in every generation, as we as the appearance of neuters. 2. The M haplotype (425.9 ± 274.6 kbp) have expanded more than twice, some three times the size of the F haplotype (181.4 ± 10.2 kbp), likely by retrotransposon insertions. This level of sequence expansion in SDR of the Y chromosome is only possible if the recombination in SDR is suppressed, either by inversions (most common) or another mechanism.

The best approach to define the genetic border of SDR is genetic mapping using an interspecific F1 population segregating for male and female. Hope such maps were available or a segregating population is available for a quick mapping using RADseq or skim sequencing of the individuals in the mapping population. The authors used flanking markers of the sex locus from a published genetic map, likely recombining between H and F haplotypes. This may explain that the right border of SDR in Figure 2 is likely partial, not reaching the end, not the full scale of the SDR region. The authors do have genomic resources to define molecular borders of the SDR by comparing the entire contiq containing the M haplotype and F haplotype in each species. The N50 of the genome assemblies is about 1.9 Mb, large enough to cover the entire region. By aligning the homologous sequences of the pseudo-autosomal regions, it will reveal the actual sizes of M and F haplotypes, and whether inversions were present in these Vitis species. A new Figure 2 showing SDR flanked by pseudo-autosomal regions in each species will clarify these questions.

The separate clustering of M and F haplotypes indicates that Vitis has sex chromosomes and they are ancestral in this genus. What about the YY genotypes? Are they viable? It will determine the stage of Vitis sex chromosome evolution.

The authors have done excellent work and generated abundant genomic resources. Improved clarity and resolution in figures and text will enhance the quality and readability of this manuscript.

Minor changes:

Please add line numbers

It would be better to clearly state in Results and Methods that the genome sequences were generated by PacBio platform, not just SMRT DNA sequencing. Which platform? RSII, Sequel I, or Sequel II? Please add to the text.

Page 1 affiliation 2: Delete extra “Dept.”

Page 2 line 2: "… sex determination (SD) region…" - … sex determination region (SDR) …

Page 3 paragraph 2 line 1: "… economic importance of dioecy…" – dioecious crops are economically important, but most dioecious species are not crops.

Page 3 paragraph 3 lines 3 - 7: All 70 species are dioecious, but one can not conclude dioecy is ancestral from that observation. You proved it in your study by analysis of M and F haplotypes. Perhaps change "indicating" to "hinting".

Page 3 paragraph 4 lines 1 – 2: It shouldn't be the assumption that Vitis doesn't have sex chromosomes, and your results disproved it.

Page 4 paragraph 2 lines 2 – 3 from bottom and Page 14 paragraph 4: If Vitis has no sex chromosomes or no recombination suppression in SDR, hermaphrodites will appear after every meiosis, and it wouldn't be hard to select during domestication. But your data disproved both assumptions. It is unlikely that hermaphrodites were derived from recombination, more likely from chromosomal rearrangement.

Reviewer #2 (Remarks to the Author):

GENERAL COMMENTS

There are a few valuable bits of information in this ms, but it is severely marred by poor presentation, including lack of clear explanations (despite space being used for repeating things), poor English, absence of statistical test for claims, vagueness, and, worst of all, failure to put the results and conclusions in a context of previous understanding of sex determining regions, including those of plants. Many parts of the ms are undigested descriptions of results, and the reader is not told why they are being described or helped to understand what they tell us about the development or evolution of separate sexes in this plant, or more generally. Dissecting out the valuable information from this morass of description, it seems that the study may have done PacBio long-read sequencing that allowed them to determine X- and Y-linked haplotypes of a region of the grapevine chromosome 2 (of about 1 Mb between roughly 4.8 and 5.8 Mb of the previously published grapevine female assembly) in which the sex determining locus has been genetically mapped (the text uses the term "sex locus" instead of the correct term "sex determining locus", and this should be corrected throughout). Then, examining predicted genes within this region, the authors try to define the extent of a fully sex-linked region, and then to describe some candidate sex determining genes. All of these parts need revision, some of them including not just better organisation and explanation, but also better analyses. Space for better explanations can be made available by avoiding repetitions. Much of the Discussion section repeats results and approaches, not much more clearly than in the earlier parts, and it would be a good idea to use this section to tell us what has been understood about the questions of interest (see below).

I apologise if I have misunderstood some things, but, after a great deal of effort to read it, I found the ms quite confusing.

Overall, the methods used in this study are not well explained in the main text. It was not even made clear that the entire study is based on new PacBio sequencing. The study did not include any genetics, but relies on sequence data, which can indeed yield evidence that some variants are male- or female-specific (respectively suggesting Y or X linkage), provided that appropriate sample sizes of independent individuals are genotyped. The ms is vague about sample sizes.

In my opinion, the network analysis is inconclusive, and the conclusions speculative, and it should be

removed (or published separately in a journal where it can be assessed by experts in such analyses).

DETAILED COMMENTS
It is not at all remarkable among plants that Vitis does not have sex chromosomes, by which the authors mean heteromorphic sex chromosomes, but just a sex determining SD region or locus. At least half of dioecious plants that have been studied have such homomorphic "sex chromosomes", which might better be termed "fully sex-linked regions". It is well understood that these must have sex-specific haplotypes, and several recent studies have examined such regions in detail, for example in papaya (which has slight heteromorphism), asparagus, poplar and kiwifruit (with no heteromorphism detectable cytologically, although length differences have now been detected in the region in some of these). In all of these species, males are the heterozygous sex (male and female haplotypes are usually called X and Y, even though they are not entire heteromorphic sex chromosomes; the notation MF in this ms could be used, if wanted, but ought to be explained if it is used). Vitis is not the only plant in which cultivated 'strains' are hermaphrodites. Other examples are papaya and probably spinach, Actinidia and strawberries. In Vitis and Spinacia, Westergaard reviews evidence that some hermaphrodites segregate with 3 staminate: 1 female ratios, suggesting Y-linkage (and an inviable YY genotype, suggesting that the Y-linked region may be ancient enough to have lost some functions), while others appear to have autosomal factors. It is possible that different hermaphrodites evolved independently through different genetic mechanisms, or perhaps that some hermaphrodites evolved long enough ago that the initial mutation of a Y-linked gene has been followed by an autosomal mutation that might improve female (or perhaps male) function. It would be good to mention that Westergaard's 1958 suggested that the two gene model, including having an active Y-linked male-determiner, might apply to Vitis (as opposed to a system involving gene interactions that can result in a single gene system), and mentioned the hermaphrodites, along with reviewing other similar cases (see above). In this context, it is worth mention that the phrase "Genetic evidence for the two-locus model is not universal" is unsatisfactory. It implies that a different model, involving just a single gene, could lead to the evolution of dioecy. However, this overlooks the fact that mutations in two genes are necessary, but can result in just a single segregating locus, given suitable interactions that allow one of the mutations to fix in the species. This is clearly what has happened in Diospyros — the obeservation of a single gene does not mean that only a single mutation was required, and this can be seen in the paper by Akagi et al. that is cited by this ms, although the authors do not explain clearly the concept just mentioned.

In papaya, the hermaphrodites have long been known to carry "X" and "Y" chromosomes, in which the "Y" has lost the female-suppressor. This, and other similar situations, are reviewed in Westergaard's 1958 review in Advances in Genetics, vol. 9, pages 217-281. The ms mentions none of this background. The ms is, in general, very weak in describing the state of knowledge prior to the new work. Previous understanding of the sex determining region in cultivated grapevines is poorly explained, but (in my edited wording, which needs to be amplified in the region indicated by []), it seems that " The Vv vinifera fully sex-linked region has been delimited to a ~200 kbp region of chromosome 2 (Fechter et al., 2012; Picq et al., 2014; Zhou et al., 2019b), and current evidence [WHOSE NATURE SHOULD BE BRIEFLY OUTLINED HERE so that readers can see what is new from the results in this new study] supports the two-locus model. One new result would be if the sex-determining genes were identified, which they were not previously, although the 15 to 20 genes within the region are candidate SD genes (Dalb. et al., 2000; Riaz et al., 2006; Marguerit et al., 2009; Fechter et al., 2012; Battilana et al., 2013; Hyma et al.,2015; Zhou et al., 2017)".

If the aim of the study is to test the two-gene model better for Vitis, using the latest sequencing and assembly methods that have now become available, this should be stated, along with stating the approach to be used. I think the authors believe that this can be done by sequencing and identifying candidate genes. However, of course, if there is a completely non-recombining region containing as many as 15 genes, it is unlikely to be possible to discover which of them is involved, because sequence variants may be present in several of them, some of which lead to loss of one sex functional or the other, while other fully Y-linked variants may simply be mutations (perhaps without functional

significance) that occurred in the Y-linked since it stopped recombining with the X-linked homologous region. If the fully sex-linked region includes genes with functions that strongly suggest their involvement in sex determination, then this is certainly an advance (though their functional involvement needs to be tested, for example in transgenic experiments, which are clearly beyond the scope of this study). This study did identify a candidate male-sterility mutation (the inaperturate pollen gene), but its expression is higher n females than males, making the evidence that it is an actual SD gene less clear.

Overall, therefore, the sex determining genes have not yet been definitively identified, and it is not yet possible to say whether two genes in the region identified were indeed involved in the evolution of separate sexes. It would be helpful to understand what is known about the time when dioecy evolved in the genus Visit, or an ancestor of the species studied. Clearly, a first step is to find out whether there really is a completely sex-linked region, and to estimate its age. Then the genes in the region can be identified and tested. If dioecy is ancient, then the idea that genes in the sex-linked region may have evolved changes since the SD mutations/system became established might be plausible (see also below). I have divided my comments into sections dealing with the different questions, in some kind of logical order.

QUESTION 1: Is there a completely non-recombining region?
The Methods section does not give adequate detail about important aspects such as the phasing. The ms does not clearly describe the nature of the sequencing approach used, other than "Single Molecule Real Time (SMRT) DNA sequencing" (which I think means PacBio), and the statement that the results were phased (e.g. parameter values used), without any information about the sequencing quality, how the results were validated or how the boundaries of the putative fully sex-linked region (or SD region) were determined, and whether there is a clear cut-off at each end. This information is important for assessing whether the complete region has been reliably identified, and whether it is truly non-recombining, or recombines rarely (as suggested when discussing the origin of hermaphrodites in domesticated cultivars). The sample sizes are also not entirely clear. The text mentions 22 haplotypes of the SD region, but 9 accessions were sequenced (presumably 18 haplotypes). Maybe the number 22 includes the outgroup species that was sequenced (see also below)? This sample size is rather small for establishing statistically significant associations between the sex and sequence variants, especially in cultivated material, where it is possible that one cultivar is descended from another, so that associations are not equivalent to those based on studying a sample from a single natural population. Even without common ancestry of this kind, a bottleneck during domestication will produce elevated linkage disequilibrium, so that associations may be false positives. The text should give some consideration to these points. Ideally, any potentially interesting associations should be validated using a natural population sample, or (in a cultivated species, a set of cultivars derived independently from the progenitor). The ms does describe tests in 2 segregating families, which do support sex linkage, but cannot demonstrate that rare recombination is absent. If the ideal sample cannot be studied, or the wild progenitor is now extinct, this issue should be discussed.

The purpose of the neighbor-joining phylogenetic trees for each gene is not clear, but it presumably related to this question, since, if the genes are fully sex linked, the phylogenies should be very similar. In Figure 3, one can see that several genes (from the TPP gene to the left of the candidate male-sterility gene with the stop codon causing inaperturate pollen, INP1, to the 7th gene on its right, VviFSEX) yield trees in which males and hermaphrodites form a single cluster (similar to the papaya situation in which hermaphrodites are probably mutant males whose Y-linked region has lost the female suppressor), while the other genes, at the two ends of the region analysed, do not evidently agree with the statement above. This appears to suggest that those 'flanking' genes might be partially, not fully, sex-linked, although they might show associations (linkage disequilibrium) with the fully sex-linked region. It would be good to integrate information so that readers know which regions fall into each of these categories. Perhaps the authors are trying to say that the sequences allow them to infer this, but this is not clearly communicated.

Another odd decision concerning the tree analysis is not to include the outgroup species. The trees are therefore unrooted, and rooted trees would have been preferable, with bootstrap support values. If dioecy pre-dates the split between Vitis and Muscadinia, they could well have the same clusters of Y-linked sequences, as has been found in other cases of genetic polymorphisms that pre-date a species split. The ms indeed mentions that "the same 8 bp deletion was also found in the female M. rotundifolia, [so] this F-specific INDEL likely occurred before Vitis and Muscadinia diverged". As both species are dioecious, it is also likely that the female suppressor may also be present in the latter, and the tree in Fig. 1 suggests that these are not too highly diverged to try this analysis (it would be good to mention the synonymous site divergence between a sample of genes in these species).

It is potentially of interest that the Y-linked region is inverted in the outgroup species, M. rotundifolia, as the two-gene model for the evolution of separate sexes suggests that fully Y- and Y-linked regions evolve in 3 steps, first, the spread male- and female-sterility mutations at two separate, but closely-linked sites on a chromosome, and then the evolution of closer linkage (this will prevent rare X-Y recombination events, which will generate hermaphrodites and, even worse, neuter phenotypes, which are sterile). The observed inversion could reflect a rearrangement like that proposes as step 3 (indeed the ms suggests that X-Y recombination may have allowed reversion to hermaphroditism in Vitis). This inversion would presumably have had to happen after the split from Vitis, and appears not to have occurred in Vitis (unless inverted arrangements in Vitis have not become fixed, and have not been detected in the limited sampling of this species). The observation of an inversion that is limited rather precisely to the sex-determining region is another potentially interesting result of this sequencing, but the ms does not really discuss what it tells us, or might tell us, given further study. The ms mentions that the Vitis SD haplotypes differ in length (though it is not very clear which regions differ and what the differences are — for example, are the Y introns longer than the X versions, or are transposable elements detected in non-coding regions (Fig. 1 shows that Y haplotypes seem to be consistently longer than their X counterparts from the same species, and that inter-genic regions are expanded, although the sample sizes are small)? Some detail might be helpful. The ms also suggests that such length differences may contribute to suppressing recombination, but does not consider which is the cause and which the effect. An inversion is clear, because one can infer the ancestral stare, and it does indeed have the potential to suppress recombination, especially if only a small region is inverted.

QUESTION 2: It is a separate question to ask "Can the sex-determining genes be identified?"

Rather than looking at trees, it might be better simply to show which sequence variants are restricted to males, suggesting Y-linkage (although, as explained above the sample sizes are very small for any strong conclusions). Such variants should show the genotype configurations expected under the form of sex system seen, for example XY regions should include male-specific variants, indicating Y-linkage, and one can test (given an adequate sample) what proportion of variants in a window of given size show the expected patters with all males heterozygous and all females homozygous for the X-linked allele. Given the phasing analysis, the trees may be a redundant analysis.

It is stated that the haplotypes tended to cluster by sex, but no test is described. It is not clear why this test was not done for the entire fully sex-linked region, as this would be best tested using all variants in the region. It is also not explained why the alignments were concatenated, as one would expect each gene to be represented within a long read, assuming that long-read sequencing was done (see above).

First, however, it is also not completely clear whether each individual's genome sequence was independently assembled, meaning that each sex's SD region was assembled separately, or whether the Cabernet Sauvignon reference assembly was used. It was used for some analyses, and it is not entirely clear which ones. It is also not clear which sex was used, as this cultivar is stated to have females and hermaphrodites. If a female reference genome was used to map the new reads, or help assemble them, is there any risk that sequences specific to the Y-linked region could have failed to be

detected? This should be discussed clearly.

Before tackling question 2, it would be helpful to separate it into distinct tests, as follows
(i) Examining just males and females, are some variants male-specific, suggesting Y linkage? If so, can one identify candidate male-sterility mutations in the X haplotype, and female-suppressing ones in the Y haplotype? Only after this is clear should the hermaphrodites be examined, as explained next.
(ii) For sequences within the fully sex-linked region, are hermaphrodites' sequences more similar to those of males' Y alleles, or to X-linked alleles? If the former, this suggests that (as in the papaya Yh chromosome) hermaphrodites have a modified Y that has lost the female suppressing factor carried by "true" Ys.
(iii) The result of (ii) is important, because the comparison between Y and Yh regions can help test candidate genes — specifically, if Yh has lost the female suppressing factor, or has a non-functional copy, and this occurred recently (as the text suggests), this could be used to test whether a candidate gene is plausible. Moreover, it would also increase the sample size of Y haplotypes, because, apart from this mutation, the rest of this region should be a normal Y that can be compared with X haplotypes.

Instead of a clear analysis, with clear steps, the ms confusingly describes comparisons between all 3 haplotypes, making it very difficult to understand what the results tell us. It is nevertheless hopeful that, among the plethora of sequence variants (SNPs and indels) described, some are candidates for involvement in sex determination.

Specifically, a frame-shift that results in production of a truncated protein may represent the male-sterility mutation that created females from a presumably hermaphroditic ancestor (the ms also fails to mention what is known about the likely ancestral state, and it mentions that dioecy has been maintained for a long time, without explicit details; these should be added, including synonymous site divergence values). However, this gene was found to be transcribed at a higher level in females than in males, making its involvement unclear. Presumably this is based on estimates of transcript abundances, and no details are provided about how the two different transcripts were measured (do they behave the same in the assay that was used, or does the stop codon affect the transcripts, or their abundance?). Details of the numbers of replicates, and the methods, are also missing or vague.

A candidate female-suppressing mutation was also examined, but no candidate becomes clear, if I understood correctly (as mentioned, the ms is very difficult to follow). The Discussion section says that "there are several candidates for the sp mutation", and seems to focus on the male-sterility mutation candidate, and says little about the female-suppressor. The text on p. 13 says that "F-linked polymorphisms define a region of the SD that is likely to house the hypothesized recessive sp mutation, because this is where F haplotypes differ from the M and H haplotypes that retain male function. I found this confusing, because I think that this criterion would define the region associated with the difference between males and females (test (i) above), and not the region carrying the female-suppression mutation (So, in the authors' notation). Moreover, it would be true only if recombination occurs (as explained above, associations within a completely linked region cannot be used for such inferences). A previously proposed candidate, APT3, seemed promising because it is expressed in the carpel primordia of male plants, consistent with a role in pistil abortion (and the new expression results on p. 11 support this), but the trees in Fig. 3 now seem to suggest that this is not fully sex-linked. However, this should be checked by a more refined analysis suggested above, comparing just males and hermaphrodites, as tree analysis could be misleading for such a conclusion. The ms mentions that mutants of Arabidopsis [presumably thaliana] APT1 [presumably the A. thaliana ortholog of the APT3 gene in Visit?] "are sterile males" [presumably meaning male sterile]. It does not tell the reader at all clearly what the APT3 results tell us until p. 16, where (after a welter of network analysis results) it is discounted as a candidate for "either the sp or the So mutation". Finally, the WRKY transcription factor is mentioned, and the support for this candidate is that it has lower expression in females than males (and this gene does seem from Fig. 1 to be potentially fully sex-linked), but, confusingly, page 12 discusses whether "WRKY plays a role in male sterility".

Another potential barrier to understanding is that the ms uses the non-standard notation just quoted, deriving from a paper in 1838 by Oberle, which is likely to be inaccessible to many readers (N. Y. Agric. Exp. Sta. Tech. Bulletin). In my view, it is not important enough to recognize Oberle's contribution by adopting his notation, given that Westergaard reviewed many species and unified the notation, and his notation (or minor variants of it) has been widely used since then. Even with a notation that some readers will know, it is difficult to remember which mutation is which, and in many places, it would be better if the text simply said "male-sterility" or "female-suppressing".

Minor comments
1. Is it correct that the majority of animals have separate sexes? I have been told that hermaphrodites are commoner than gonochoristic species, so this should be checked.
2. Of course, Darwin pointed out, long before Renner (2014), that dioecy is rare in plants. And the evidence that it has evolved many times in plants, independently in different lineages, was also known long before Renner's article (e.g. Westergaard's 1958 review in Advances in Genetics, vol. 9, pages 217-281, which is unaccountably not mentioned, and Charlesworth's 1985 analysis of the distribution of dioecy and self-incompatibility in angiosperms, pp. 237-268 in Evolution — Essays in Honour of John Maynard Smith, edited by P. J. Greenwood and M. Slatkin).

Reviewer #3 (Remarks to the Author):

In general, this is a well written and interesting research paper. The discussion is very well supported with results and the methodology robust. These are, however, some small questions and remarks. First of all, authors must consider to review the Vitis nomenclature used. For that we advise to read "The grapevine gene nomenclature system; BMC Genomics volume 15, Article number: 1077 (2014)".

Page 3:
"The second step is a dominant mutation that suppresses female function (So)"
Should be: "The second step is a dominant mutation that suppresses female function (So), also accordingly to Oberle, 1938)"

"Thus far, the best candidates come from asparagus, where females lack a gene associated with tapetal development (Tsugama et al., 2017) and a mutant male lost a putative female suppressor (So) gene to become a hermaphrodite (Harkess et al., 2017)."
The So reference in this phrase should be eliminated. The So/so Sp/sp model was build specifically for the grapevine dilemma and has not referred in the work of Harkess et al., 2017.

Page 4:
on «With these extensive data, we address four questions»
Only three questions are raised by authors. Shortly:
1) How M differs from F and H?
2) Are there different gene expression levels?
3) Can we reconstruct the domestication process?

Figure 1: The c panel on figure 1 does not fit together with the others panels within this figure. Authors are advised to review the images positioning.

Page 5:
"male Muscadinia rotundifolia" and "400 kbp insertion, and the M. Rotundifolia"
Is a bit confusing, either "male muscadine" or "male V. rotundifolia". This issue is recurrent as it appears throughout the manuscript

“three hermaphrodite Vv vinifera”
Would benefit having the cultivars names: “three hermaphrodite Vv vinifera cultivars (Merlot, Black Corinth seedless and Black Corinth seeded)”

“Relative to H and Vv M haplotypes,”
Should be: “Relative to H and M haplotypes,”

Page 12:
A blank line is missing between the legend of Figure 5 and the manuscript text.

Page 13:
Ramos et al 2014 has a similar diagram with fig. 6. How do your findings relate to the hypothesis raised by these authors? Or vice versa.
There is also some talk about the So and Sp but little regarding to what they mean. When talking about figure 6, would be nice to have some background regarding the so/So and sp/Sp and its origin. Also interesting would discuss why based on these findings a model like so/So; sp/Sp is more adequate than for example the M F H model. Despite the terminology M, F, H being used for the haplotypes for the sake of clarity.

Page 15:
When the authors question why the haplotypes remain distinct, one simpler hypothesis, along with the possibilities raised by the authors, is the one point by Oberle when he suggested the two close linked loci. In the case of this work, if YABBY and INP1 are close then recombination between the two loci would be rare.

F1 populations:
What were the segregation rates (and $\chi 2$) of the F1 populations?
If we counted correctly, one cross resulted in 100 females and 118 males and the other cross in 78 females and 100 males.
There is also some confusion regarding the F1 offspring phenotype (supplementary table 4a and 4b). What is the meaning of the NA plants, they don’t have a visible phenotype yet (haven´t yet bloom) or INP1 amplification was inconclusive?

The amplification of INP1 in the F1 populations showed that male plants had at least one functional INP1 allele. What about the YABBY gene? Did the SV in the YABBY gene and promoter segregate in the F1 according to sex?

Two F1 populations are a remarkable source of information that the authors should explore. Mainly in order to confirm the hypothesis proposed and see how they hold compared with older Mendelian studies (Oberle 1938; Avramov et al 1967; Negi and Olmo 1971, for example)

Supplementary figure 7:
In the panel B there is alleles F and M but from what we understand from the manuscript, female plant is FF and so the alleles would be FF and not F H as it is shown in the figure. Similarly, male plants would be MF and not HF was inferred by the figure.

This article was reviewed by: Margarida Rocheta; Lucas Coito and Miguel de Jesus Nunes Ramos from the same group.

Reviewer #4 (Remarks to the Author):

The authors studied the structure and evolution of the sex determination region in grape species to

better understand the origin of dioecy and how dioecy was lost through the domestication. They identified a candidate male-sterility mutation in the INP1 gene and potential female-sterility function associated with a transcription factor. Moreover, the identification of two candidate genes encourage future testable hypotheses concerning their putative evolution and function.
This is an interesting manuscript which provides new insights on the domestication process in grapevine. The manuscript is clear for most parts and pleasant to read. I do not have experience with some analysis implemented in the manuscript. However, the choice of methods seems appropriate and the methodology is adequate. The results are in line with the aims and show which genes are probably involved in the domestication process.
I have some specific comments that show below (I am surprised not to see the numbers of the lines along the manuscript. This is very unpleasant. However, I have indicated the number of the line to which my comments refer, hoping to use the same pdf file.)

Title: Your study involves different species from Vitis vinifera to Muscadine. Usually, the grapevine is referred to Vitis vinifera L. I suggest changing the word grapevine with grapes, in the title.

Introduction: Page 3, Line33. I have some doubts about the time of the split of the genus Vitis. Effectively, Ma et al., 2019 show a split about 47 millions of years ago but this result is influenced by calibration models, choose of outgroups and fossil data. In particular, divergence times estimations can be affected by interpretation of fossil data and the number of calibration points applied (Rutschmann et al. Syst Biol 2007; Zecca et al., Current genetics 2019). As you can see the paper table1 in Wan et al., (BMC Evolutionary Biology 2013) are recognized different times of split for the crown of Vitis subgenus. Moreover, other values are recognized in more recent papers. Thus, I suggest that the authors should avoid showing the time of divergence of the Vitis, unless you don't choose to show the alternative results.

Results: Page 5, line 6. You have rooted the tree with M. rotundifolia. I agree but you should add a reference about phylogenetic studies and a short comment showing that this species is distant from all other grapes. If you need references, see GRIN taxonomy or Flora of North America.

Discussion: Page 13, Line3. I expected to read a profound discussion about the YABBY gene. The function of this gene is widely discussed for other species; you should have no problem finding articles about it. In addition, there are recent articles also related to the genus Vitis (Zhang et al., Frontiers in Plant Science 2019; Xiang et al., Protoplasma 2013). I suggest you, to compare your results with other studies and so to improve the discussion.

Discussion: Page 13, Line4. “Among all protein phylogenies, YABBY clearly differentiates M haplotypes from F and H haplotypes”. I don’t agree. There are other protein phylogenies that show clearly differentiates M haplotypes from F and H. The authors should modify the sentence.

Discussion: page 14, line23. I agree with the authors that the highly expressed of INP1 gene in female is a mystery. However, also your answer is a mystery for me ."...High expression of INP1 in females could reflect an attempt to compensate for the 8 bp deletion that renders the protein non-functional." I don’t understand how a cell can compensate for this. This sentence should be explained better and highly supported by data and references.

Discussion: page 14, line10. I don’t understand where to find the 60% value in your results. Perhaps, do you mean 57%?

Discussion: page 14. “..Length differences between F and M haplotypes play some role in recombination deterrence,..” This is very interesting. Are there other studies that support this? Can you support this hypothesis with adequate literature?

Materials and methods: pag19, Phylogenetic analysis. You have applied the Maximum-Likelihood (ML)

method, assuming the evolutionary model LG+G8+F. How have you chosen the model? Do you have used software? You should indicate the name of the software and how you have set it.
.
Fig. 3: For me is very difficult to find the NP1 gene in Figure. Can you find a better solution to indicate this gene in Figure?

Response to Reviewers – Authors' answers are in blue

Reviewer #1 (Remarks to the Author):

Sex determination is a major switch in the evolution of dioecious plants and animals. It used to be and still is in many species a major effort to sequence and assemble sex chromosomes and identify and characterize candidate genes for sex determination. The authors fully utilized the rapidly advancing genomic technologies and generated 9 phased genomes of male, female, and hermaphrodite. They have compared male and female or hermaphrodite and female haplotypes in three species and an out group species in a sister genus. Based on comparative genomics and gene expression analysis, they identified INP1 and YABBY as candidate genes for sex determination, corroborating previous reports about these two candidate genes.

An inversion was detected in the sex determination region (SDR) in M. rotundifolia, but not in any Vitis species that examined. This is odd for two reasons: 1. They have proven that sex chromosomes are ancestral in the genus Vitis, because all M haplotypes across species were clustered together, separated from all F haplotypes from multiple species, a valid and strong test. For a stable sex chromosome system to be inherited through all ~70 species after 47 million years of divergence, the SDR had to be suppressed for recombination. Otherwise sex reversal to hermaphrodite would occur in every generation, as we as the appearance of neuters. 2. The M haplotype (425.9 ± 274.6 kbp) have expanded more than twice, some three times the size of the F haplotype (181.4 ± 10.2 kbp), likely by retrotransposon insertions. This level of sequence expansion in SDR of the Y chromosome is only possible if the recombination in SDR is suppressed, either by inversions (most common) or another mechanism.

The best approach to define the genetic border of SDR is genetic mapping using an interspecific F1 population segregating for male and female. Hope such maps were available or a segregating population is available for a quick mapping using RADseq or skim sequencing of the individuals in the mapping population. The authors used flanking markers of the sex locus from a published genetic map, likely recombining between H and F haplotypes. This may explain that the right border of SDR in Figure 2 is likely partial, not reaching the end, not the full scale of the SDR region. The authors do have genomic resources to define molecular borders of the SDR by comparing the entire contiq containing the M haplotype and F haplotype in each species. The N50 of the genome assemblies is about 1.9 Mb, large enough to cover the entire region. By aligning the homologous sequences of the pseudo-autosomal regions, it will reveal the actual sizes of M and F haplotypes, and whether inversions were present in these Vitis species. A new Figure 2 showing SDR flanked by pseudo-autosomal regions in each species will clarify these questions.

We thank the reviewer for the suggestions. To address the reviewer's comments, we have generated new sequencing data, carried out additional analyses, modified the manuscript, and added new figures. We think that these changes helped improve clarity and strengthen our conclusions.

First, we have expanded the description on how the sex-determining region (SDR) was identified in the Cabernet Sauvignon genome using known genetic markers. Known sex-linked markers were added in the main text and in **Fig. 2** and **Fig. 3**.

Second, we have extended the variant analysis on a larger genomic region, comprising the SDR flanked by pseudo-autosomal regions (**Supplemental Fig. 2**). The absence of sex-linked SNPs outside of the SDR confirmed the location and the boundaries of the locus.

Finally, we followed the reviewer's suggestion and took advantage of the interspecific F1 population (*Vv vinifera* F2-35 (FF) x *V. arizonica* b42-26 (MF)) we used in the study. We sequenced the genotypes of 120 progeny divided in four bulks based on sex type. The bulk segregant analysis confirmed the location of the locus on chromosome 2 and confirmed the complete linkage between the sex-linked polymorphisms we have identified by comparing phased haplotypes, including the non-synonymous mutations in *VviYABBY3* and the 8 bp deletion in *VviINP1* (**Supplementary Fig. 8**).

In addition to the new figures (**Supplementary Fig. 2** and **Supplementary Fig. 8**), the following changes were made to the manuscript:

<u>Page 6 line 152:</u>
The SDR was first identified by aligning primer sequences of sex-linked markers (VVIB23 from Riaz *et al*., 2006; VSVV006, VVS007, VSVV09 and VSVV10 from Picq *et al*., 2014) to chromosome 2 of the Cabernet Sauvignon hap1 reference.

<u>**Fig. 2** caption, page 7 line 207:</u>
The filled, black triangles on this scale mark the position of the sex-linked genetic markers VVIB23 and VSVV010 (Riaz *et al*., 2006; Picq *et al*., 2014); the white-filled triangle represents the amplicon VSVV011 (Picq *et al*., 2014), which is not linked to the SDR.

<u>**Fig. 3** caption, page 9 line 246:</u>
The x-axis denotes the location on the Cabernet Sauvignon hap1 (H) SDR, and the two black triangles along this axis mark the position of the genetic markers VVIB23 and VSVV010 that are closely linked to the SDR (Riaz *et al*., 2006; Picq *et al*., 2014).

<u>Page 11 line 329:</u>
The 8 bp deletion in *VviINP1* as well as all other sex-linked polymorphisms in the SDR were confirmed by replicated bulk analysis of 120 individuals of the *Vv vinifera* x *V. arizonica* F1 population genotyped by whole genome resequencing using Illumina technology (**Supplementary Fig. 8**).

<u>In Supplementary Information, page 8 line 82:</u>
Supplementary Fig. 8: Bulk segregant analysis on 120 individuals from the population *Vv vinifera* F2-35 x *V. arizonica* b42-26.
a, The number of sex-linked SNPs per 100 kbp across Cabernet Sauvignon hap1 genome. SNPs were identified by aligning short-read sequencing data from 120 individuals of the F1 population *Vv vinifera* F2-35 x *V. arizonica* b42-26 split in four bulks, two composed of female individuals

(FF), called F1 and F2, two made of male individuals (MF), called M1 and M2. SNPs in homozygous state in both female pools (0/0/0/0 or 1/1/1/1) and in heterozygous state in both male pools (0/0/1/1) were considered fully sex-linked. Bulk segregant analysis confirmed the location of the sex-determining region on chromosome 2. Alignment of the short-read sequencing data confirm complete sex linkage of the 8 bp deletion in *VviINP1* (**b**), as well as the two non-synonymous SNPs in *VviYABBY3* (**c**) and SNPs identified in its promoter region (**d**).

The separate clustering of M and F haplotypes indicates that Vitis has sex chromosomes and they are ancestral in this genus. What about the YY genotypes? Are they viable? It will determine the stage of Vitis sex chromosome evolution.

To our knowledge, no YY genotype has been recorded and/or reported so far.

The authors have done excellent work and generated abundant genomic resources. Improved clarity and resolution in figures and text will enhance the quality and readability of this manuscript.

Minor changes:

Please add line numbers

We added line numbers to the manuscript.

It would be better to clearly state in Results and Methods that the genome sequences were generated by PacBio platform, not just SMRT DNA sequencing. Which platform? RSII, Sequel I, or Sequel II? Please add to the text.

We made clear in the Introduction and Results sections that genome assemblies are based on long reads generated with Pacbio sequencing technologies. Pacbio platforms are indicated in the Methods section.

Page 4 line 105:
All of these genomes are based on high-coverage, Pacific Biosciences long-read sequencing.

Page 5 line 138:
Each genome was based on Single Molecule Real Time (SMRT) DNA sequencing using the Pacific Biosciences (PacBio) technology and assembled *de novo* with FALCON-Unzip (Chin *et al*., 2016), which produces partially phased diploid genomes.

Page 20 line 658:
SMRTbell libraries were prepared as described in Minio *et al*. (2019a) and sequenced on a PacBio Sequel system using V3 chemistry, and on a PacBio RS II (Pacific Biosciences, CA,

USA) using P6-C4 chemistry for *Vv vinifera* cv. Merlot (DNA Technology Core Facility, University of California, Davis).

Page 1 affiliation 2: Delete extra "Dept."

We did the correction.

Page 1 line 9:
[2]Department of Ecology and Evolutionary Biology, University of California Irvine, Irvine, California, USA

Page 2 line 2: "… sex determination (SD) region…" - … sex determination region (SDR) …

Abbreviation was modified.

Page 2 line 16:
We studied the structure and evolution of the sex-determining region (SDR) in *Vitis* species to better understand the genes that contributed to the origin of dioecy in the genus and to understand how dioecy was lost in the domesticated grapevine.

Page 3 paragraph 2 line 1: "… economic importance of dioecy…" – dioecious crops are economically important, but most dioecious species are not crops.

The sentence was modified.

Page 3 line 39:
The taxonomic distribution of dioecy and economic importance of dioecious crops has made dioecy a focus of numerous evolutionary and genetic studies.

Page 3 paragraph 3 lines 3 - 7: All 70 species are dioecious, but one can not conclude dioecy is ancestral from that observation. You proved it in your study by analysis of M and F haplotypes. Perhaps change "indicating" to "hinting".

The sentence was modified.

Page 3 line 62:
The first is that dioecy is conserved; all ~70 *Vitis* species are dioecious (Moore and Wen, 2016), suggesting that dioecy has been conserved since before the origin of the genus.

Page 3 paragraph 4 lines 1 – 2: It shouldn't be the assumption that Vitis doesn't have sex chromosomes, and your results disproved it.

The sentence was removed.

Page 4 paragraph 2 lines 2 – 3 from bottom and Page 14 paragraph 4: If Vitis has no sex chromosomes or no recombination suppression in SDR, hermaphrodites will appear after every meiosis, and it wouldn't be hard to select during domestication. But your data disproved both assumptions. It is unlikely that hermaphrodites were derived from recombination, more likely from chromosomal rearrangement.

Strong linkage disequilibrium in the SDR was previously described in Picq *et al*., 2014. Our data confirmed this observation. Why recombination is suppressed in *Vitis* is not clear and we have highlighted this point in the discussion.

Page 4 line 85:
Polymorphisms within the region have high linkage disequilibrium in *Vv sylvestris*, suggesting low or no recombination between M and F haplotypes (Picq *et al*., 2014).

Page 11 line 332:
Finally, a peak of linkage disequilibrium (r^2 = 0.77) in the *VviINP1* genomic region (**Fig. 3c**) across 50 *Vv vinifera* (HF) accessions, suggesting a suppression of recombination at this locus.

Page 18 line 605:
Finally, we address one more question about recombination: if recombination can occur between F and M alleles, then what has kept the two haplotypes distinct for so long, given that dioecy has been maintained in the wild since the origin of the genus? This is an especially important question given the hypothesis that the rarity of dioecy among angiosperms is due to easy reversion to hermaphroditism (Käfer *et al*., 2017). The question's answer is obvious for *M. rotundifolia*, because 57% of the M haplotype is inverted relative to the F haplotype. This inversion is not only likely to slow recombination between the haplotypes (Lemaitre *et al*., 2009; Wang *et al*., 2012a), but it also helps further delineate the genes that function in sex determination (because recombination should be limited among the genes that influence sex determination and that therefore are within the inversion). Toward that end, it is interesting to note that *VviAPT3* is not within the inversion (**Fig. 2d**), further discounting that this gene represents the female-sterility mutation. But what slows recombination in wild *Vitis* spp., where we have yet to detect an inversion? Recombination may be deterred by differences in structure and length between M and F haplotypes, which are largely attributable to TE accumulation in intergenic space (He and Dooner, 2009). The SDR in grapevine is also relatively short and the close proximity of sex-determining genes may serve as an impediment to their recombination (Oberle 1938). These, the 50% probability of recombinants being successful hermaphrodites (**Fig. 6b**), and potential fitness costs associated with hermaphroditism in nature (Charlesworth and Charlesworth, 1978) may contribute to the conspicuous absence of hermaphroditic wild grapes.

In addition, all our results, including the distribution of M- and F-linked SNPs along the Cabernet Sauvignon H haplotype and the phylogenetic analysis, suggest a recombination event.

Sentences of interpretation of the results were added in order to help the reader.

Page 6 line 188:
Finally, H haplotypes were similar to F haplotypes within the first 60 kbp of the SDR but were more similar in structure to M haplotypes downstream of the SDR (**Fig. 2d**). This switch in higher structural similarity between H and F haplotypes in the 5' 60 kbp of the SDR to high similarity between H and M haplotypes for the remainder of the locus could be related to the origin of hermaphroditism in *Vv vinifera*.

Page 8 line 226:
This pattern of polymorphisms provides potential functional and evolutionary insights. Given that only H and F haplotypes support female function, the SDR region where M haplotypes differ from H and F haplotypes likely includes the female-sterility allele(s) in M haplotypes. Similarly, sequences where F haplotypes differ from M and H haplotypes likely include the genes that encode male function and contain the male-sterility allele(s) in F haplotypes. The observed distribution of sex-linked polymorphism thus provides preliminary insights into the origin of hermaphroditism in *Vv vinifera*, because H haplotypes are more similar to F haplotypes in the 5' region of the SDR but apparently more similar to M haplotypes in the 3' region (**Fig. 2d** and **3a**).

Page 10 line 282:
Third, these phylogenies are consistent with the observation of clusters of sex-specific polymorphisms (**Fig. 3a-b**), with F-like H haplotypes at the beginning of the region and M-like H haplotypes towards the end of the region **(Fig. 2)**. Finally, genes at the edges of the region do not cluster haplotypes by sex type, supporting the boundaries of the SDR delimited herein (**Fig. 3f**; **Supplementary Fig. 4**). Together, these observations support that the emergence of H haplotypes in domesticated *Vv vinifera* from dioecious wild relatives may have involved a recombination event near the aldolase and *TPP* genes (**Fig. 3e**).

Page 18 line 584:
We have yet to discuss an important question, which is the cause(s) of reversion to hermaphroditic flowers during domestication. It has been hypothesized that recombination between M and F haplotypes caused reversions (Picq *et al*., 2014; Henry *et al*., 2018; Zhou *et al*., 2019a). Consistent with this conjecture, several pieces of evidence support that H haplotypes arose from a recombination event, including their intermediate length and their similarity in structure and sequence to F haplotypes in the first portion of the SDR and to M haplotypes in the latter portion of the SDR (**Fig. 6a**). Based on our data, we could also localize the recombination event. Phylogenetic evidence supports that the recombination event occurred between the *aldolase* and *TPP* genes, and polymorphism information supports that it could have occurred within *TPP* gene, as the gene sequence contained both M- and F-linked non-synonymous SNPs (**Fig. 3a-f**; **Fig. 6b**). We note, however, that there is evidence that H haplotypes originated more than once in domesticated grapevine (Picq *et al*., 2014; Zhou *et al*., 2019a), suggesting that there could be different recombination breakpoints across H haplotypes. For that and others reasons, we caution that all of our inferences are based on our sample of twenty haplotypes; although we

have generated the largest collection of fully resolved SDR sequences in any plant to date, our sample may not be sufficient to fully elucidate the origins of hermaphroditism. Nonetheless, the point remains that any successful recombination event would need to separate male from female function, so that the vast majority of recombination events in the SDR are unlikely to lead to successful hermaphrodites.

Reviewer #2 (Remarks to the Author):

GENERAL COMMENTS

There are a few valuable bits of information in this ms, but it is severely marred by poor presentation, including lack of clear explanations (despite space being used for repeating things), poor English, absence of statistical test for claims, vagueness, and, worst of all, failure to put the results and conclusions in a context of previous understanding of sex determining regions, including those of plants. Many parts of the ms are undigested descriptions of results, and the reader is not told why they are being described or helped to understand what they tell us about the development or evolution of separate sexes in this plant, or more generally.

We would like to thank the reviewer for the comments above and for the extensive comments below. We have made substantial efforts to improve the manuscript in response to comments from this and the other reviewers. We agree that the suggested changes to the manuscript helped improve clarity and also helped highlight the impact of the discoveries we describe in the context of what is already known about the genetics of sex determination in grapes. Our study builds on the genetics and genomics work carried out to identify the sex-determining locus boundaries on chromosome 2, which we now describe and cite in this revised version of the manuscript.

Dissecting out the valuable information from this morass of description, it seems that the study may have done PacBio long-read sequencing that allowed them to determine X- and Y-linked haplotypes of a region of the grapevine chromosome 2 (of about 1 Mb between roughly 4.8 and 5.8 Mb of the previously published grapevine female assembly) in which the sex determining locus has been genetically mapped (the text uses the term “sex locus” instead of the correct term “sex determining locus”, and this should be corrected throughout).

The reviewer is correct. All 11 genomes were sequenced using PacBio long-read sequencing. We realized that it was only mentioned in the methods section, so we have added the information to the Introduction and Results sections.

<u>Page 4 line 105:</u>
All of these genomes are based on high-coverage, Pacific Biosciences long-read sequencing.

<u>Page 5 line 138:</u>
Each genome was based on Single Molecule Real Time (SMRT) DNA sequencing using the Pacific Biosciences (PacBio) technology and assembled *de novo* with FALCON-Unzip (Chin *et al*., 2016), which produces partially phased diploid genomes.

The sex-determining locus was previously genetically mapped on chromosome 2 (Dalbó *et al*., 2000; Riaz *et al*., 2006; Marguerit *et al*., 2009; Fechter *et al*., 2012; Battilana *et al*., 2013; Picq *et al*., 2014; Hyma *et al*., 2015; Zhou *et al*., 2017) and confirmed by the bulk segregant analysis we carried out as suggested by reviewer #1. Our study focused on this region in 11 genomes. We were able to identify the region across all 22 haplotypes using known linked genetic markers. We have expanded the description of how the location of the sex-determining locus was known prior to our study.

<u>Page 4 line 80:</u>
The third reason that *Vitis* is notable is because previous genetic and genomic studies have identified the approximate boundaries of the SDR (Dalbó *et al*., 2000; Riaz *et al*., 2006; Marguerit *et al*., 2009; Fechter *et al*., 2012; Battilana *et al*., 2013; Picq *et al*., 2014; Hyma *et al*., 2015; Zhou *et al*., 2017). In *Vitis* spp., the SDR maps genetically to ~150 kbp of chromosome 2 that contains between 15 and 20 genes (Fechter *et al*., 2012; Picq *et al*., 2014; Zhou *et al*., 2019b). Polymorphisms within the region have high linkage disequilibrium in *Vv sylvestris*, suggesting low or no recombination between M and F haplotypes (Picq *et al*., 2014). It was hypothesized that this region contains the recessive male-sterility and dominant female-sterility alleles predicted by the two-locus model, and their identification has been attempted by comparative gene expression analyses (Picq *et al*., 2014; Ramos *et al*., 2014). One such candidate, the adenine phosphoribosyltransferase gene *VviAPT3*, was expressed in the carpel primordial of male plants, suggesting a role in pistil abortion (Coito *et al*., 2017).

Until recently, a major limitation in the study of *Vitis* sex determination has been that the *Vv vinifera* reference genome represented only a partly assembled F haplotype (Jaillon *et al*., 2007). More recent work has partially resolved the sequence of H and F haplotypes, showing that they differ in the presence and absence of three genes (Zhou *et al*., 2019b). This work also annotated two previously unrecognized genes in the SDR, one of which is homologous to *INAPERTURE POLLEN 1* (*INP1*), which affects the deposition of pollen apertures in *Arabidopsis thaliana* (Dobritsa and Coerper, 2012). Yet, despite substantial progress our understanding of the SDR and the potential determinants of sex have been hampered by the absence of information from M haplotypes.

<u>Page 6 line 152:</u>
The SDR was first identified by aligning primer sequences of sex-linked markers (VVIB23 from Riaz *et al*., 2006; VSVV006, VVS007, VSVV09 and VSVV10 from Picq *et al*., 2014) to chromosome 2 of the Cabernet Sauvignon hap1 reference.

As requested we have changed “sex determination” to “sex-determining ” throughout the manuscript.

<u>Page 2 line 16:</u>

We studied the structure and evolution of the sex-determining region (SDR) in *Vitis* species to better understand the genes that contributed to the origin of dioecy in the genus and to understand how dioecy was lost in the domesticated grapevine.

Then, examining predicted genes within this region, the authors try to define the extent of a fully sex-linked region, and then to describe some candidate sex determining genes. All of these parts need revision, some of them including not just better organisation and explanation, but also better analyses. Space for better explanations can be made available by avoiding repetitions. Much of the Discussion section repeats results and approaches, not much more clearly than in the earlier parts, and it would be a good idea to use this section to tell us what has been understood about the questions of interest (see below).

We appreciate this comment, especially the point that the Results could provide more and clearer explanation. We have therefore tried to provide clearer conclusions and explanations in the Results, while also shortening the Discussion considerably. However, Results and Discussion still overlap to some extent, because we believe some overlap is necessary to explain the results in the context of the proposed model of sex determination.

I apologise if I have misunderstood some things, but, after a great deal of effort to read it, I found the ms quite confusing.

Overall, the methods used in this study are not well explained in the main text. It was not even made clear that the entire study is based on new PacBio sequencing. The study did not include any genetics, but relies on sequence data, which can indeed yield evidence that some variants are male- or female-specific (respectively suggesting Y or X linkage), provided that appropriate sample sizes of independent individuals are genotyped. The ms is vague about sample sizes.

As noted above, we have inserted “Pacific Biosciences” into the main text to make this clearer. The reviewer is correct that this study is based principally on sequencing data and not genetics. The genetics of sex determination in grapevine have been studied extensively previously. Our work builds on this previous work. As described above, in this revision we have tried to summarize more completely what had been done and how it enabled this study.

<u>Page 4 line 80:</u>
The third reason that *Vitis* is notable is because previous genetic and genomic studies have identified the approximate boundaries of the SDR (Dalbó *et al*., 2000; Riaz *et al*., 2006; Marguerit *et al*., 2009; Fechter *et al*., 2012; Battilana *et al*., 2013; Picq *et al*., 2014; Hyma *et al*., 2015; Zhou *et al*., 2017). In *Vitis* spp., the SDR maps genetically to ~150 kbp of chromosome 2 that contains between 15 and 20 genes (Fechter *et al*., 2012; Picq *et al*., 2014; Zhou *et al*., 2019b). Polymorphisms within the region have high linkage disequilibrium in *Vv sylvestris*, suggesting low or no recombination between M and F haplotypes (Picq *et al*., 2014). It was hypothesized that this region contains the recessive male-sterility and dominant female-sterility alleles predicted by the two-locus model, and their identification has been attempted by comparative gene expression analyses (Picq *et al*., 2014; Ramos *et al*., 2014). One such

candidate, the adenine phosphoribosyltransferase gene *VviAPT3*, was expressed in the carpel primordial of male plants, suggesting a role in pistil abortion (Coito *et al*., 2017).

Until recently, a major limitation in the study of *Vitis* sex determination has been that the *Vv vinifera* reference genome represented only a partly assembled F haplotype (Jaillon *et al*., 2007). More recent work has partially resolved the sequence of H and F haplotypes, showing that they differ in the presence and absence of three genes (Zhou *et al*., 2019b). This work also annotated two previously unrecognized genes in the SDR, one of which is homologous to *INAPERTURE POLLEN 1* (*INP1*), which affects the deposition of pollen apertures in *Arabidopsis thaliana* (Dobritsa and Coerper, 2012). Yet, despite substantial progress our understanding of the SDR and the potential determinants of sex have been hampered by the absence of information from M haplotypes.

Given the reviewers comments, we have also tried to emphasize the novelty of this paper. It not only resolves male haplotypes for the first time, it reports 9 new reference-level genomes. Based on these and previously available genomes, we analyze a total of 22 fully-resolved haplotypes of the sex-determining locus, 20 *Vitis* and 2 *Muscadinia*. To our knowledge, the ability to compare multiple haplotypes of each sex is unique, because there are few fully resolved sex-determining loci in plants. When they are resolved, it is often with gaps and single representations of Y-linked regions.

<u>Page 4 line 104:</u>
To fill this gap, we report **nine** new, phased diploid genomes of cultivated hermaphrodites and wild male and female grapes.

We have carefully included sample sizes in the text, especially in the Figure legends.

<u>Page 2 line 20:</u>
By resolving **twenty** *Vitis* SDR haplotypes, including the first generated for males, we were able to contrast male, female and hermaphrodite haplotype structure and to identify regions of sex-specific function.

<u>Page 5 line 132:</u>
To investigate the structure and evolution of the SDR in *Vitis* spp., we sequenced and assembled the complete genomes of **eight** *Vitis* accessions, including **three** hermaphrodite *Vv vinifera* cultivars (Merlot, Black Corinth seedless and Black Corinth seeded), **four** *Vv sylvestris* accessions (**two** females and **two** males), and **one** male *V. arizonica*. In addition, the genome of **one** male *Muscadinia rotundifolia* was constructed as a dioecious outgroup to *Vitis spp.* (**Fig. 1c**; Small, 1903; Moore, 1991; Mullins *et al*., 1992; Liu *et al*., 2016; Wen *et al*., 2018; Zecca *et al*., 2020).

<u>Page 6 line 171:</u>
For example, the **twelve** *Vitis* F haplotypes shared eight large deletions in comparison to the H haplotype of Cabernet Sauvignon, encompassing a total length of 117.4 kbp (**Fig. 2b**).

Page 6 line 175:
The **three** M haplotypes shared two large SVs relative to the H reference, including a 22.6 kbp insertion at position 4,802,134 and a 30 kbp deletion from positions 5,021,983 to 5,052,079.

Fig. 2 caption, page 7 line 201:
Schematic representations of the SDR in four of the **eleven** genomes analyzed for this study.

Page 8 line 219:
In total, 1,275 SNPs were shared by all **twelve** *Vitis* F haplotypes versus H and M haplotypes, and 270 SNPs were shared by all **three** M haplotypes versus F and H haplotypes.

Fig. 4 caption, page 12 line 348:
Alignment of the first 100 bp of **twenty** *VviINP1* coding sequences representing **twelve** F, **five** H and **three** M haplotypes along with **two** *M. rotundifolia INP1* coding sequences from F and M haplotypes.

In my opinion, the network analysis is inconclusive, and the conclusions speculative, and it should be removed (or published separately in a journal where it can be assessed by experts in such analyses).

We appreciate this comment, but we respectfully disagree. The network analyses constitute an important analysis of another novel feature of this study, which is gene expression gathered from stages of floral identify that have not been analyzed before. We realize that we may not have clearly established the novelty of these data in the previous version; we have tried to accentuate their novelty in the revision.

Page 13 line 397:
Previous studies have analyzed gene expression in the SDR and have compared flowers of different sexes (Ramos *et al*., 2014). However, these data were sampled from flowers at early developmental stages and may have missed the late steps of sex determination. Accordingly, we sampled flowers at three developmental stages: (i) during the early development of the reproductive structures, (ii) pre-bloom during pollen maturation, and (iii) at anthesis.

DETAILED COMMENTS
It is not at all remarkable among plants that Vitis does not have sex chromosomes, by which the authors mean heteromorphic sex chromosomes, but just a sex determining SD region or locus. At least half of dioecious plants that have been studied have such homomorphic "sex chromosomes", which might better be termed "fully sex-linked regions". It is well understood that these must have sex-specific haplotypes, and several recent studies have examined such regions in detail, for example in papaya (which has slight heteromorphism), asparagus, poplar and kiwifruit (with no heteromorphism detectable cytologically, although length differences have now been detected in the region in some of these). In all of these species, males are the heterozygous sex (male and female haplotypes are usually called X and Y, even though they are

not entire heteromorphic sex chromosomes; the notation MF in this ms could be used, if wanted, but ought to be explained if it is used). Vitis is not the only plant in which cultivated ‘strains’ are hermaphrodites. Other examples are papaya and probably spinach, Actinidia and strawberries. In Vitis and Spinacia, Westergaard reviews evidence that some hermaphrodites segregate with 3 staminate: 1 female ratios, suggesting Y-linkage (and an inviable YY genotype, suggesting that the Y-linked region may be ancient enough to have lost some functions), while others appear to have autosomal factors. It is possible that different hermaphrodites evolved independently through different genetic mechanisms, or perhaps that some hermaphrodites evolved long enough ago that the initial mutation of a Y-linked gene has been followed by an autosomal mutation that might improve female (or perhaps male) function. It would be good to mention that Westergaard’s 1958 suggested that the two gene model, including having an active Y-linked male-determiner, might apply to Vitis (as opposed to a system involving gene interactions that can result in a single gene system), and mentioned the hermaphrodites, along with reviewing other similar cases (see above). In this context, it is worth mention that the phrase “Genetic evidence for the two-locus model is not universal” is unsatisfactory. It implies that a different model, involving just a single gene, could lead to the evolution of dioecy. However, this overlooks the fact that mutations in two genes are necessary, but can result in just a single segregating locus, given suitable interactions that allow one of the mutations to fix in the species. This is clearly what has happened in Diospyros — the obeservation of a single gene does not mean that only a single mutation was required, and this can be seen in the paper by Akagi et al. that is cited by this ms, although the authors do not explain clearly the concept just mentioned.

In papaya, the hermaphrodites have long been known to carry “X” and “Y” chromosomes, in which the “Y” has lost the female-suppressor. This, and other similar situations, are reviewed in Westergaard’s 1958 review in Advances in Genetics, vol. 9, pages 217-281. The ms mentions none of this background. The ms is, in general, very weak in describing the state of knowledge prior to the new work. Previous understanding of the sex determining region in cultivated grapevines is poorly explained, but (in my edited wording, which needs to be amplified in the region indicated by []), it seems that “ The Vv vinifera fully sex-linked region has been delimited to a ~200 kbp region of chromosome 2 (Fechter et al., 2012; Picq et al., 2014; Zhou et al., 2019b), and current evidence [WHOSE NATURE SHOULD BE BRIEFLY OUTLINED HERE so that readers can see what is new from the results in this new study] supports the two-locus model. One new result would be if the sex-determining genes were identified, which they were not previously, although the 15 to 20 genes within the region are candidate SD genes (Dalb. et al., 2000; Riaz et al., 2006; Marguerit et al., 2009; Fechter et al., 2012; Battilana et al., 2013; Hyma et al.,2015; Zhou et al., 2017)”.

We are grateful for these comments. All of the pertinent work in grapevine was cited previously for interested readers, but on rereading, we agree that we should have: i) done a better job reporting the state of knowledge in grapevine for the general reader, ii) shown how our work fits into that context and ii) made the specific goals of this manuscript more explicit. We have revised the manuscript accordingly.

<u>Page 4 line 80:</u>

The third reason that *Vitis* is notable is because previous genetic and genomic studies have identified the approximate boundaries of the SDR (Dalbó *et al*., 2000; Riaz *et al*., 2006;

Marguerit *et al*., 2009; Fechter *et al*., 2012; Battilana *et al*., 2013; Picq *et al*., 2014; Hyma *et al*., 2015; Zhou *et al*., 2017). In *Vitis* spp., the SDR maps genetically to ~150 kbp of chromosome 2 that contains between 15 and 20 genes (Fechter *et al*., 2012; Picq *et al*., 2014; Zhou *et al*., 2019b). Polymorphisms within the region have high linkage disequilibrium in *Vv sylvestris*, suggesting low or no recombination between M and F haplotypes (Picq *et al*., 2014). It was hypothesized that this region contains the recessive male-sterility and dominant female-sterility alleles predicted by the two-locus model, and their identification has been attempted by comparative gene expression analyses (Picq *et al*., 2014; Ramos *et al*., 2014). One such candidate, the adenine phosphoribosyltransferase gene *VviAPT3*, was expressed in the carpel primordial of male plants, suggesting a role in pistil abortion (Coito *et al*., 2017).

Until recently, a major limitation in the study of *Vitis* sex determination has been that the *Vv vinifera* reference genome represented only a partly assembled F haplotype (Jaillon *et al*., 2007). More recent work has partially resolved the sequence of H and F haplotypes, showing that they differ in the presence and absence of three genes (Zhou *et al*., 2019b). This work also annotated two previously unrecognized genes in the SDR, one of which is homologous to *INAPERTURE POLLEN 1* (*INP1*), which affects the deposition of pollen apertures in *Arabidopsis thaliana* (Dobritsa and Coerper, 2012). Yet, despite substantial progress our understanding of the SDR and the potential determinants of sex have been hampered by the absence of information from M haplotypes.

<u>Page 4 line 109:</u>
With these extensive new sequence and expression data, we compared the F, H, and M haplotypes to better define the SDR, identify candidate sex-determining genes, and reconstruct a key step in the domestication of *Vv vinifera*, namely the recombination event that resulted in the reversion to hermaphroditism observed in domesticates.

If the aim of the study is to test the two-gene model better for Vitis, using the latest sequencing and assembly methods that have now become available, this should be stated, along with stating the approach to be used.

As noted above, it was not our intent to test the two-locus model. Our intent was to gain a better understanding into organization of the sex-determining locus by resolving multiple haplotypes of the sex-determining locus, including (for the first time) data from males. We also sought to use these data to gain clues into the two sex-determining regions and that causes of reversion in cultivated grapes. We have tried to make this motivation clearer because it seems that our lack of clarity was the basis for many of the reviewer’s comments.

In this context, it is worth emphasizing again that complete genomic resolution of sex-determining regions is rare in plants, having been achieved in only a handful of plant species. Here we have resolved this region in 11 different genomes, including the first male; this is not a trivial feat.

I think the authors believe that this can be done by sequencing and identifying candidate genes. However, of course, if there is a completely non-recombining region containing as many as 15 genes, it is unlikely to be possible to discover which of them is involved, because sequence variants may be present in several of them, some of which lead to loss of one sex functional or the other, while other fully Y-linked variants may simply be mutations (perhaps without functional significance) that occurred in the Y-linked since it stopped recombining with the X-linked homologous region. If the fully sex-linked region includes genes with functions that strongly suggest their involvement in sex determination, then this is certainly an advance (though their functional involvement needs to be tested, for example in transgenic experiments, which are clearly beyond the scope of this study). This study did identify a candidate male-sterility mutation (the inaperturate pollen gene), but its expression is higher n females than males, making the evidence that it is an actual SD gene less clear.

Overall, therefore, the sex determining genes have not yet been definitively identified, and it is not yet possible to say whether two genes in the region identified were indeed involved in the evolution of separate sexes.

We agree with all of these comments! We were, in fact, very careful not to claim that we had found a sex-determining gene; such a claim requires a test of function, which is beyond the scope of this paper given that such a test is several years down the road for this non-model perennial plant. We had hoped that we were clear that our efforts were: i) to compare M, F and H haplotypes, ii) to provide insights into the content of the region and iii) to use differences in content among haplotypes to whittle down the number of candidate sex-determining genes.

<u>Page 4 line 109:</u>
With these extensive new sequence and expression data, we compared the F, H, and M haplotypes to better define the SDR, identify candidate sex-determining genes, and reconstruct a key step in the domestication of *Vv vinifera*, namely the recombination event that resulted in the reversion to hermaphroditism observed in domesticates.

It would be helpful to understand what is known about the time when dioecy evolved in the genus Visit, or an ancestor of the species studied. Clearly, a first step is to find out whether there really is a completely sex-linked region, and to estimate its age. Then the genes in the region can be identified and tested. If dioecy is ancient, then the idea that genes in the sex-linked region may have evolved changes since the SD mutations/system became established might be plausible (see also below). I have divided my comments into sections dealing with the different questions, in some kind of logical order.

As mentioned in the manuscript, dioecy extends beyond *Vitis* to the genus *Muscadinia*, one sample of which was included in the manuscript. We cited previous work that these two genera diverged ~50 million years ago, but one reviewer asked us to modify this date based on disagreements in the literature. In addition to modifying the data of divergence between genera, we now report a dS analysis for the *VviINP1* gene.

<u>Page 11 line 304:</u>

The average synonymous distance (dS) between all pairs of F and M alleles of *INP1* was 0.0275 substitutions per base. Assuming a generation time of 3 years and a nucleotide substitution rate of $2.5x10^{-9}$ substitutions per base per year (Zhou *et al*., 2017), we infer that M and F alleles diverged ~16.5 million years ago (mya), a value within the range of uncertainty of the estimated split time between *Vitis* and *Muscadinia* (Wan *et al*., 2013).

QUESTION 1: Is there a completely non-recombining region?
The Methods section does not give adequate detail about important aspects such as the phasing. The ms does not clearly describe the nature of the sequencing approach used, other than "Single Molecule Real Time (SMRT) DNA sequencing" (which I think means PacBio), and the statement that the results were phased (e.g. parameter values used), without any information about the sequencing quality, how the results were validated or how the boundaries of the putative fully sex-linked region (or SD region) were determined, and whether there is a clear cut-off at each end. This information is important for assessing whether the complete region has been reliably identified, and whether it is truly non-recombining, or recombines rarely (as suggested when discussing the origin of hermaphrodites in domesticated cultivars).

A few points: First, we again apologize that we did not explicit state Pacific Biosciences in the text. SMRT-reads are short-hand for PacBio sequencing in the world of genomics, but it should have been clearer. Second, information about quality was included in the first paragraph of the Results, in the Materials and Methods and in the **Supplementary Table 1**. Third, the pertinent phasing information was mentioned in the Results and in the Materials and Methods. Those familiar with the method would understand how phasing was done, but we now include a brief phrase about the fact that the assembly method is the state of the art for phasing.

Page 5 line 138:
Each genome was based on Single Molecule Real Time (SMRT) DNA sequencing using the Pacific Biosciences (PacBio) technology and assembled *de novo* with FALCON-Unzip (Chin *et al*., 2016), which produces partially phased diploid genomes.

Page 21 line 690:
Haplotype phasing was carried out using FALCON-Unzip. FALCON-Unzip was designed to combine single-nucleotide polymorphisms and structural variants to separate long sequencing reads based on their haplotype, which are then assembled into separate contigs (Chin *et al*., 2016). FALCON-Unzip was shown to successfully phase heterozygous regions in plants, including grapes (Chin *et al*., 2016; Minio *et al*., 2019b).

Finally, as we stated previously in the text, the sex-determining region was defined by previous genetic studies. We used the markers from that study to delineate the sex-determining locus on genomic sequences. We now also include a supplementary figure (**Supplementary Fig. 2**) showing that associations with gender disappear rapidly beyond the edges of the sex-determining region, reinforcing the notion that we are indeed studying the sex-determining region. Location of the sex-determining locus was confirmed on chromosome 2 by bulked segregant analysis. In the revised manuscript, we added sequencing data of 120 progeny of the interspecific F1 used in

this study, divided in four bulks based on sex type. Bulk segregant analysis confirmed the location of the sex-determining locus on chromosome 2 and confirmed the complete sex-linkage of the polymorphisms we identified by comparing the 20 *Vitis* haplotypes, including the candidate mutations in *VviYABBY3* and *VviINP1* (**Supplementary Fig. 8**).

Page 6 line 152:
The SDR was first identified by aligning primer sequences of sex-linked markers (VVIB23 from Riaz *et al*., 2006; VSVV006, VVS007, VSVV09 and VSVV10 from Picq *et al*., 2014) to chromosome 2 of the Cabernet Sauvignon hap1 reference.

Supplementary Information, page 8 line 82:
Supplementary Fig. 8: Bulk segregant analysis on 120 individuals from the population *Vv vinifera* F2-35 x *V. arizonica* b42-26.
a, The number of sex-linked SNPs per 100 kbp across Cabernet Sauvignon hap1 genome. SNPs were identified by aligning short-read sequencing data from 120 individuals of the F1 population *Vv vinifera* F2-35 x *V. arizonica* b42-26 split in four bulks, two composed of female individuals (FF), called F1 and F2, two made of male individuals (MF), called M1 and M2. SNPs in homozygous state in both female pools (0/0/0/0 or 1/1/1/1) and in heterozygous state in both male pools (0/0/1/1) were considered fully sex-linked. Bulk segregant analysis confirmed the location of the sex-determining region on chromosome 2. Alignment of the short-read sequencing data confirm complete sex linkage of the 8 bp deletion in *VviINP1* (**b**), as well as the two non-synonymous SNPs in *VviYABBY3* (**c**) and SNPs identified in its promoter region (**d**).

The sample sizes are also not entirely clear. The text mentions 22 haplotypes of the SD region, but 9 accessions were sequenced (presumably 18 haplotypes). Maybe the number 22 includes the outgroup species that was sequenced (see also below)? This sample size is rather small for establishing statistically significant associations between the sex and sequence variants, especially in cultivated material, where it is possible that one cultivar is descended from another, so that associations are not equivalent to those based on studying a sample from a single natural population. Even without common ancestry of this kind, a bottleneck during domestication will produce elevated linkage disequilibrium, so that associations may be false positives. The text should give some consideration to these points. Ideally, any potentially interesting associations should be validated using a natural population sample, or (in a cultivated species, a set of cultivars derived independently from the progenitor). The ms does describe tests in 2 segregating families, which do support sex linkage, but cannot demonstrate that rare recombination is absent. If the ideal
sample cannot be studied, or the wild progenitor is now extinct, this issue should be discussed.

Thank you for these comments. To clarify the sampling size, we have restated the number of genomes (10 *Vitis* + 1 *Muscadinia*) and haplotypes (20 *Vitis* + 2 *Muscadinia*) throughout the manuscript, including the Figure Legends, to make analyses more transparent (see above).

We ask the reviewer to recognize that we are not trying associate polymorphisms statistically. We are describing fixed differences among resolved male, female and hermaphrodite haplotypes; that is, all our analyses rely only on *perfect* associations with sex type. Given that there are few

fully resolved sex regions for other plant species, we think that our sample size is impressive. However, we agree that we should include caveats related to sample size and have included a short statement to that effect in the Discussion.

Page 18 line 597:
For that and others reasons, we caution that all of our inferences are based on our sample of twenty haplotypes; although we have generated the largest collection of fully resolved SDR sequences in any plant to date, our sample may not be sufficient to fully elucidate the origins of hermaphroditism.

The purpose of the neighbor-joining phylogenetic trees for each gene is not clear, but it presumably related to this question, since, if the genes are fully sex linked, the phylogenies should be very similar. In Figure 3, one can see that several genes (from the TPP gene to the left of the candidate male-sterility gene with the stop codon causing inaperturate pollen, INP1, to the 7th gene on its right, VviFSEX) yield trees in which males and hermaphrodites form a single cluster (similar to the papaya situation in which hermaphrodites are probably mutant males whose Y-linked region has lost the female suppressor), while the other genes, at the two ends of the region analysed, do not evidently agree with the statement above. This appears to suggest that those 'flanking' genes might be partially, not fully, sex-linked, although they might show associations (linkage disequilibrium) with the fully sex-linked region. It would be good to integrate information so that readers know which regions fall into each of these categories. Perhaps the authors are trying to say that the sequences allow them to infer this, but this is not clearly communicated.

The purpose of the phylogenies was similar to what the reviewer describes, but the reviewer's comments suggest that there was some confusion over what they demonstrate. We have included additional sentences and explanations to try to help guide the reader towards their purpose and interpretation, particularly with respect to the fact that the reference hermaphrodite allele represents an M-F recombinant and that these phylogenies are resolving features of the recombination event.

Page 8 line 226:
This pattern of polymorphisms provides potential functional and evolutionary insights. Given that only H and F haplotypes support female function, the SDR region where M haplotypes differ from H and F haplotypes likely includes the female-sterility allele(s) in M haplotypes. Similarly, sequences where F haplotypes differ from M and H haplotypes likely include the genes that encode male function and contain the male-sterility allele(s) in F haplotypes. The observed distribution of sex-linked polymorphism thus provides preliminary insights into the origin of hermaphroditism in *Vv vinifera*, because H haplotypes are more similar to F haplotypes in the 5' region of the SDR but apparently more similar to M haplotypes in the 3' region (**Fig. 2d** and **3a**).

Page 10 line 271:
To better understand the history of the SDR and identify male-sterility and female-sterility candidates, we constructed phylogenies from *Vitis* sequences for each gene in the region (**Fig. 3f**; **Supplementary Fig. 4**), yielding four observations. First, the alleles tended to cluster by sex

type across most of the SDR (**Fig. 3f**; **Supplementary Fig. 4**) in a manner consistent with the sex-linked polymorphisms discovered. For example, all of the *VviYABBY3* and aldolase alleles formed clades that separated M from F and H orthologs (**Fig. 3f**). Second, the pattern of clustering varied along the SDR. The phylogenies of four genes at the beginning of the region (from *VviYABBY3* to the aldolase gene) clustered M sequences apart from F and H sequences. This pattern switched from *TPP* onward; for *TPP*, *VviINP1*, *exostosin*, *KASIII*, *PLATZ*, the three *FMO*, the hypothetical protein gene (*VviFSEX*), and *VviAPT3,* F sequences clustered apart from M and H alleles (**Fig. 3f**). Third, these phylogenies are consistent with the observation of clusters of sex-specific polymorphisms (**Fig. 3a-b**), with F-like H haplotypes at the beginning of the region and M-like H haplotypes towards the end of the region **(Fig. 2)**. Finally, genes at the edges of the region do not cluster haplotypes by sex type, supporting the boundaries of the SDR delimited herein (**Fig. 3f**; **Supplementary Fig. 4**). Together, these observations support that the emergence of H haplotypes in domesticated *Vv vinifera* from dioecious wild relatives may have involved a recombination event near the aldolase and *TPP* genes (**Fig. 3e**).

Another odd decision concerning the tree analysis is not to include the outgroup species. The trees are therefore unrooted, and rooted trees would have been preferable, with bootstrap support values. If dioecy pre-dates the split between Vitis and Muscadinia, they could well have the same clusters of Y-linked sequences, as has been found in other cases of genetic polymorphisms that pre-date a species split. The ms indeed mentions that "the same 8 bp deletion was also found in the female M. rotundifolia, [so] this F-specific INDEL likely occurred before Vitis and Muscadinia diverged". As both species are dioecious, it is also likely that the female suppressor may also be present in the latter, and the tree in Fig. 1 suggests that these are not too highly diverged to try this analysis (it would be good to mention the synonymous site divergence between a sample of genes in these species).

Please note that the phylogenies in **Fig. 3f** are illustrative, to show how M-H cluster for some gene phylogenies (hence defining the male-linked region of the sex-determining locus) and F-H cluster for other phylogenies (hence defining the female-linked region of the SD locus). Note that haplotypes do not cluster by sex for the extreme 5' and 3' loci of our study, suggesting we have captured all of the sex-linked loci. We included phylogenies with bootstraps in the **Supplementary Fig. 4**. We have also modified **Fig. 4b** to include outgroups and divergence time estimates. Bootstraps for **Fig. 4b** are included, as before. We have also revised the main text to make the purpose of these phylogenies clearer.

Page 10 line 284:
Finally, genes at the edges of the region do not cluster haplotypes by sex type, supporting the boundaries of the SDR delimited herein (**Fig. 3f**; **Supplementary Fig. 4**).

Page 11 line 302:
Phylogenetic analysis of the *INP1* protein clustered *Vitis* and *Muscadini*a orthologs by sex, confirming the sex-specificity of *INP1* alleles (**Fig. 4b**; **Supplementary Fig. 5**). The average synonymous distance (dS) between all pairs of F and M alleles of *INP1* was 0.0275 substitutions per base. Assuming a generation time of 3 years and a nucleotide substitution rate of 2.5×10^{-9} substitutions per base per year (Zhou *et al*., 2017), we infer that M and F alleles diverged ~16.5

million years ago (mya), a value within the range of uncertainty of the estimated split time between *Vitis* and *Muscadinia* (Wan *et al*., 2013).

It is potentially of interest that the Y-linked region is inverted in the outgroup species, M. rotundifolia, as the two-gene model for the evolution of separate sexes suggests that fully Y- and Y-linked regions evolve in 3 steps, first, the spread male- and female-sterility mutations at two separate, but closely-linked sites on a chromosome, and then the evolution of closer linkage (this will prevent rare X-Y recombination events, which will generate hermaphrodites and, even worse, neuter phenotypes, which are sterile). The observed inversion could reflect a rearrangement like that proposes as step 3 (indeed the ms suggests that X-Y recombination may have allowed reversion to hermaphroditism in Vitis). This inversion would presumably have had to happen after the split from Vitis, and appears not to have occurred in Vitis (unless inverted arrangements in Vitis have not become fixed, and have not been detected in the limited sampling of this species). The observation of an inversion that is limited rather precisely to the sex-determining region is another potentially interesting result of this sequencing, but the ms does not really discuss what it tells us, or might tell us, given further study.

We dedicated the last paragraph of the Discussion to this point.

Page 18 line 605:
Finally, we address one more question about recombination: if recombination can occur between F and M alleles, then what has kept the two haplotypes distinct for so long, given that dioecy has been maintained in the wild since the origin of the genus? This is an especially important question given the hypothesis that the rarity of dioecy among angiosperms is due to easy reversion to hermaphroditism (Käfer *et al*., 2017). The question's answer is obvious for *M. rotundifolia*, because 57% of the M haplotype is inverted relative to the F haplotype. This inversion is not only likely to slow recombination between the haplotypes (Lemaitre *et al*., 2009; Wang *et al*., 2012a), but it also helps further delineate the genes that function in sex determination (because recombination should be limited among the genes that influence sex determination and that therefore are within the inversion). Toward that end, it is interesting to note that *VviAPT3* is not within the inversion (**Fig. 2d**), further discounting that this gene represents the female-sterility mutation. But what slows recombination in wild *Vitis* spp., where we have yet to detect an inversion? Recombination may be deterred by differences in structure and length between M and F haplotypes, which are largely attributable to TE accumulation in intergenic space (He and Dooner, 2009). The SDR in grapevine is also relatively short and the close proximity of sex-determining genes may serve as an impediment to their recombination (Oberle 1938). These, the 50% probability of recombinants being successful hermaphrodites (**Fig. 6b**), and potential fitness costs associated with hermaphroditism in nature (Charlesworth and Charlesworth, 1978) may contribute to the conspicuous absence of hermaphroditic wild grapes.

The ms mentions that the Vitis SD haplotypes differ in length (though it is not very clear which regions differ and what the differences are — for example, are the Y introns longer than the X versions, or are transposable elements detected in non-coding regions (Fig. 1 shows that Y

haplotypes seem to be consistently longer than their X counterparts from the same species, and that inter-genic regions are expanded, although the sample sizes are small)? Some detail might be helpful. The ms also suggests that such length differences may contribute to suppressing recombination, but does not consider which is the cause and which the effect. An inversion is clear, because one can infer the ancestral stare, and it does indeed have the potential to suppress recombination, especially if only a small region is inverted.

We recognize that we cannot disentangle cause from effect, but now mention that point explicitly in the discussion. We also provide additional information about the content of the intergenic regions that differ in length between M and F haplotypes.

Page 6 line 171:
For example, the twelve *Vitis* F haplotypes shared eight large deletions in comparison to the H haplotype of Cabernet Sauvignon, encompassing a total length of 117.4 kbp (**Fig. 2b**). It is worthy to note that these F-linked SVs were mainly composed of transposable elements (62.9%), including LTR, Gypsy, Copia and MuDR elements.

Page 19 line 616:
But what slows recombination in wild *Vitis* spp., where we have yet to detect an inversion? Recombination may be deterred by differences in structure and length between M and F haplotypes, which are largely attributable to TE accumulation in intergenic space (He and Dooner, 2009). The SDR in grapevine is also relatively short and the close proximity of sex-determining genes may serve as an impediment to their recombination (Oberle 1938). These, the 50% probability of recombinants being successful hermaphrodites (**Fig. 6b**), and potential fitness costs associated with hermaphroditism in nature (Charlesworth and Charlesworth, 1978) may contribute to the conspicuous absence of hermaphroditic wild grapes.

QUESTION 2: It is a separate question to ask "Can the sex-determining genes be identified?"

Rather than looking at trees, it might be better simply to show which sequence variants are restricted to males, suggesting Y-linkage (although, as explained above the sample sizes are very small for any strong conclusions). Such variants should show the genotype configurations expected under the form of sex system seen, for example XY regions should include male-specific variants, indicating Y-linkage, and one can test (given an adequate sample) what proportion of variants in a window of given size show the expected patters with all males heterozygous and all females homozygous for the X-linked allele. Given the phasing analysis, the trees may be a redundant analysis.

We did not look only at trees, because we plotted male- and female-specific variants in **Fig. 3** for nucleotides, non-synonymous sites, and transcription binding factor motifs. By doing so, we illustrate regions that appear to be M-specific, given our sample. We agree, that the trees are slightly redundant to the plots, but we believe that they convey important information as well. Hopefully our explanatory text has helped here.

Page 8 line 214:

We used our sequence alignments to Cabernet Sauvignon to identify SNPs that associate perfectly with sex among *Vitis* spp.. These were polymorphisms fixed in one sex haplotype versus the other two sex haplotypes. All of the F- and M-associated polymorphisms were found from positions 4,801,876 to 5,061,548 on chromosome 2 of the Cabernet Sauvignon hap1 reference (**Fig. 3a**; **Supplementary Table 2**; **Supplementary Fig. 2**), which further confirms and delimits the SDR (Fechter *et al*., 2012; Picq *et al*., 2014). In total, 1,275 SNPs were shared by all twelve *Vitis* F haplotypes versus H and M haplotypes, and 270 SNPs were shared by all three M haplotypes versus F and H haplotypes.

<u>Page 10 line 257:</u>
Next, potential male-sterility and female-sterility mutations were identified among non-synonymous mutations that segregated F from M and H haplotypes and segregated M from F and H, respectively (**Fig. 3b**). In total, 89 non-synonymous F-specific SNPs were detected in ten genes (**Supplementary Table 2**). These included one in a gene encoding a trehalose-6-phosphate phosphatase (*TPP*), one in *VviINP1*, seven in an exostosin-coding gene, three in a 3-ketoacyl-acyl carrier protein synthase III gene (*KASIII*), seven in a PLATZ transcription factor (TF)-coding gene (*PLATZ*), eighteen in the first *FMO* gene, twenty-six in the second *FMO*, eleven in the third *FMO*, eleven in the hypothetical protein *VviFSEX*, and four in *VviAPT3*. Three of these SNPs introduce a premature stop codon in the first two of four *FMO* genes (**Fig. 3e**). In contrast, we found only six non-synonymous M-linked SNPs: two in the YABBY TF-coding gene *VviYABBY3* (Zhang *et al*., 2019), two in an aldolase-coding gene, one in *TPP*, and one in the third *FMO*. These non-synonymous M-linked SNPs represent potential female-sterility mutations.

<u>Page 10 line 292:</u>
In addition to SNPs, we identified 156 and 25 small INDELs (≤ 50 bp) shared by all F and M haplotypes, respectively, relative to the Cabernet Sauvignon H reference.

<u>Page 12 line 365:</u>
In order to assess the potential impact of sex-linked SNPs and INDELs on the regulation of the genes comprised in the SDR, we searched for sex-linked TF-binding sites within 3 kbp regions upstream of transcription start sites (**Fig. 3d**; **Supplementary Fig. 9**; **Supplementary Table 5**). M-linked TF-binding motifs were identified upstream of the genes encoding the PPR-containing protein, VviYABBY3, the aldolase, KASIII, FMOs and the hypothetical protein (VviFSEX). Two of these motifs were associated with flowering and flower development, including binding sites for SHORT VEGETATIVE PHASE (SVP), which is involved in the control of flowering time by temperature (Lee *et al*., 2007), and BES1-INTERACTING MYC-LIKE1 (BIM1), a brassinosteroid-signaling component involved in *A. thaliana* male fertility (Xing *et al*., 2013). Similarly, we identified TF-binding motifs upstream of *VviINP1*, *exostosin*, *KASIII*, *PLATZ*, *FMOs*, *VviFSEX*, *WRKY*, and *VviAPT3* that were unique to F haplotypes (**Fig. 3d**; **Supplementary Table 5**).

It is stated that the haplotypes tended to cluster by sex, but no test is described. It is not clear why this test was not done for the entire fully sex-linked region, as this would be best tested using all variants in the region. It is also not explained why the alignments were concatenated, as one

would expect each gene to be represented within a long read, assuming that long-read sequencing was done (see above).

As stated in the text, we looked at polymorphisms that associated perfectly with sex. No statistical test was described because none was applied. Simply put, this is taking the first large sample of fully resolved sex haplotypes from any plant species and looking for which variants associate perfectly with sex. Clearly there are limits to this approach due to sample size, etc.. We have modified the text to highlight the caveats, given the reviewer's comments.

Page 8 line 214:
We used our sequence alignments to Cabernet Sauvignon to identify SNPs that associate perfectly with sex among *Vitis* spp.. These were polymorphisms fixed in one sex haplotype versus the other two sex haplotypes.

Fig. 3 caption, page 9 line 243:
Only SNPs strictly (100%) linked to one sex type were retained.

First, however, it is also not completely clear whether each individual's genome sequence was independently assembled, meaning that each sex's SD region was assembled separately, or whether the Cabernet Sauvignon reference assembly was used. It was used for some analyses, and it is not entirely clear which ones. It is also not clear which sex was used, as this cultivar is stated to have females and hermaphrodites. If a female reference genome was used to map the new reads, or help assemble them, is there any risk that sequences specific to the Y-linked region could have failed to be detected? This should be discussed clearly.

All genomes were assembled *de novo* from long PacBio reads. Each genome was independently phased and each sex-determining region independently manually annotated. We did not put the phrase "*de novo*" in the text, preferring to mention the methods that were used, which clearly point to *de novo* assembly. We have inserted the phrase, however, for those unfamiliar with the methods.

Page 5 line 138:
Each genome was based on Single Molecule Real Time (SMRT) DNA sequencing using the Pacific Biosciences (PacBio) technology and assembled *de novo* with FALCON-Unzip (Chin *et al*., 2016), which produces partially phased diploid genomes.

Before tackling question 2, it would be helpful to separate it into distinct tests, as follows
(i) Examining just males and females, are some variants male-specific, suggesting Y linkage? If so, can one identify candidate male-sterility mutations in the X haplotype, and female-suppressing ones in the Y haplotype? Only after this is clear should the hermaphrodites be examined, as explained next.
(ii) For sequences within the fully sex-linked region, are hermaphrodites' sequences more similar to those of males' Y alleles, or to X-linked alleles? If the former, this suggests that (as in the

papaya Yh chromosome) hermaphrodites have a modified Y that has lost the female suppressing factor carried by "true" Ys.

(iii) The result of (ii) is important, because the comparison between Y and Yh regions can help test candidate genes — specifically, if Yh has lost the female suppressing factor, or has a non-functional copy, and this occurred recently (as the text suggests), this could be used to test whether a candidate gene is plausible. Moreover, it would also increase the sample size of Y haplotypes, because, apart from this mutation, the rest of this region should be a normal Y that can be compared with X haplotypes.

We believe our approach is more powerful than that which is suggested here, for the following reason. First, it has been suggested that hermaphrodites are recombinants between M and F, and our full haplotypes are consistent with that suggestion. Second, given the possibility that H haplotypes are part M-like and part F-like, this provides a powerful method to identify the regions that confer male and female function. This is exactly what we do in **Fig. 3a**.

We are sorry if this was unclear. We have tried to make this more explicit in the revision, in the hope it becomes clearer to this reviewer. We also hope that these changes make the work more accessible to a broader audience.

Page 8 line 219:
In total, 1,275 SNPs were shared by all twelve *Vitis* F haplotypes versus H and M haplotypes, and 270 SNPs were shared by all three M haplotypes versus F and H haplotypes.

Page 8 line 224:
Interestingly, M-linked SNPs were more dense in the first 8 kbp of the SDR (176 SNPs, 4,801,876 to 4,809,592) and the first F-linked SNP was ~40 kbp downstream (4,842,196; **Fig. 3a**). This pattern of polymorphisms provides potential functional and evolutionary insights. Given that only H and F haplotypes support female function, the SDR region where M haplotypes differ from H and F haplotypes likely includes the female-sterility allele(s) in M haplotypes. Similarly, sequences where F haplotypes differ from M and H haplotypes likely include the genes that encode male function and contain the male-sterility allele(s) in F haplotypes. The observed distribution of sex-linked polymorphism thus provides preliminary insights into the origin of hermaphroditism in *Vv vinifera*, because H haplotypes are more similar to F haplotypes in the 5' region of the SDR but apparently more similar to M haplotypes in the 3' region (**Fig. 2d** and **3a**).

Page 10 line 282:
Third, these phylogenies are consistent with the observation of clusters of sex-specific polymorphisms (**Fig. 3a-b**), with F-like H haplotypes at the beginning of the region and M-like H haplotypes towards the end of the region **(Fig. 2)**.

Page 16 line 488:
M-linked polymorphisms occur in the 5' region spanning from the promoter region of the PPR-containing protein through to *TPP*, and F-linked polymorphisms span *TPP* through *VviAPT3* (**Fig. 6a**). These distinct regions provide an opportunity to disentangle the genetic determinants of sex trait. For example, under the two-locus model of the origin of dioecy, the dominant

female-sterility allele is expected to be unique to M haplotypes, and therefore located in the region where M haplotypes differ from F and H haplotypes.

Instead of a clear analysis, with clear steps, the ms confusingly describes comparisons between all 3 haplotypes, making it very difficult to understand what the results tell us. It is nevertheless hopeful that, among the plethora of sequence variants (SNPs and indels) described, some are candidates for involvement in sex determination.

Specifically, a frame-shift that results in production of a truncated protein may represent the male-sterility mutation that created females from a presumably hermaphroditic ancestor (the ms also fails to mention what is known about the likely ancestral state, and it mentions that dioecy has been maintained for a long time, without explicit details; these should be added, including synonymous site divergence values). However, this gene was found to be transcribed at a higher level in females than in males, making its involvement unclear. Presumably this is based on estimates of transcript abundances, and no details are provided about how the two different transcripts were measured (do they behave the same in the assay that was used, or does the stop codon affect the transcripts, or their abundance?). Details of the numbers of replicates, and the methods, are also missing or vague.

The information about gene expression (sample sizes and handling of reads) is provided in the Materials and Methods section. We agree that the up-regulation of *VviINP1* is surprising and inserted text that states so in the manuscript. An upregulation in the expression of non-functional alleles has been previously reported in CRISPR mutants (Smits *et al*., 2019), work we cite when discussing the phenomenon. As noted above, we have included information about dS for the *INP1* gene.

<u>Page 13 line 409:</u>
For example – and to our surprise – *VviINP1* was significantly more highly expressed in pre- and post-bloom female flowers compared to male and hermaphroditic flowers (adjusted P value ≤ 0.05).

<u>Page 17 line 543:</u>
Why *VviINP1* was more highly expressed in female flowers is not clear (**Fig. 5a**), especially given our hypothesis that the deletion event makes the F allele of VviINP1 protein non-functional. However, high expression may constitute a kind of compensatory effect similar to those reported for CRISPR knock outs (Smits *et al*., 2019).

<u>Page 11 line 304:</u>
The average synonymous distance (dS) between all pairs of F and M alleles of *INP1* was 0.0275 substitutions per base. Assuming a generation time of 3 years and a nucleotide substitution rate of $2.5x10^{-9}$ substitutions per base per year (Zhou *et al*., 2017), we infer that M and F alleles diverged ~16.5 million years ago (mya), a value within the range of uncertainty of the estimated split time between *Vitis* and *Muscadinia* (Wan *et al*., 2013).

A candidate female-suppressing mutation was also examined, but no candidate becomes clear, if I understood correctly (as mentioned, the ms is very difficult to follow). The Discussion section says that "there are several candidates for the sp mutation", and seems to focus on the male-sterility mutation candidate, and says little about the female-suppressor. The text on p. 13 says that "F-linked polymorphisms define a region of the SD that is likely to house the hypothesized recessive sp mutation, because this is where F haplotypes differ from the M and H haplotypes that retain male function. I found this confusing, because I think that this criterion would define the region associated with the difference between males and females (test (i) above), and not the region carrying the female-suppression mutation (So, in the authors' notation).

We agree that this may not be clear, because the region where H and M are mostly alike and differ from F is the region that hosts the dominant male-fertility allele. We have modified the text to clarify the approach and our inferences. We hope the changes make the manuscript more accessible to a broader audience.

Page 16 line 488:

M-linked polymorphisms occur in the 5' region spanning from the promoter region of the PPR-containing protein through to *TPP*, and F-linked polymorphisms span *TPP* through *VviAPT3* (**Fig. 6a**). These distinct regions provide an opportunity to disentangle the genetic determinants of sex trait. For example, under the two-locus model of the origin of dioecy, the dominant female-sterility allele is expected to be unique to M haplotypes, and therefore located in the region where M haplotypes differ from F and H haplotypes. This narrows a search for the female-sterility locus around the region where M-linked polymorphism is elevated (**Fig. 3a-e**). Notably, M-linked SNPs cluster near *VviYABBY3*, and VviYABBY3 protein sequence clearly differentiates M from F and H haplotypes (**Fig. 3f**). In addition, *VviYABBY3* exhibits an M-linked gene expression pattern during flower development (**Fig. 5a**). We therefore hypothesize that one of the key steps in the ancient transition to dioecy was either caused by the amino acid change in the VviYABBY3 protein and/or the upregulation of the *VviYABBY3* gene that caused female sterility in males.

Moreover, it would be true only if recombination occurs (as explained above, associations within a completely linked region cannot be used for such inferences). A previously proposed candidate, APT3, seemed promising because it is expressed in the carpel primordia of male plants, consistent with a role in pistil abortion (and the new expression results on p. 11 support this), but the trees in Fig. 3 now seem to suggest that this is not fully sex-linked. However, this should be checked by a more refined analysis suggested above, comparing just males and hermaphrodites, as tree analysis could be misleading for such a conclusion. The ms mentions that mutants of Arabidopsis [presumably thaliana] APT1 [presumably the A. thaliana ortholog of the APT3 gene in Visit?] "are sterile males" [presumably meaning male sterile]. It does not tell the reader at all clearly what the APT3 results tell us until p. 16, where (after a welter of network analysis results) it is discounted as a candidate for "either the sp or the So mutation". Finally, the WRKY transcription factor is mentioned, and the support for this candidate is that it has lower expression in females than males (and this gene does seem from Fig. 1 to be potentially fully sex-linked), but, confusingly, page 12 discusses whether "WRKY plays a role in male sterility".

We have modified the text to clarify.

Page 17 line 527:
Similarly, F-linked polymorphisms define a region of the SDR that is likely to house the hypothesized recessive male-sterility mutation, because this is where F haplotypes differ from the M and H haplotypes that retain male function. F-linked polymorphisms were observed in the latter portion of the SDR from *TPP* to *VviAPT3* (**Figs. 3 and 6a**); it is in this region that a male-sterility candidate probably resides. Of the genes in the region, *WRKY* and *VviINP1* are the most noteworthy. The gene *WRKY* is poorly expressed in females relative to males (**Fig. 5a**) and is part of a co-expression module that is negatively correlated with female sex (**Fig. 5c**); low *WRKY* expression may participate in male sterility.

Page 17 line 548:
Like *VviINP1, VviAPT3* is in the latter portion of the SDR and exhibits F-linked polymorphism. *A. thaliana apt1* mutants are male sterile (Moffatt and Somerville, 1988; Gaillard *et al*., 1998). However, *VviAPT3* was highly expressed in males, in a co-expression module well-correlated with the male sex, and was previously associated with pistil structure abortion in grapevine, not male sterility (Coito *et al*., 2017). APT proteins promote the inactivation of cytokinins (Allen *et al*., 2002; Zhang *et al*., 2013), which regulate ovule number, gynoecium size (Bartrina *et al*., 2011; Marsch-Martínez *et al*., 2012), flower sex specification (Durand and Durand, 1991; Ni *et al*., 2018), and can restore female organ development in male grapevines (Negi and Olmo, 1966). High expression of the *VviAPT3* allele common to H and M could contribute to female sterility. Alternatively, polymorphisms in the F-linked promoter of *VviAPT3* that integrate hormone signals (**Supplementary Fig. 10a**), as in *A. thaliana* (Ito *et al*., 2007; Song *et al*., 2013; Qi *et al*., 2015), might downregulate gene expression in a manner that contributes to male sterility in female flowers or is required for female fertility. These data implicate *VviAPT3* in flower development and sex determination, though its mechanism of action seems complex and requires further study to fully understand.

Another potential barrier to understanding is that the ms uses the non-standard notation just quoted, deriving from a paper in 1838 by Oberle, which is likely to be inaccessible to many readers (N. Y. Agric. Exp. Sta. Tech. Bulletin). In my view, it is not important enough to recognize Oberle's contribution by adopting his notation, given that Westergaard reviewed many species and unified the notation, and his notation (or minor variants of it) has been widely used since then. Even with a notation that some readers will know, it is difficult to remember which mutation is which, and in many places, it would be better if the text simply said "male-sterility" or "female-suppressing".

We appreciate this comment. We have opted to replace *so* and *Sp* throughout the manuscript, with the exception of **Fig. 6**, where we carefully note that that the *so/Sp* terminology is common in the grape literature. We introduced the *so*/*Sp* terminology making clear that it was used by Oberle specifically in the context of grape sex determination.

Page 18 line 574:

This model is compatible with the one proposed by Oberle (1938) for grapes, who designated *sp* the allele that inhibits pollen development and *So* the allele that inhibits ovule development (**Fig. 6b**).

Minor comments

1. Is it correct that the majority of animals have separate sexes? I have been told that hermaphrodites are commoner than gonochoristic species, so this should be checked.

We have removed the comment.

2. Of course, Darwin pointed out, long before Renner (2014), that dioecy is rare in plants. And the evidence that it has evolved many times in plants, independently in different lineages, was also known long before Renner's article (e.g. Westergaard's 1958 review in Advances in Genetics, vol. 9, pages 217-281, which is unaccountably not mentioned, and Charlesworth's 1985 analysis of the distribution of dioecy and self-incompatibility in angiosperms, pp. 237-268 in Evolution — Essays in Honour of John Maynard Smith, edited by P. J. Greenwood and M. Slatkin).

Thank you for these references. They have been cited.

Page 3 line 33:
Dioecy ensures outcrossing and thus promotes genetic diversity, but it occurs in only 5 to 6% of angiosperms (Westergaard, 1958; Charlesworth, 1985). Despite its rarity, dioecy is widespread phylogenetically, suggesting it has evolved independently on multiple occasions (Westergaard, 1958; Charlesworth, 1985; Ming *et al*., 2011; Käfer *et al*., 2017).

Page 15 line 475:
Dioecy is rare but phylogenetically widespread in angiosperms, suggesting it originated independently on many occasions (Westergaard, 1958; Charlesworth, 1985).

Reviewer #3 (Remarks to the Author):

In general, this is a well written and interesting research paper. The discussion is very well supported with results and the methodology robust. These are, however, some small questions and remarks.
First of all, authors must consider to review the Vitis nomenclature used. For that we advise to read "The grapevine gene nomenclature system; BMC Genomics volume 15, Article number: 1077 (2014)".

We thank the reviewer for the suggestion. Accordingly, we modified the taxonomy ID and the object type ID following the nomenclature instructions described in Grimplet *et al*. (2014). Due to the complexity of our data, *i.e*. genomes from different cultivars/varieties, we adapted the

nomenclature proposed by Grimplet *et al*. (2014). We describe in detail how we have named genes, transcripts, and protein in the methods in supplementary material.

<u>In Supplementary Information, page 12 line 168:</u>
For each genome, gene locus nomenclature was adapted from Grimplet *et al*. (2014):

1. Taxonomy ID: the three letters ‘VIT’ and the code defined by the Vitis International Variety (VIV) Catalogue (http://www.vivc.de/docs/dataonbreeding/AbbrevVitaceae%208Dez10.pdf).
2. Accession ID. For *Vitis vinifera* ssp. *vinifera*, accession ID is composed of the cultivar abbreviation and clone number.
3. Genome assembly version.
4. Genomic sequence.
5. Gene annotation version.
6. Gene numeric code.

Examples:

```
VITVvi_vCabSauv08_v1.1.H0000F_002.ver1.0.g000110
|------------| |------------| |----| |---------------| |-------| |---------|
      1              2          3          4             5         6

VITVvi_sO34-16_v1.1_H0000F_002.ver1.0.g000100
|-----------| |---------| |----| |---------------| |-------| |---------|
      1            2        3          4             5         6
```

Regarding the gene name, no nomenclature system for naming genes at the *Vitis* genus level is proposed in Grimplet *et al*. (2014). Consequently, we contacted Jerome Grimplet and Grant Cramer (senior author of the BMC paper). Following their recommendations, the prefix “*Vvi*” was added to the gene names in the main text as the genes are present in all *Vitis vinifera* as well as in the other *Vitis* spp..

<u>Page 4 line 90:</u>
One such candidate, the adenine phosphoribosyltransferase gene *VviAPT3*, was expressed in the carpel primordial of male plants, suggesting a role in pistil abortion (Coito *et al*., 2017).

<u>Page 10 line 260:</u>
These included one in a gene encoding a trehalose-6-phosphate phosphatase (*TPP*), one in *VviINP1*, seven in an exostosin-coding gene, three in a 3-ketoacyl-acyl carrier protein synthase III gene (*KASIII*), seven in a PLATZ transcription factor (TF)-coding gene (*PLATZ*), eighteen in the first *FMO* gene, twenty-six in the second *FMO*, eleven in the third *FMO*, eleven in the hypothetical protein *VviFSEX*, and four in *VviAPT3*.

<u>Page 10 line 266:</u>

In contrast, we found only six non-synonymous M-linked SNPs: two in the YABBY TF-coding gene *VviYABBY3* (Zhang *et al*., 2019), two in an aldolase-coding gene, one in *TPP*, and one in the third *FMO*.

Page 3:
“The second step is a dominant mutation that suppresses female function (So)”
Should be: “The second step is a dominant mutation that suppresses female function (So), also accordingly to Oberle, 1938)”

The sentence was removed. The introduction was substantially revised to address comments from multiple reviewers. We have added a section that contains general explanations about the two-locus model from Westergaard (1958), as this model has been already used in other plants, such as kiwifruit and asparagus (Akagi *et al*., 2019; Akagi and Charlesworth, 2019; Tsugama *et a*l., 2017). A description of the Oberle’s model was added in the Discussion section.

Page 18 line 574:
This model is compatible with the one proposed by Oberle (1938) for grapes, who designated *sp* the allele that inhibits pollen development and *So* the allele that inhibits ovule development (**Fig. 6b**).

“Thus far, the best candidates come from asparagus, where females lack a gene associated with tapetal development (Tsugama et al., 2017) and a mutant male lost a putative female suppressor (So) gene to become a hermaphrodite (Harkess et al., 2017).”
The So reference in this phrase should be eliminated. The So/so Sp/sp model was build specifically for the grapevine dilemma and has not referred in the work of Harkess et al., 2017.

We agree with the reviewers and the sentence was rephrased.

Page 3 line 54:
The best candidates have been found in asparagus and kiwifruit (Akagi *et al*., 2018, 2019; Harkess *et al*., 2017; Tsugama *et al*., 2017). In asparagus, females lack a gene associated with tapetal development (Tsugama *et al*., 2017) and mutant males without a putative female-suppressor gene become hermaphrodites (Harkess *et al*., 2017).

Page 4:
on «With these extensive data, we address four questions»
Only three questions are raised by authors. Shortly:
1) How M differs from F and H?
2) Are there different gene expression levels?
3) Can we reconstruct the domestication process?

Questions were removed and goals of the study were rephrased.

Page 4 line 109:
With these extensive new sequence and expression data, we compared the F, H, and M haplotypes to better define the SDR, identify candidate sex-determining genes, and reconstruct a key step in the domestication of *Vv vinifera*, namely the recombination event that resulted in the reversion to hermaphroditism observed in domesticates.

Figure 1: The c panel on figure 1 does not fit together with the others panels within this figure. Authors are advised to review the images positioning.

Positioning has been modified with c panel below a and b panels (**Fig. 1**).

Page 5:
"male Muscadinia rotundifolia" and "400 kbp insertion, and the M. Rotundifolia"
Is a bit confusing, either "male muscadine" or "male V. rotundifolia". This issue is recurrent as it appears throughout the manuscript

In order to prevent any confusion, the term "male" is only used for the sex type of the individual while the sex type of the SDR haplotype is abbreviated, *i.e*. the M haplotype of *M. rotundifolia*.

Page 5 line 135:
In addition, the genome of one male *Muscadinia rotundifolia* was constructed as a dioecious outgroup to *Vitis spp*. (**Fig. 1c**; Small, 1903; Moore, 1991; Mullins *et al*., 1992; Liu *et al*., 2016; Wen *et al*., 2018; Zecca *et al*., 2020).

Page 6 line 177:
In addition, the *V. arizonica* M haplotype had a unique ~400 kbp insertion, and the M haplotype of *M. rotundifolia* contained an inversion that encompassed 57% of the SDR (**Fig. 2a**).

Page 6 line 185:
Despite a large inversion, gene content and order in the M haplotype of *M. rotundifolia* was similar to the *Vitis* M haplotypes. The F haplotype of *M. rotundifolia* was identical in gene content and order to the *Vitis* F haplotypes (**Fig. 2d**).

"three hermaphrodite Vv vinifera"
Would benefit having the cultivars names: "three hermaphrodite Vv vinifera cultivars (Merlot, Black Corinth seedless and Black Corinth seeded)"

Cultivars names were added.

Page 5 line 132:
To investigate the structure and evolution of the SDR in *Vitis* spp., we sequenced and assembled the complete genomes of eight *Vitis* accessions, including three hermaphrodite *Vv vinifera*

cultivars (Merlot, Black Corinth seedless and Black Corinth seeded), four *Vv sylvestris* accessions (two females and two males), and one male *V. arizonica*.

"Relative to H and Vv M haplotypes,"
Should be: "Relative to H and M haplotypes,"

Vv was referencing *Vitis vinifera*, as the TPR-containing proteins are not present in the M haplotype of *V. arizonica*. Sentence was rephrased to avoid any confusion.

<u>Page 6 line 181:</u>
Relative to H and *Vv sylvestris* M haplotypes, F haplotypes had a deletion of two genes that encode TPR-containing proteins.

Page 12:
A blank line is missing between the legend of Figure 5 and the manuscript text.

Thank you for the observation.

Page 13:
Ramos et al 2014 has a similar diagram with fig. 6. How do your findings relate to the hypothesis raised by these authors? Or vice versa.
There is also some talk about the So and Sp but little regarding to what they mean. When talking about figure 6, would be nice to have some background regarding the so/So and sp/Sp and its origin. Also interesting would discuss why based on these findings a model like so/So; sp/Sp is more adequate than for example the M F H model. Despite the terminology M, F, H being used for the haplotypes for the sake of clarity.

The discussion now includes a comment on the sex determination model, the origin of the *so*/*Sp* allele designation, and the literature that supports to the gynodioecy path.

<u>Page 18 line 574:</u>
This model is compatible with the one proposed by Oberle (1938) for grapes, who designated *sp* the allele that inhibits pollen development and *So* the allele that inhibits ovule development (**Fig. 6b**). Like Ramos *et al*. (2014), we propose that female grapes arose via hermaphroditic selfing. Because male-sterility mutations leading to gynodioecy is the more likely path based on population genetic modelling (Charlesworth and Charlesworth, 1978), we hypothesize that the male-sterility mutation arose first.

Page 15:
When the authors question why the haplotypes remain distinct, one simpler hypothesis, along with the possibilities raised by the authors, is the one point by Oberle when he suggested the two

close linked loci. In the case of this work, if YABBY and INP1 are close then recombination between the two loci would be rare.

Excellent suggestion! We added it to the Discussion.

Page 19 line 620:
The SDR in grapevine is also relatively short and the close proximity of sex-determining genes may serve as an impediment to their recombination (Oberle 1938).

F1 populations:
What were the segregation rates (and $\chi 2$) of the F1 populations?
If we counted correctly, one cross resulted in 100 females and 118 males and the other cross in 78 females and 100 males.
There is also some confusion regarding the F1 offspring phenotype (supplementary table 4a and 4b). What is the meaning of the NA plants, they don't have a visible phenotype yet (haven´t yet bloom) or INP1 amplification was inconclusive?

NA indicates plants that did not produce any inflorescence. This information was added to the **Supplementary Table 4**: "NA, no visible phenotype (no inflorescence)."

Information about segregation of the sex trait was added in the manuscript. χ^2 values and associated df and P value were added to the text.

Page 11 line 320:
Among the 218 individuals of the *Vv vinifera* x *V. arizonica* F1 population, 102 individuals were males, 100 females and 16 did not produce any inflorescences, while the *Vv vinifera* x *Vv sylvestris* F1 population was composed of 92 males, 78 females and 8 plants without inflorescences. In both F1 populations, sex trait segregation followed a 1:1 ratio, as expected from a FF x MF cross (χ^2 = 0.02, df = 1, P value = 0.890; χ^2 = 1.15, df = 1, P value = 0.283).

The amplification of INP1 in the F1 populations showed that male plants had at least one functional INP1 allele. What about the YABBY gene? Did the SV in the YABBY gene and promoter segregate in the F1 according to sex?

Two F1 populations are a remarkable source of information that the authors should explore. Mainly in order to confirm the hypothesis proposed and see how they hold compared with older Mendelian studies (Oberle 1938; Avramov et al 1967; Negi and Olmo 1971, for example)

We totally agree with the reviewers. In addition to confirming the 1:1 segregation ratio, we performed a bulk segregant analysis including 120 individuals of the *Vv vinifera* x *V. arizonica* F1. The analysis allowed to confirm the complete sex linkage of all the SNPs and INDELs we identified as well as to confirm the location of sex-determining region on chromosome 2. Regarding *VviYABBY3*, we added pictures of alignments confirming the position of the two non-

synonymous SNPs in *VviYABBY3* M allele and the M-linked SNPs identified in its promoter region in **Supplementary Fig. 8 c-d**.

Page 11 line 329:
The 8 bp deletion in *VviINP1* as well as all other sex-linked polymorphisms in the SDR were confirmed by replicated bulk analysis of 120 individuals of the *Vv vinifera* x *V. arizonica* F1 population genotyped by whole genome resequencing using Illumina technology (**Supplementary Fig. 8**).

In Supplementary Information, page 8 line 82:
Supplementary Fig. 8: Bulk segregant analysis on 120 individuals from the population *Vv vinifera* F2-35 x *V. arizonica* b42-26.
a, The number of sex-linked SNPs per 100 kbp across Cabernet Sauvignon hap1 genome. SNPs were identified by aligning short-read sequencing data from 120 individuals of the F1 population *Vv vinifera* F2-35 x *V. arizonica* b42-26 split in four bulks, two composed of female individuals (FF), called F1 and F2, two made of male individuals (MF), called M1 and M2. SNPs in homozygous state in both female pools (0/0/0/0 or 1/1/1/1) and in heterozygous state in both male pools (0/0/1/1) were considered fully sex-linked. Bulk segregant analysis confirmed the location of the sex-determining region on chromosome 2. Alignment of the short-read sequencing data confirm complete sex linkage of the 8 bp deletion in *VviINP1* (**b**), as well as the two non-synonymous SNPs in *VviYABBY3* (**c**) and SNPs identified in its promoter region (**d**).

Supplementary figure 7:
In the panel B there is alleles F and M but from what we understand from the manuscript, female plant is FF and so the alleles would be FF and not F H as it is shown in the figure. Similarly, male plants would be MF and not HF was inferred by the figure.

In Supplementary Fig. 7 panel B, we represented the number of normalized read counts mapping on both Cabernet Sauvignon alleles, H and F, of *VviAPT3*. Mapping of the RNA-seq reads was performed on the entire genome of Cabernet Sauvignon in a non-deterministic way. For each gene of the sex-determining locus, read counting was done by allele, i.e. H and F alleles separately. In absence of sequence variation, RNA-seq reads from the female flower (FF) mapped evenly on both F and H haplotypes. That's why female flower (FF) depicted counts for both F and H haplotypes, nonetheless with a higher level of expression of the F allele due to sequence similarity.

Information was added to the caption of Supplementary Fig. 10 to clarify:

In Supplementary Information, page 10 line 101:
Supplementary Fig. 10: *VviAPT3*, sex-linked factor-binding sites, gene expression and allele usage.
a, Sex-linked transcription factor-binding sites found within 3 kbp region upstream of *VviAPT3* transcription start site (TSS). **b**, *VviAPT3* gene expression during floral development in three sex types. Mapping of the RNA-seq reads was performed on the entire genome of Cabernet Sauvignon in a non-deterministic way. For each gene of the sex-determining locus, read counting

was done by allele, *i.e.* H and F alleles separately. In absence of sequence variation, RNA-seq reads were assigned evenly to F and H haplotypes. **c**, *VviAPT3* allele usage by flower sex at each developmental stage. Abbreviations: F, female; H, hermaphrodite; M, male.

This article was reviewed by: Margarida Rocheta; Lucas Coito and Miguel de Jesus Nunes Ramos from the same group.

Reviewer #4 (Remarks to the Author):

The authors studied the structure and evolution of the sex determination region in grape species to better understand the origin of dioecy and how dioecy was lost through the domestication. They identified a candidate male-sterility mutation in the INP1 gene and potential female-sterility function associated with a transcription factor. Moreover, the identification of two candidate genes encourage future testable hypotheses concerning their putative evolution and function.
This is an interesting manuscript which provides new insights on the domestication process in grapevine. The manuscript is clear for most parts and pleasant to read. I do not have experience with some analysis implemented in the manuscript. However, the choice of methods seems appropriate and the methodology is adequate. The results are in line with the aims and show which genes are probably involved in the domestication process.
I have some specific comments that show below (I am surprised not to see the numbers of the lines along the manuscript. This is very unpleasant. However, I have indicated the number of the line to which my comments refer, hoping to use the same pdf file.)

Line numbers were added to the manuscript. We apologize for the inconvenience.

Title: Your study involves different species from Vitis vinifera to Muscadine. Usually, the grapevine is referred to Vitis vinifera L. I suggest changing the word grapevine with grapes, in the title.

Title was modified as suggested.

Introduction: Page 3, Line33. I have some doubts about the time of the split of the genus Vitis. Effectively, Ma et al., 2019 show a split about 47 millions of years ago but this result is influenced by calibration models, choose of outgroups and fossil data. In particular, divergence times estimations can be affected by interpretation of fossil data and the number of calibration points applied (Rutschmann et al. Syst Biol 2007; Zecca et al., Current genetics 2019). As you can see the paper table1 in Wan et al., (BMC Evolutionary Biology 2013) are recognized different times of split for the crown of Vitis subgenus. Moreover, other values are recognized in more recent papers. Thus, I suggest that the authors should avoid showing the time of divergence of the Vitis, unless you don't choose to show the alternative results.

We understand your concern. Accordingly, the sentence was removed.

Results: Page 5, line 6. You have rooted the tree with M. rotundifolia. I agree but you should add a reference about phylogenetic studies and a short comment showing that this species is distant from all other grapes. If you need references, see GRIN taxonomy or Flora of North America.

Sentence was rephrased and references were added as suggested.

Page 5 line 135:
In addition, the genome of one male *Muscadinia rotundifolia* was constructed as a dioecious outgroup to *Vitis spp.* (**Fig. 1c**; Small, 1903; Moore, 1991; Mullins *et al*., 1992; Liu *et al*., 2016; Wen *et al*., 2018; Zecca *et al*., 2020).

Fig. 1 caption, page 5 line 121:
A phylogenetic tree predicted from whole-genome proteome orthology separates species by taxonomy and not by sex genotype. *M. rotundifolia* is an outgroup to the *Vitis* ingroup (Liu *et al*., 2016; Wen *et al*., 2018; Zecca *et al*., 2020).

Discussion: Page 13, Line3. I expected to read a profound discussion about the YABBY gene. The function of this gene is widely discussed for other species; you should have no problem finding articles about it. In addition, there are recent articles also related to the genus Vitis (Zhang et al., Frontiers in Plant Science 2019; Xiang et al., Protoplasma 2013). I suggest you, to compare your results with other studies and so to improve the discussion.

We thank the reviewer for this suggestion. Potential role of VviYABBY3 has been discussed.

Page 16 line 501:
Functional information about YABBY gene family is consistent with this hypothesis. In *A. thaliana*, YABBY genes are involved in floral and lateral organ development (Chen *et al*., 1999; Sawa *et al*., 1999; Siegfried *et al*., 1999, Bowman *et al*., 1989; Bowman, 2000), specifically the development of carpels and the ovule outer integument (Villanueva *et al*., 1999). Though VviYABBY3 is not yet characterized, expression of *Vitis pseudoreticulata VpYABBY1* and *VpYABBY2* in *A. thaliana,* and *VvYABBY4* in tomato implicate these genes in leaf and carpel growth and development (Xiang *et al*., 2013; Zhang *et al*., 2019).

Discussion: Page 13, Line4. “Among all protein phylogenies, YABBY clearly differentiates M haplotypes from F and H haplotypes”. I don’t agree. There are other protein phylogenies that show clearly differentiates M haplotypes from F and H. The authors should modify the sentence.

The sentence was modified.

Page 16 line 496:

Notably, M-linked SNPs cluster near *VviYABBY3*, and VviYABBY3 protein sequence clearly differentiates M from F and H haplotypes (**Fig. 3f**). In addition, *VviYABBY3* exhibits an M-linked gene expression pattern during flower development (**Fig. 5a**).

Discussion: page 14, line23. I agree with the authors that the highly expressed of INP1 gene in female is a mystery. However, also your answer is a mystery for me ."...High expression of INP1 in females could reflect an attempt to compensate for the 8 bp deletion that renders the protein non-functional." I don't understand how a cell can compensate for this. This sentence should be explained better and highly supported by data and references.

We understand your concern. Sentence was modified and a reference was added.

<u>Page 17 line 543:</u>
Why *VviINP1* was more highly expressed in female flowers is not clear (**Fig. 5a**), especially given our hypothesis that the deletion event makes the F allele of VviINP1 protein non-functional. However, high expression may constitute a kind of compensatory effect similar to those reported for CRISPR knock outs (Smits *et al.*, 2019).

Discussion: page 14, line10. I don't understand where to find the 60% value in your results. Perhaps, do you mean 57%?

We did mean 57%. The sentence was corrected.

<u>Page 19 line 609:</u>
The question's answer is obvious for *M. rotundifolia*, because 57% of the M haplotype is inverted relative to the F haplotype.

Discussion: page 14. "..Length differences between F and M haplotypes play some role in recombination deterrence,.." This is very interesting. Are there other studies that support this? Can you support this hypothesis with adequate literature?

Haplotypic structural variability has been showed to strongly affect the frequency and distribution of recombination events in maize (He and Dooner, 2009). Reference was added.

<u>Page 19 line 618:</u>
Recombination may be deterred by differences in structure and length between M and F haplotypes, which are largely attributable to TE accumulation in intergenic space (He and Dooner, 2009).

Materials and methods: pag19, Phylogenetic analysis. You have applied the Maximum-Likelihood (ML) method, assuming the evolutionary model LG+G8+F. How have you chosen

the model? Do you have used software? You should indicate the name of the software and how you have set it.

Phylogenetic analysis was performed using RAxML-NG v.0.9.0 with the option --opt-model to optimize the evolutionary model in function of the data (Kozlov *et al*., 2019; Stamatakis, 2014). The evolutionary model 'LG+G8m+F' was provided by RAxML-NG as optimized evolutionary model considering our data. This information was added to the Methods section.

Page 24 line 827:
Next, phylogenetic analysis was performed using RAxML-NG v.0.9.0 with the option --opt-model to optimize the evolutionary model in function of the data (Kozlov *et al*., 2019; Stamatakis, 2014). We applied the Maximum Likelihood (ML) method with the optimized evolutionary model 'LG+G8m+F', using twenty random starting trees, a boostrapping of 200 replicates and the Gblocks parsed alignments. The analysis was supervised using the Approximate-ML tree produced by OrthoFinder.

Fig. 3: For me is very difficult to find the NP1 gene in Figure. Can you find a better solution to indicate this gene in Figure?

In **Fig. 3**, genes affected by nonsense mutations are now indicated with an "X" and the affected haplotype(s) followed by the corresponding gene abbreviation in parenthesis. In this way, *VviINP1* is better indicated.

Reviewers' comments:

Reviewer #1 (Remarks to the Author):

The authors addressed my questions, and no more comment.

Reviewer #2 (Remarks to the Author):

The English is still shaky and needs serious work by a native English speaker, such as Dr. Gaut, who is an author. The revisions should include omitting repetitions that are unnecessary, some of which I note below. The text is also not completely accurate in many places, or is vague. Presumably the yellow highlighting indicates revised text?

Statements in the responses

The abstract gives the impression that good candidates for the sex-determining genes have been found, and this is surely one of the reasons why the editors considered the manuacript likely to be promising. The abstract says that the study "identified a candidate male sterility mutation in the VviINP1 gene and potential female-sterility function associated with the transcription factor VviYABBY3". The wording in the responses is much more cautious. As the genes are not identified, my opinion is that this ought to be made clear early on, rather than using words that raise the expectation that the genes might be identified.

The abstract says that the work "significantly refines the model of sex determination in Vitis", but does not state what specific refinement is achieved. Why be so vague? Why not tell readers what this is?

What has actually been achieved should be laid out. Long-read sequencing has been done for several species. It allows a better assembly than previously. It defines a sex-linked region better than previously. More than one of these related species appear to share the same sex-linked region. This is not surprising, but worth knowing, as it suggests something about the age of the sex-determining system, and the age estimated from divergence (dS) between sequences in the M and F haplotypes is ~16.5 MY, consistent with this. This is not a very young system. It is, however, physically small (the haplotypes ranged from ~171.6 to only ~837.4 kilobases). This is similar to results from some other plants, including asparagus, for example.

The information about the divergence estimates is not easy to find. The text should mention where these data are shown, and details should be shown, to inform readers about how many X-Y gene pairs were used for the estimates, their coding sequence lengths, or confidence intervals on the estimates. The number of genes that are still X-Y gene pairs (as opposed to being present on the X and missing from the Y) is potentially interesting, as loss of genes is expected from old-established Y-linked regions. It therefore seems strange not to mention whether all the genes in this region of the X (or F) haplotype are also still present in the Y (or H) one, or not, and, if not, why genetic degeneration might not have occurred. Perhaps the roughly 15 genes shown in Figure 1 are not enough, or not big enough, to have led to degeneration?

The finding of an inversion is not as illuminating as it is portrayed, because rearrangements are expected after recombination become suppressed, and unless there is some evidence supporting the idea that the inversion caused the recombination suppression, one cannot suggest that it did. The text says "The answer is obvious for M. rotundifolia, because 57% of the M haplotype is inverted relative to the F haplotype" (which is incorrect, as it is true only if one assumes the answer), and it then says that inversions are "likely to slow [meaning impede] recombination between the haplotypes", but in my opinion this is too strong, as readers are likely to think that recombination suppression is generally caused by inversions, so one should be very careful not to give this impression. It is illogical to say

that because inversions have been shown to reduce recombination, that the presence of an inversion must do so in this case — it may have done, or maybe not. In this context, the ms later suggests that TE accumulation might prevent recombination (although again the higher TE density in low recombination genome regions is a matter of which is cause and which effect), and it is surely not invariably the case that TEs accumulate only in intergenic space, as line 619 suggests.

Overall, a calmer and better organized presentation would be desirable.

Some detailed comments and examples follow
Abstract
chromosome-scale Cabernet Sauvignon reference GENOME SEQUENCE, and the phased ….|
"to contrast male…." In line 21 should read "to compare male….
What does "regions of sex-specific function" mean? Is it meant to mean "fully sex-linked regions that include the sex-determining gene or genes"? Why is it "regions"? Are these several regions? If there is just one fully sex-linked region, then surely the singular should be used.
The phrase "Our data support that dioecy was lost…", should be corrected to "Our data support the conclusion that dioecy was lost…".

Introduction
It is misleading to write that "Dioecy ensures outcrossing and thus promotes genetic diversity", as genetic diversity is not a selective reason for the evolution of dioecy. It would be better to write simply "Dioecy ensures outcrossing". There is o need to repeat ideas. Why not simply say "About 5 to 6% of angiosperms species have separate male and female individuals, a mating system called dioecy, which ensures outcrossing"

It is misleading to write that "the two-locus model …. assumes …. two steps". Papers on this model point out that 2 steps are required.

It is strange to give credit to Henry et al., 2018 for the understanding that a two-locus system can maintain separate sexes only if the two loci are completely linked, because recombination between them could restore hermaphrodites. Westergaard explained this clearly many years earlier, and Bull's classic book on sex determination and sex chromosomes does also, and was published in 1983.

Bull, J. J., 1983 Evolution of Sex Determining Mechanisms. Benjamin/Cummings, Menlo Park, CA.

Westergaard's review also explained the empirical support for this model, and ought to be cited, as his evidence is much stronger than most of the other studies mentioned. The recent paper by Harkess et al. should also probably be cited.

Although it is possible that recombination event between male and female haplotypes generated the hermaphrodites, mutation is also possible if the male-sterility mutation is something that can revert, like a single base mutation. This question should be related to the conclusions about the male-sterility mutation. It is unnecessary to repeat information here, and the phrase "As a consequence of this reversion, Vitis spp. have individuals of three sexes (Negi and Olmo, 1970)" can be omitted, and just the figure of the sex morphs in the domesticated species shown. As only this species shows the hermaphrodite morph, you can use that observation to be stronger than "Presumably the shift of mating system occurred during domestication". The text about the genetic basis can be shortened by explaining that the hermaphrodite appears to have a Y-lined region, as in the similar papaya system (also involving domestication".

Strangely, line 112 states that a recombination event definitely occurred, and that the study will reconstruct a key step in the domestication of Vv vinifera, namely
the recombination event observed in domesticates", so it would have been better to say in the earlier

text that this will be tested.

"The partially resolved sequences of H and F haplotypes, showed that they differ in the presence and absence of three genes". Some specifics should be given, for example, whether these are genes that are present on the F haplotype and absent from the H one, as is often seen under genetic degeneration of Y chromosomes, or some other kind of difference. It's unclear what "This work" in line 98 means — is it this new work, or are you referring to the Zhou et al., 2019b paper? The sentence "Yet, despite substantial progress our understanding of the SDR and the potential determinants of sex have been hampered by the absence of information from M haplotypes" is an unnecessary repeat.

A comma is needed in line 107, after "manually curated".

Results
The repetition of the goal "To investigate the structure and evolution of the SDR in Vitis spp." In line 132 can be omitted.

In line 136, the English is strange: "the genome of one male Muscadinia rotundifolia was constructed" means something like "the genome of a male Muscadinia rotundifolia was sequenced".

Similarly, corrections are needed to the text "Each Vitis SDR haplotype was aligned to the Cabernet Sauvignon H haplotype to determine the structural differences among haplotypes [at this point, no structural differences have been mentioned, so readers don't know what this means, though perhaps it means simply to test for structural differences], and identify features that are conserved in a sex-specific manner" [maybe meaning identify sex-specific features"].

Tenses are sometimes incorrect, for example in line 171 "These length differences reflected the presence of sex-linked SVs" (should read "reflect").

Line 621 is an example of unnecessarily long-winded writing that is difficult to understand ("close proximity of sex-determining genes may serve as an impediment to their recombination", means "close linkage of sex-determining genes may simply reflect physical closeness").

Several places have odd English, including "observations [or data] support that", which ought to be "support the view [or interpretation, or working hypothesis] that"

Reviewer #3 (Remarks to the Author):

The authors significantly improved the article. For our part we are satisfied.
Just a small detail that we forgot in the previous review: the authors refer to the existence of three sexes, (pag.3 line 70 and pag.4, line 75).
Please consider: in our world there are only two sexes: male and female. Nowadays this may be a philosophical question, but as we are talking about flowers perhaps the designation of "flower type" is appropriate, when you need to refer male, female and hermaphrodite. And so, we have two sexes but three flower types.

This manuscript was revised by: Margarida Rocheta, Miguel Ramos and João Coito

Reviewer #4 (Remarks to the Author):

I thank the authors for accurate answers and the changes in the text. In my opinion, the authors have

sufficiently addressed the revisions and submitted information to include from the previous draft. I consider that this manuscript provides new and interesting results and thus I suggest publishing.

Response to Reviewers – Authors' answers are in blue

Reviewer #1 (Remarks to the Author):

The authors addressed my questions, and no more comment.

Reviewer #2 (Remarks to the Author):

The English is still shaky and needs serious work by a native English speaker, such as Dr. Gaut, who is an author. The revisions should include omitting repetitions that are unnecessary, some of which I note below. The text is also not completely accurate in many places, or is vague. Presumably the yellow highlighting indicates revised text?

We carefully reviewed every word and sentence of the manuscript to improve and clarify the presentation of the manuscript to every extent possible (yellow highlighting indicates revised text).

Statements in the responses

The abstract gives the impression that good candidates for the sex-determining genes have been found, and this is surely one of the reasons why the editors considered the manuacript likely to be promising. The abstract says that the study "identified a candidate male sterility mutation in the VviINP1 gene and potential female-sterility function associated with the transcription factor VviYABBY3". The wording in the responses is much more cautious. As the genes are not identified, my opinion is that this ought to be made clear early on, rather than using words that raise the expectation that the genes might be identified.

We agree with the reviewer that the genes we identified are only candidates and were not functionally characterized in this study. Accordingly, a sentence was added to the discussion and sentences in the results and abstract were modified as detailed below.

Page 17 line 542:

"Given our hypothesis that the *VviINP1* deletion leads to male sterility, an important future step will be functional confirmation that a homozygous deletion engineered into a hermaphrodite produces female flowers."

In the VviINP1 result section, page 11 line 336:

"Together, the sequence, phylogenetic, association, and functional evidence suggest that a recessive allele of *VviINP1* containing an 8 bp deletion interrupts male function, making *VviINP1* a plausible male-sterility candidate."

In the Abstract we carefully refer to these two genes as "candidate male-sterility mutation" and "potential female-sterility function".

Page 2 line 23:

"Coupled with gene expression data, we identified a candidate male-sterility mutation in the *VviINP1* gene and potential female-sterility function associated with the transcription factor *VviYABBY3*."

The abstract says that the work "significantly refines the model of sex determination in Vitis", but does not state what specific refinement is achieved. Why be so vague? Why not tell readers what this is?

The sentence was modified to avoid referring to the model. The advancements in understanding of the genetic basis of sex determination in grapes are described in the previous sentences of the abstract.

Page 2 line 27:

"This work significantly advances the understanding of the genetic basis of sex determination in *Vitis* and provides the information necessary to rapidly identify sex types in grape breeding programs."

What has actually been achieved should be laid out. Long-read sequencing has been done for several species. It allows a better assembly than previously. It defines a sex-linked region better than previously. More than one of these related species appear to share the same sex-linked region. This is not surprising, but worth knowing, as it suggests something about the age of the sex-determining system, and the age estimated from divergence (dS) between sequences in the M and F haplotypes is ~16.5 MY, consistent with this. This is not a very young system. It is, however, physically small (the haplotypes ranged from ~171.6 to only ~837.4 kilobases). This is similar to results from some other plants, including asparagus, for example.

The information about the divergence estimates is not easy to find. The text should mention where these data are shown, and details should be shown, to inform readers about how many X-Y gene pairs were used for the estimates, their coding sequence lengths, or confidence intervals on the estimates. The number of genes that are still X-Y gene pairs (as opposed to being present on the X and missing from the Y) is potentially interesting, as loss of genes is expected from old-established Y-linked regions. It therefore seems strange not to mention whether all the genes in this region of the X (or F) haplotype are also still present in the Y (or H) one, or not, and, if not, why genetic degeneration might not have occurred. Perhaps the roughly 15 genes shown in Figure 1 are not enough, or not big enough, to have led to degeneration?

Details about the divergence estimate were added.

Page 11 line 299:

"The sex-specificity of *INP1* alleles provided an opportunity to estimate the divergence date between M and F haplotypes and hence the potential age of dioecy. We calculated the average synonymous distance (dS) between all 52 pairs of F and M alleles of *INP1* to be 0.0275

substitutions per base (95% confidence interval: 0.0258 - 0.0292). Assuming a generation time of 3 years and a nucleotide substitution rate of $2.5x10^{-9}$ substitutions per base per year (Zhou *et al*., 2017), we infer that M and F alleles diverged ~16.5 million years ago (95% confidence interval: 15.5 - 17.5), a value within the range of uncertainty of the estimated split time between *Vitis* and *Muscadinia* (Wan *et al*., 2013)."

In addition, number of shared genes within the SDR has been added in the text.

Page 6 line 178:

"While all twenty *Vitis* SDR haplotypes shared 13 SDR genes, two SVs altered gene content."

The finding of an inversion is not as illuminating as it is portrayed, because rearrangements are expected after recombination become suppressed, and unless there is some evidence supporting the idea that the inversion caused the recombination suppression, one cannot suggest that it did. The text says "The answer is obvious for M. rotundifolia, because 57% of the M haplotype is inverted relative to the F haplotype" (which is incorrect, as it is true only if one assumes the answer), and it then says that inversions are "likely to slow [meaning impede] recombination between the haplotypes", but in my opinion this is too strong, as readers are likely to think that recombination suppression is generally caused by inversions, so one should be very careful not to give this impression. It is illogical to say that because inversions have been shown to reduce recombination, that the presence of an inversion must do so in this case — it may have done, or maybe not. In this context, the ms later suggests that TE accumulation might prevent recombination (although again the higher TE density in low recombination genome regions is a matter of which is cause and which effect), and it is surely not invariably the case that TEs accumulate only in intergenic space, as line 619 suggests.

We thank the reviewer for this comment. This discussion point was revised according the reviewer's guidance.

Page 18 line 576:

"Finally, we address one more question about recombination: if recombination can occur between F and M alleles, then what has kept the two haplotypes distinct for so long, given that dioecy has been maintained in the wild since the origin of the genus? This is an especially important question given the hypothesis that the rarity of dioecy among angiosperms is due to easy reversion to hermaphroditism (Käfer *et al*., 2017). We speculate that recombination between M and F haplotypes is deterred by at least three features of the *Vitis* SDR. The first is that the close linkage of sex-determining genes may simply reflect physical closeness (Oberle 1938). If we are correct in our hypotheses that *VviYABBY3* and *VviINP1* are the sterility genes, then recombination events must occur in < 100 kbp that separates the two genes to produce an H haplotype. The second is that not all recombination events will be successful in nature: only 50% of correct recombinants will become hermaphrodites (**Fig. 6b**), and there can be fitness costs associated with hermaphroditism (Charlesworth and Charlesworth, 1978). Finally, we suspect that differences in the structure and length of M and F haplotypes, which are largely attributable to TE accumulation in intergenic space, limit recombination, because recombination can be slowed by differences in

TE content between alleles (He and Dooner, 2009). In this context, the inversion in *M. rotundifolia,* which affects 57% of the M haplotype relative to the F haplotype, may be an especially effective deterrent, because inversions can be barriers to recombination (Lemaitre *et al*., 2009; Wang *et al*., 2012a). We suspect that these three features contribute to the conspicuous absence of hermaphroditic grapes in the wild."

Overall, a calmer and better organized presentation would be desirable.

We went through the manuscript thoroughly and edited it to improve readability and accuracy.

Some detailed comments and examples follow
Abstract
chromosome-scale Cabernet Sauvignon reference GENOME SEQUENCE, and the phased ….|
"to contrast male…." In line 21 should read "to compare male….

We have modified the two sentences as suggested by the reviewer.

Page 2 line 18:
"Our work reports an improved, chromosome-scale Cabernet Sauvignon reference genome sequence and the phased assembly of nine new wild and cultivated grape genomes."

Page 2 line 20:
"By resolving twenty *Vitis* SDR haplotypes, including the first for males, we were able to compare male, female, and hermaphrodite haplotype structures and to identify sex-linked regions that include the sex-determining genes."

What does "regions of sex-specific function" mean? Is it meant to mean "fully sex-linked regions that include the sex-determining gene or genes"? Why is it "regions"? Are these several regions? If there is just one fully sex-linked region, then surely the singular should be used.

The sentence was modified as suggested.

Page 2 line 20:
"By resolving twenty *Vitis* SDR haplotypes, including the first for males, we were able to compare male, female, and hermaphrodite haplotype structures and to identify sex-linked regions that include the sex-determining genes."

The phrase "Our data support that dioecy was lost…", should be corrected to "Our data support the conclusion that dioecy was lost…".

The sentence was modified as suggested.

Page 2 line 25:
"Our data also suggest that dioecy was lost during domestication through a rare recombination event between male and female haplotypes."

Introduction

It is misleading to write that "Dioecy ensures outcrossing and thus promotes genetic diversity", as genetic diversity is not a selective reason for the evolution of dioecy. It would be better to write simply "Dioecy ensures outcrossing". There is o need to repeat ideas. Why not simply say "About 5 to 6% of angiosperms species have separate male and female individuals, a mating system called dioecy, which ensures outcrossing"

We modified the text as suggested.

Page 3 line 34:

"Dioecy ensures outcrossing, but it occurs in only 5 to 6% of angiosperms (Westergaard, 1958; Charlesworth, 1985)."

It is misleading to write that "the two-locus model …. assumes …. two steps". Papers on this model point out that 2 steps are required.

The sentence was rephrased as requested.

Page 3 line 41:

"A common hypothesis about the origin of dioecy is the two-locus model, which requires that dioecy evolved from an hermaphroditic ancestor in two steps (Westergaard, 1958; Charlesworth and Charlesworth, 1978; Charlesworth, 2016)."

It is strange to give credit to Henry et al., 2018 for the understanding that a two-locus system can maintain separate sexes only if the two loci are completely linked, because recombination between them could restore hermaphrodites. Westergaard explained this clearly many years earlier, and Bull's classic book on sex determination and sex chromosomes does also, and was published in 1983.

Bull, J. J., 1983 Evolution of Sex Determining Mechanisms. Benjamin/Cummings, Menlo Park, CA.

We added the suggested references.

Page 3 line 47:

"This two-locus system can maintain separate sexes only if the two loci are completely linked, because recombination between them could restore hermaphrodites (Westergaard, 1958; Bull, 1983; Henry *et al.*, 2018)."

Westergaard's review also explained the empirical support for this model, and ought to be cited, as his evidence is much stronger than most of the other studies mentioned. The recent paper by Harkess et al. should also probably be cited.

We added the suggested references.

Page 3 line 50:

"Support for the two-locus model has been found in several species (Westergaard, 1958), including papaya (*Carica papaya*; Wang *et al.*, 2012a), strawberry (*Fragaria virginiana*; Spigler *et al.*, 2008), *Silene latifolia* (Fujita *et al.*, 2011), *Actinidia* spp. (Akagi *et al.*, 2014, 2019) and grapes (*Vitis* spp.; Picq *et al.*, 2014)."

Page 3 line 56:
"In asparagus, for example, females lack a gene associated with tapetal development (Tsugama *et al.*, 2017) and mutant males without a putative female-suppressor gene revert to hermaphrodites (Harkess *et al.*, 2017; 2020)."

Although it is possible that recombination event between male and female haplotypes generated the hermaphrodites, mutation is also possible if the male-sterility mutation is something that can revert, like a single base mutation. This question should be related to the conclusions about the male-sterility mutation. It is unnecessary to repeat information here, and the phrase "As a consequence of this reversion, Vitis spp. have individuals of three sexes (Negi and Olmo, 1970)" can be omitted, and just the figure of the sex morphs in the domesticated species shown.

We have modified the sentence to avoid repetitions. But we have not removed the description of the three flower types. We think it is important to describe them, particularly for an audience that is not familiar with grape flower morphology.

Page 3 line 69:
"Therefore, *Vitis* spp. have individuals of three types (Negi and Olmo, 1970; **Fig. 1a,b**)"

As only this species shows the hermaphrodite morph, you can use that observation to be stronger than "Presumably the shift of mating system occurred during domestication".

We agree with the reviewer and removed "presumably".

Page 3 line 66:
"This shift of mating system occurred during domestication ~8,000 years ago (This *et al.*, 2006; McGovern *et al.*, 2017), perhaps following a rare recombination event between male (M) and female (F) haplotypes (Picq *et al.*, 2014; Henry *et al.*, 2018; Zhou *et al.*, 2019a)."

The text about the genetic basis can be shortened by explaining that the hermaphrodite appears to have a Y-lined region, as in the similar papaya system (also involving domestication".

We think that an audience that is not familiar with sex determination would benefit from this explanation.

Strangely, line 112 states that a recombination event definitely occurred, and that the study will reconstruct a key step in the domestication of Vv vinifera, namely the recombination event observed in domesticates", so it would have been better to say in the earlier text that this will be tested.

We agreed with the reviewer and modified the last sentence of the introduction to clarify that one of the study's goals was to assess the role of recombination in the development of the H haplotype.

Page 4 line 110:
"With these extensive new sequence and expression data, we compare the F, H, and M haplotypes to better define the SDR, identify candidate sex-determining genes, and assess whether H haplotypes owe their origin to a recombination event."

"The partially resolved sequences of H and F haplotypes, showed that they differ in the presence and absence of three genes". Some specifics should be given, for example, whether these are genes that are present on the F haplotype and absent from the H one, as is often seen under genetic degeneration of Y chromosomes, or some other kind of difference. It's unclear what "This work" in line 98 means — is it this new work, or are you referring to the Zhou et al., 2019b paper?

We added the requested information.

Page 4 line 95:
"More recent work has resolved the partial sequence of four SDR haplotypes, including three H and one F haplotypes (Zhou *et al.*, 2019b). Comparison between H and F haplotypes revealed that they differ in two genes encoding TPR-containing proteins that are present only in H haplotypes (Zhou *et al.*, 2019b)."

The sentence "Yet, despite substantial progress our understanding of the SDR and the potential determinants of sex have been hampered by the absence of information from M haplotypes" is an unnecessary repeat.

The sentence mentions for the first time the lack of M haplotypes as an impediment to the study of sex determination in grapes. We also think it provides the necessary logical link with the following paragraph. We have decided not to remove it.

A comma is needed in line 107, after "manually curated".

The comma was added as suggested.

Page 4 line 107:
"For each genome, haplotypes within the genetically defined SDR have been curated manually, and transcripts expressed from the region have been measured during early and late stages of flower development in male, female, and hermaphrodite plants."

Results
The repetition of the goal "To investigate the structure and evolution of the SDR in Vitis spp." In line 132 can be omitted.

The sentence was modified.

Page 5 line 133:

"We sequenced and assembled the complete genomes of eight *Vitis* accessions, including three hermaphrodite *Vv vinifera* cultivars (Merlot, Black Corinth seedless and Black Corinth seeded), four *Vv sylvestris* accessions (two females and two males), and one male *V. arizonica*."

In line 136, the English is strange: "the genome of one male Muscadinia rotundifolia was constructed" means something like "the genome of a male Muscadinia rotundifolia was sequenced".

The sentence was modified as suggested.

Page 5 line 136:
"In addition, the genome of a male *Muscadinia rotundifolia* was sequenced as a dioecious outgroup to *Vitis spp.* (**Fig. 1c**; Small, 1903; Moore, 1991; Mullins *et al*., 1992; Liu *et al*., 2016; Wen *et al*., 2018; Zecca *et al*., 2020)."

Similarly, corrections are needed to the text "Each Vitis SDR haplotype was aligned to the Cabernet Sauvignon H haplotype to determine the structural differences among haplotypes [at this point, no structural differences have been mentioned, so readers don't know what this means, though perhaps it means simply to test for structural differences], and identify features that are conserved in a sex-specific manner" [maybe meaning identify sex-specific features"].

The sentence was modified.

Page 6 line 161:
"All SDR haplotypes were aligned to the Cabernet Sauvignon hap1 H haplotype to assess structural differences and to identify sex-specific features (**Fig. 2a-c**)."

Tenses are sometimes incorrect, for example in line 171 "These length differences reflected the presence of sex-linked SVs" (should read "reflect").

The sentence was modified.

Page 6 line 168:
"These length differences reflect the presence of sex-linked structural variants (SVs; > 50 bp)."

In addition, tenses in other sentences were corrected as suggested.

For example:

Page 4 line 86:
"It has been hypothesized that this region contains the recessive male-sterility and dominant female-sterility alleles predicted by the two-locus model, and their identification has been attempted by comparative gene expression analyses (Picq *et al*., 2014; Ramos *et al*., 2014)."

Page 4 line 107:

"For each genome, haplotypes within the genetically defined SDR have been curated manually, and transcripts expressed from the region have been measured during early and late stages of flower development in male, female, and hermaphrodite plants."

Page 4 line 110:
"With these extensive new sequence and expression data, we compare the F, H, and M haplotypes to better define the SDR, identify candidate sex-determining genes, and assess whether H haplotypes owe their origin to a recombination event."

Line 621 is an example of unnecessarily long-winded writing that is difficult to understand ("close proximity of sex-determining genes may serve as an impediment to their recombination", means "close linkage of sex-determining genes may simply reflect physical closeness").

The sentence was modified as suggested.

Page 18 line 582:
"The first is that the close linkage of sex-determining genes may simply reflect physical closeness (Oberle 1938)."

Several places have odd English, including "observations [or data] support that", which ought to be "support the view [or interpretation, or working hypothesis] that"

The manuscript was edited to resolve these issues.

Page 18 line 557:
"Consistent with this conjecture, several pieces of evidence support the hypothesis that H haplotypes arose from a recombination event, including their intermediate length and their similarity in structure and sequence to F haplotypes in the first portion of the SDR and to M haplotypes in the latter portion of the SDR (**Fig. 6a**)."

Page 18 line 561:
"Phylogenetic evidence supports the hypothesis that the recombination event occurred between the *aldolase* and *TPP* genes, and polymorphism information supports that it could have occurred within *TPP* gene, because the gene sequence contained both M- and F-linked non-synonymous SNPs (**Fig. 3a-f**; **Fig. 6b**)."

Reviewer #3 (Remarks to the Author):

The authors significantly improved the article. For our part we are satisfied.
Just a small detail that we forgot in the previous review: the authors refer to the existence of three sexes, (pag.3 line 70 and pag.4, line 75).
Please consider: in our world there are only two sexes: male and female. Nowadays this may be a philosophical question, but as we are talking about flowers perhaps the designation of "flower type" is appropriate, when you need to refer male, female and hermaphrodite. And so, we have two sexes but three flower types.

We thank the reviewer for this comment. Accordingly, sentences were modified.

Page 3 line 69:
"Therefore, *Vitis* spp. have individuals of three types (Negi and Olmo, 1970; **Fig. 1a,b**): (i) males with flowers that have reduced pistils, with neither stigma nor style development, (ii) females with flowers containing reflexed anthers and stamens that release sterile pollen grains (Gallardo *et al.*, 2009), and (iii) hermaphrodites within *Vv vinifera*, which have perfect flowers with functional pistils and stamens that bear fertile pollen."

Page 3 line 74:
"The three types are determined by the genotype at the SDR locus."

Reviewer #4 (Remarks to the Author):

I thank the authors for accurate answers and the changes in the text. In my opinion, the authors have sufficiently addressed the revisions and submitted information to include from the previous draft. I consider that this manuscript provides new and interesting results and thus I suggest publishing.

REVIEWERS' COMMENTS:

Reviewer #2 (Remarks to the Author):

Many problem places in the ms have now been corrected.
